# Transcriptome Analysis of *Aedes aegypti* Transgenic Mosquitoes with Altered Immunity

**DOI:** 10.1371/journal.ppat.1002394

**Published:** 2011-11-17

**Authors:** Zhen Zou, Jayme Souza-Neto, Zhiyong Xi, Vladimir Kokoza, Sang Woon Shin, George Dimopoulos, Alexander Raikhel

**Affiliations:** 1 Department of Entomology and the Institute for Integrative Genome Biology, University of California, Riverside, California, United States of America; 2 W. Harry Feinstone Department of Molecular Microbiology and Immunology, Bloomberg School of Public Health, Johns Hopkins University, Baltimore, Maryland, United States of America; Case Western Reserve University, United States of America

## Abstract

The mosquito immune system is involved in pathogen-elicited defense responses. The NF-κB factors REL1 and REL2 are downstream transcription activators of Toll and IMD immune pathways, respectively. We have used genome-wide microarray analyses to characterize fat-body-specific gene transcript repertoires activated by either REL1 or REL2 in two transgenic strains of the mosquito *Aedes aegypti*. Vitellogenin gene promoter was used in each transgenic strain to ectopically express either REL1 (REL1+) or REL2 (REL2+) in a sex, tissue, and stage specific manner. There was a significant change in the transcript abundance of 297 (79 up- and 218 down-regulated) and 299 (123 up- and 176 down-regulated) genes in fat bodies of REL1+ and REL2+, respectively. Over half of the induced genes had predicted functions in immunity, and a large group of these was co-regulated by REL1 and REL2. By generating a hybrid transgenic strain, which ectopically expresses both REL1 and REL2, we have shown a synergistic action of these NF-κB factors in activating immune genes. The REL1+ immune transcriptome showed a significant overlap with that of *cactus* (RNAi)-depleted mosquitoes (50%). In contrast, the REL2+ -regulated transcriptome differed from the relatively small group of gene transcripts regulated by RNAi depletion of a putative inhibitor of the IMD pathway, caspar (35 up- and 140 down-regulated), suggesting that caspar contributes to regulation of a subset of IMD-pathway controlled genes. Infections of the wild type *Ae. aegypti* with *Plasmodium gallinaceum* elicited the transcription of a distinct subset of immune genes (76 up- and 25 down-regulated) relative to that observed in REL1+ and REL2+ mosquitoes. Considerable overlap was observed between the fat body transcriptome of *Plasmodium*-infected mosquitoes and that of mosquitoes with transiently depleted *PIAS*, an inhibitor of the JAK-STAT pathway. *PIAS* gene silencing reduced *Plasmodium* proliferation in *Ae. aegypti*, indicating the involvement of the JAK-STAT pathway in anti-*Plasmodium* defense in this infection model.

## Author Summary

Mosquito-borne diseases cause tremendous morbidity and mortality worldwide. New approaches to control vector-borne diseases include interruption of the association between pathogens and vectors by genetic manipulation of vectors and the development of transmission-blocking vaccines. Potential success of these approaches requires in-depth knowledge of the molecular interactions between vector defense mechanisms and the ability of a pathogen to overcome these defenses. A combination of the genome-wide microarray and transgenic approaches has permitted us to decipher repertoires of genes controlled by two major immune pathways, Toll and IMD, in the Dengue-fever mosquito vector *Aedes aegypti*. We have shown that these pathways interact to bring about a high level of immune genes by means of generating a transgenic strain, which ectopically expresses the NF-κB factors of TOLL and IMD. In *Ae. aegypti*, a malaria parasite *Plasmodium gallinaceum* elicited the transcription of a distinct subset of immune genes relative to those observed in transgenic mosquitoes with activated Toll or IMD pathways. However, a considerable overlap was observed between the fat body transcriptome of *Plasmodium* infected mosquitoes and that of mosquitoes with the activated JAK-STAT pathway. *Plasmodium* proliferation was reduced in the latter, indicating JAK-STAT involvement in anti-*Plasmodium* defense in this infection model.

## Introduction

Mosquito-borne diseases cause tremendous morbidity and mortality worldwide [1]. New approaches to control vector-borne diseases include interruption of the association between pathogens and vectors by genetic manipulation of vectors and the development of transmission-blocking vaccines. Potential success of these approaches requires in-depth knowledge of the molecular interactions between vectors' defense mechanisms and the evolutionary established ability of a pathogen to overcome these defenses.

The yellow fever mosquito *Aedes aegypti* is the principal vector of Dengue fever and, due to a large body of knowledge amassed for this mosquito and readily available genetic and molecular tools, it also serves as an outstanding model for vector biology [2]. Sequencing and annotation of the genome of this mosquito have been critical in further advancing genomic and molecular approaches in studies of its immunity [3], [4]. Despite being five times larger than the genome of the malaria mosquito *Anopheles gambiae*, the *Ae. aegypti* genome consists of a similar number of protein-encoding genes, around 17,700 [3], [4]. Comparative genome analysis has indicated that 353 *Aedes* genes from 31 families are associated with immunity, compared with 285 and 338 immune genes in *Drosophila melanogaster* and *An. gambiae*, respectively, suggesting expansions of some immune gene groups in *Ae. aegypti* [4]. The key immune pathways are conserved between mosquitoes and the fruit fly; however, mosquitoes exhibit expansions of pattern recognition and effector molecules, likely due to their co-evolution with various pathogens [4]. Similar to *Drosophila*, mosquito Toll and IMD pathways constitute major immune pathways activating a battery of anti-microbial peptides and immune proteins in response to invasion by various microorganisms [4], [5]. The activation of genes encoding these immune effector molecules is accomplished by the action of the NF-κB transcription factors REL1, the orthologue of *Drosophila* Dorsal, and REL2, the Relish orthologue, respectively [6], [7], [8].

Another important defense mechanism in Arthropods is melanization, which mediates wound healing and parasite encapsulation [9]. The key enzyme of melanization, phenoloxidase (PO), is involved in the production of toxic melanin, which is deposited at the wound or around the parasite. A CLIP-domain serine protease cascade is responsible for amplification of signals, which are released upon infection, from wounded tissues or ruptured oenocytoids, and conversion of prophenoloxidase (PPO) into an active PO. (Reviewer 1, query 3) The activation of the melanization cascade is under strict regulation by serine protease inhibitors (serpins). The importance of melanization cascades in mosquitoes is indicated by major expansions in their melanization pathway gene families (10 PPOs, 25 Serpins, and 79 CLIPs in *Ae. aegypti*) [4], [10], [11].

The fat body of insects, such as *Drosophila* and mosquitoes, is the major metabolic tissue, and also serves as a powerful immune organ [5], [12]. Although the role of the fat body in immunity has been demonstrated for the model insect *Drosophila* [5], its precise function in immune responses in mosquitoes is still largely unknown. Deciphering the repertoire of immune genes expressed in the mosquito fat body is of particular importance because of the considerable expansion of immune-related genes in mosquitoes relative to that in *Drosophila* [4], [10], [11].

In previous studies, we have generated transgenic strains of *Ae. aegypti*, in which REL1 and REL2 were ectopically expressed under the control of the blood-meal-regulated promoter of the vitellogenin (*Vg*) gene in the fat body [13], [14]. In this current work, we took advantage of the availability of these transgenic strains and performed transcriptome analyses to characterize repertoires of fat body-specific genes controlled by Toll and IMD pathways in this vector. Using microarray-based genome-wide transcriptional analyses, we have characterized gene repertoires in two transgenic *Ae. aegypti* mosquito strains that ectopically express either REL1 (REL1+ strain) or REL2 (REL2+ strain). Moreover, we have shown a synergistic action of REL1 and REL2 in activating immune genes in the transgenic mosquito co-expressing both these NF-κB transcription factors. Infection of *Ae. aegypti* with *Plasmodium gallinaceum* resulted in the transcriptional modulation of a distinct subset of host immune genes. There was considerable overlap between the fat body transcriptome of *Plasmodium*-infected mosquitoes and the repertoire of genes regulated in mosquitoes transiently depleted of PIAS, an inhibitor of the JAK-STAT pathway. RNAi depletion of PIAS reduced *Plasmodium* proliferation in *Ae. aegypti*, indicating involvement of JAK-STAT in anti-parasite defense.

## Results/Discussion

### Fat body transcriptional responses in REL1+ and REL2+ transgenic *Aedes aegypti* female mosquitoes

Previously generated transgenic strains of the mosquito *Ae. aegypti* ectopically expressing either *REL1* or *REL2* [13], [14] have permitted us to decipher transcript repertoires of genes in the fat body controlled by the Toll and IMD pathways, respectively. We analyzed the transcriptional profiles of fat body-expressed genes using custom-made 60-mer oligonucleotide microarrays representing the approximately 17,700 *Ae. aegypti* genes [15]. The transgenic mosquitoes were constructed to ectopically express either recombinant REL1 or REL2 using the *Vg* promoter, which is a female- and fat-body-specific, blood meal-inducible gene [16]. The abundance of transcripts in REL1+ and REL2+ mosquitoes was compared with that in the non-transgenic wild type mosquitoes at 24 h post blood meal (PBM), and genes uniquely regulated by REL1+ and REL2+ mosquitoes were further analyzed. The time point of 24 h PBM was chosen for transcriptome analyses because it is the expression peak for the *Vg* gene, whose upstream regulatory region was used to drive the expression of both *REL1* and *REL2* transgenes. The *REL1* transgene is maximally expressed in the fat body of the REL1+ transgenic strain at this PBM time [13]. We reexamined *REL2* transgene expression profile in the REL2+ strain, reported in [14], by means of quantitative real time PCR (qRT-PCR) and found that its peak was at 24 h PBM (Figure S1).

**Figure 1:**
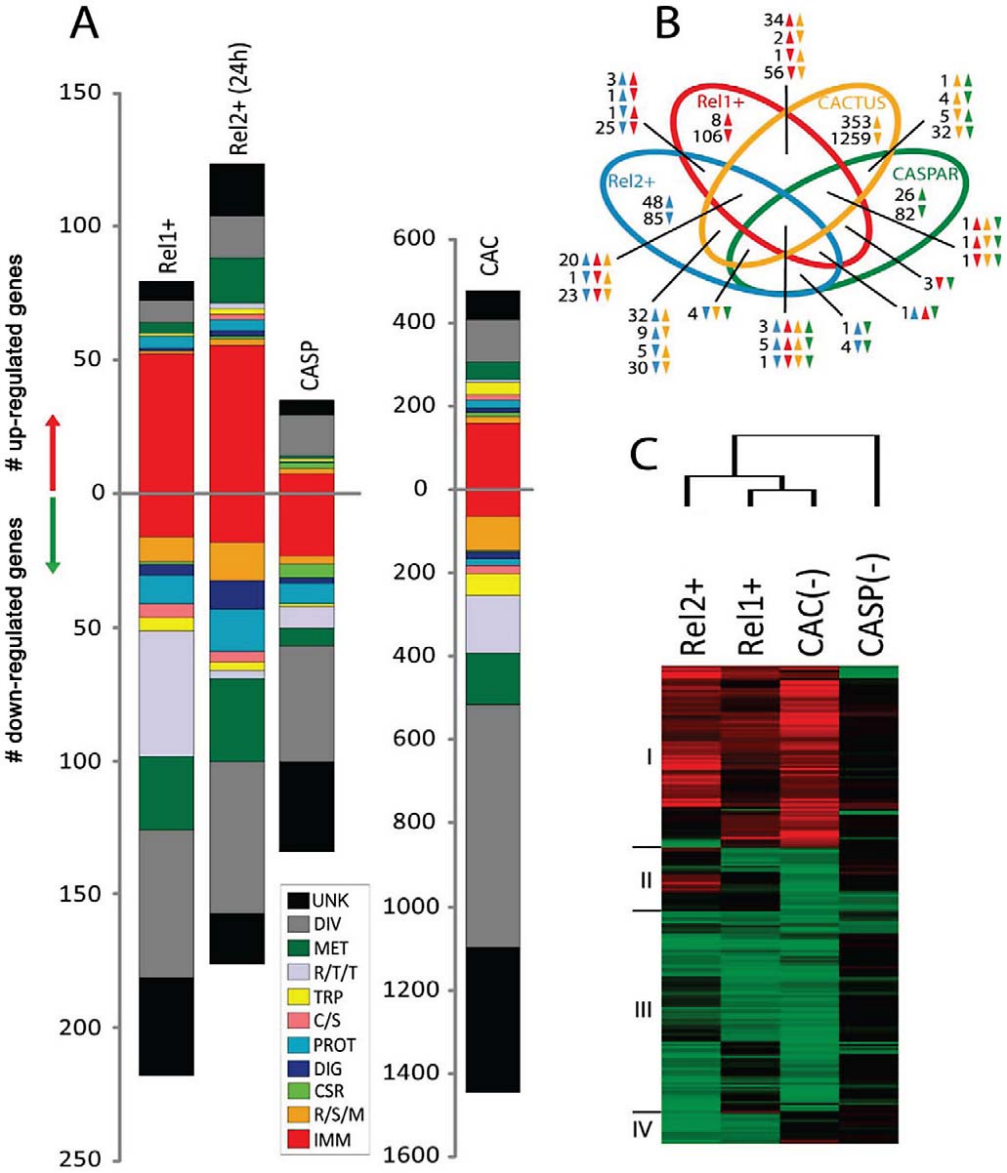
Comparative transcriptome analysis of fat body genes in REL1+ and REL2+ transgenic mosquitoes. **A**) Functional classification of the REL1+ and REL2+ regulated transcriptome. Functional group abbreviations are: IMM, immunity; R/S/M, redox, stress and mitochondrion; CSR, chemosensory reception; DIG, digestive; C/S, cytoskeletal and structural; PROT, proteolysis; TRP, transport; R/T/T, replication, transcription, and translation; MET, metabolism; DIV, diverse functions; UNK, unknown functions. **B**) Venn diagram representing unique and shared transcriptome regulation in REL1+ and REL2+ transgenic mosquitoes, and *cactus*- and *caspar*-depleted mosquitoes. The overlapping regions represent genes that are concomitantly regulated in two, three, or four experimental conditions at the level of transcript abundance. The direction of gene transcript changes is indicated by upward-pointing and downward-pointing arrows. Green, Brown, Red, and Blue colors represent *caspar*-depleted, *cactus*-depleted, REL1+, and REL2+ transgenic mosquitoes, respectively. **C**) Hierarchical cluster analysis of fat body gene transcripts that were significantly regulated in at least two of the four experimental conditions; REL1+, REL2+, *cactus*- and *caspar*-depleted mosquitoes.

The fat body transcriptome of REL1+ transgenic mosquitoes contained 297 gene transcripts, 79 of which were up-regulated and 218 down regulated (Figure 1A). Immune genes were the most predominant up-regulated group in the REL1+ fat body transcriptome, representing 66% of all up-regulated genes (Figure 1A and Table S1). Among the category of immune genes that were up-regulated in the REL1+ mosquito fat body were components of the Toll pathway, indicating the involvement of REL1 in the feedback regulation of its own pathway. These were genes encoding the Toll-specific pattern recognition receptor, the Gram Negative Binding Protein 1 (*GNBP1*), *spätzle 3A*, *REL1*, and the negative regulator of the Toll pathway, *cactus* (Table S1). Activation of effector molecule transcripts–the anti-microbial peptides (AMPs) *defensins A*, *C* and D, and *lysozymes C10* and *C11*–was high (Table S1). Defensins represent the major antimicrobial peptides (AMP) in mosquitoes [4].

Genes encoding opsonization factors, such as thio-ester proteins (TEP)–*TEP2*, *3*, *20*, *21*, and *22*–represented another predominant group of REL1-induced immune genes. Member of the TEP family have been identified in diverse animal species and play important roles in immune responses as components of the complement system [17]. In *An. gambiae*, hemocyte-specific TEP1 has been implicated as a key molecule involved in killing of midgut stages of Plasmodium [18]. It acts with two leucine-rich repeat (LRR) proteins, LRIM1 and APL1, as a complement system in parasite killing [19]. However, functions of most TEPs in insects, including mosquitoes, remain to be elucidated.

Transcripts of 6 genes, encoding galactose-specific C-type lectins were also elevated in the REL1+ transcriptome. Genes encoding proteolytic cascades and signaling modulators, *CLIPs* and *serpins*, were also represented in the immune repertoire of the REL1+-induced fat body transcriptome (Table S1). Previously, we have shown that some of these gene transcripts, *TEP15*, *TEP20*, *defensin A*, and *CLIP13B*, were activated by REL1, thus, providing additional confidence to our genome-wide transcriptome data set [7].

Transcript of the gene encoding an orthologue of the *Drosophila* JAK/STAT pathway receptor Domeless (Dome) was among the most highly increased upon REL1 activation in the fat body (AAEL012471), indicating the involvement of Toll-REL1 pathway in regulating JAK-STAT pathway (Table S1). Dome is the *Drosophila* homolog of the vertebrate transmembrane cytokine class I receptor, which serves as a signal transducer and mediates activation of *totA* in the fat body [20], [21]. Activation of Dome/JAK/STAT signaling requires hemocyte-specific cytokine Unpaired [21]. Fat body *totA* is also regulated by Relish, a *Drosophila* orthologue of mosquito REL2. Here, we provide evidence on the involvement of REL1 in up-regulation of *Dome*, the gene encoding a key component of the JAK/STAT pathway in the mosquito fat body.

Several recent studies in *Drosophila* have pointed out on a communication between immune tissues[21], [22], [23], [24]. In addition to hemocyte-specific cytokine mediated activation of the Dome/JAK/STAT in the fat body, blood cells are also required for the immune activation of the fat body [22], [24]. However, Rel proteins, Dif and Dorsal, also act in the fat body to produce factors that promote blood-cell number in *Drosophila* larvae [23]. The identity of these fat body factors remains undetermined. There is no evidence of possible involvement of Relish in similar fat body – blood cell communication. Utilization of fat body-specific, ectopically expressed REL1+ represents a unique opportunity to address the question about fat body – hemocyte communication in mosquitoes. Thus, although the ectopic expression of REL1 in the REL1+ transgenic mosquitoes is strictly fat body-specific, the fat body REL1-mediated production of blood cell stimulating factors could activate proliferation of blood cells adhered to fat body preparations. As a consequence, the overall transcriptome from REL+ mosquitoes could include genes from proliferating hemocytes. This aspect of fat body – blood cell communication will be studied further in the future.

The REL1+ controlled transcriptome contained a large number of genes (230) attributed to non-immune biological processes; 26.6% of these gene transcripts (REL1) were involved in ribosomal biogenesis, DNA replication and metabolism (Figure 1A; Table S1). 88% of these non-immune genes were down-regulated. Notably, 44 of down-regulated genes were related to ribosomal biogenesis and translation. This observation is in agreement with microarray analyses of ectopic expression of Rel proteins in Drosophila [25].

One of genes activated in the REL1+ fat body transcriptome encodes an orthologue of a vertebrate Grb2-associating protein (Gasp, AAEL002492; Table S1), which is a thymus-specific factor critical for T-cell differentiation [26]. Finding its function represents a potentially important aspect of immunity in the mosquito.

REL1 also up-regulates an orthologue of the cytosolic sulfotrnasferase, SULT (AAEL006334, Table S1). Members of SULT superfamily catalyze the sulfation of xenobiotics, hormones and neurotransmitters [27]. Considering multiple functions of these enzymes, it is difficult to predict the role of this REL1-dependent SULT in the mosquito fat body.

An interesting glimpse in the gene functional conservation is also provided by the Rel-mediated up-regulation of an orthologue of a vertebrate major facilitator superfamily domain-containing protein (Mfsd2a, AAEL009195; Table S1), which is expressed in brown adipose tissue and liver (the fat body is a functional analogue of these tissues combined) [28]. In vertebrates, Mfsd2a is highly expressed during thermogenesis and have been found to be a tumor suppressor [28], [29].

The fat body transcriptome of REL2+ transgenic mosquitoes contained 299 genes, 123 of which were up-regulated and 176 down regulated (Figure 1A and Table S2). Immune genes represented 44% of most highly up-regulated genes in the REL2+ fat body transcriptome. In particular, transcripts of AMPs and recognition molecules were enriched (Table S2). Genes encoding thio-ester proteins (TEP)–*TEP20*, *21*, and *22*–were also elevated among REL2-induced immune genes. Genes encoding factors of the IMD pathway–a peptidoglycan recognition protein (*PGRP-S1*) and *REL2* - were up-regulated (Figure 1A and Table S2).

Two members of the APL1 family of leucine-rich (LRR) proteins, APL1B (AAEL012086) and APL1C (AAEL009520), were up-regulated in the REL2+ fat body transcriptome but not in the REL1+ one (Tables S1 and S2). LRR proteins play an important role in the innate responses against pathogens in plants, insects, and mammals [30], [31], [32]. APL1 (*Anopheles Plasmodium*-responsive leucine-rich repeat 1) was first identified in *An. gambiae*, in which it controls resistance to *Plasmodium falciparum* [33]. The APL1 family is comprised of paralogs APL1A, APL1B and APL1C [34]. APL1C is responsible for defense of *An. gambiae* against *P. berghei*, which is a rodent parasite. APL1C has been reported to function within the REL1-cactus immune signaling, which regulates APL1C at the transcriptional and translational levels [34]. However, further studies have revealed that protection of *An. gambiae* against its natural parasite *P. falciparum* is mediated by APL1A [35]. This protection correlates with the transcriptional control of APL1A by REL2, suggesting that REL2 anti-parasite phenotype results partially from its control of APL1A [35]. APL1C has been implicated in a complement-like pathway that mediates parasite killing interacting with LRIM1 and TEP1 [36], [37]. Our data indicate that *Ae. aegypti* APL1 proteins are controlled by REL2.

Up-regulation of a fibrinogen-related protein (AAEL004150) was also observed in the REL2+ fat body transcriptome. The fibrinogen-related gene family belongs to pattern recognition receptors and involved in innate immunity in both invertebrates and vertebrates [38], [39], [40]. In *An. gambiae*, fibrinogen-related proteins interact with Gram-positive, Gram-negative bacteria and co-localized with both *P. berghei* and *P. falciparum* [40]. It has been suggested that fibrinogen-related proteins expand pattern recognition capacity, thus, enhancing innate immunity against various pathogens.

227 genes in the REL2+ fat body transcriptome belonged to genes encoded factors of non-immune biological processes. Transcript levels of some genes related to non-immune functional categories, most notably stress and metabolism, were predominantly repressed (Figure 1A; Table S2). Thirty-three genes (6 induced and 27 repressed) in the REL2+ regulated transcriptome were related to proteolysis process. Interestingly, REL2+ transcriptome contained an up-regulated component of ribosome biogenesis, 20S rna accumulation protein 1 (AAEL004493), in contrast to overall down-regulation of genes related to ribosomal biogenesis and translation in the REL1 transcriptome. This difference points out on specificity of action of REL1 and REL2 not only in affecting immune, but also non-immune genes.

We also compared the fat body transcriptomes of REL1+ and REL2+ transgenic mosquito with those of mosquitoes in which either the negative regulator cactus or caspar had been depleted by RNAi silencing. The latter two transcriptomes have been previously reported and are represented here for comparative purposes only [15]. Cactus is a repressor of *Drosophila* Dorsal/Dif and mosquito REL1, which has been shown to directly interact with this NF-κB factor preventing the latter to translocate to the nucleus [5], [15], [41], [42]. In mosquitoes, *cactus* silencing results in activation of *REL1* and its underlying immune responses [7], [10], [11], [15], [41]. Hierarchical clustering confirmed the close relationship between the immune transcriptomes regulated by transgene *REL1* overexpression and *cactus* depletion (Figure 1C, Cluster I and Table S4). However, REL1+ affected transcript abundance of fewer genes in diverse functional classes compared to *cactus* depletion. Our analysis revealed the presence of the same 53 genes in transcriptomes from REL1+, REL2+ transgenic and *cactus*-depleted mosquitoes (Figure 1B). 30 of them belonged to immunity category.

In *Drosophila* and *Anopheles*, caspar has been shown to be an inhibitor of the IMD pathway, in which it has been suggested to prevent Dredd-dependent nuclear translocation of Relish and REL2 [43], [44]. In *Ae aegypti*, RNA depletion of *caspar* triggered up-regulation of only a small number of genes when compared with *REL2* transgene ectopic expression ([15] and this report). Moreover, caspar-induced transcriptome only marginally overlapped with that of REL2+ (Figure 1C and Table S4). Further studies are required to clarify the role of caspar in the regulation of the IMD pathway.

### Synergistic action of REL1 ad REL 2 in activating immune genes in the mosquito fat body

Importantly, 84 genes were present in both REL1+ and REL2+ fat body transcriptomes, suggesting co-regulation of these genes by the NF-kB factors REL1 and REL2 and their respective pathways (Figure 1B and Figure S2, Table S3). The majority of highly enriched gene transcripts (50%), which were common for both REL1+ and REL2+ fat body transcriptomes, belonged to the immunity category. The AMP genes *defensins A*, *C*, *D* and *lysozyme C* displayed increased mRNA abundance in response to either REL1 or REL2. However, REL2 appeared to be a more potent activator of these AMPs. A group of six galactose-specific C-type lectin transcripts was highly elevated in both transcriptomes. Gene transcripts encoding TEPs, *TEP2*, *TEP20*, *TEP21* and *TEP22*, appeared to be equally upregulated by REL1 and REL2.

Only 22% of all non-immune genes were present in both REL1+ and REL2+ -regulated fat body transcriptomes in contrast of 50% of immune ones. Among non-immune genes that were induced in both transgenic mosquitoes was juvenile hormone esterase (JHE). Increased JHE activity has been linked with degradation of juvenile hormone during PBM development in *Ae aegypti* females [45]. However, modulation of juvenile hormone titer via immune factors has not been previously reported. The majority of non-immune down-regulated genes in both transcriptomes belonged to metabolism and cell cycle functional categories (Table S3).

**Figure 2:**
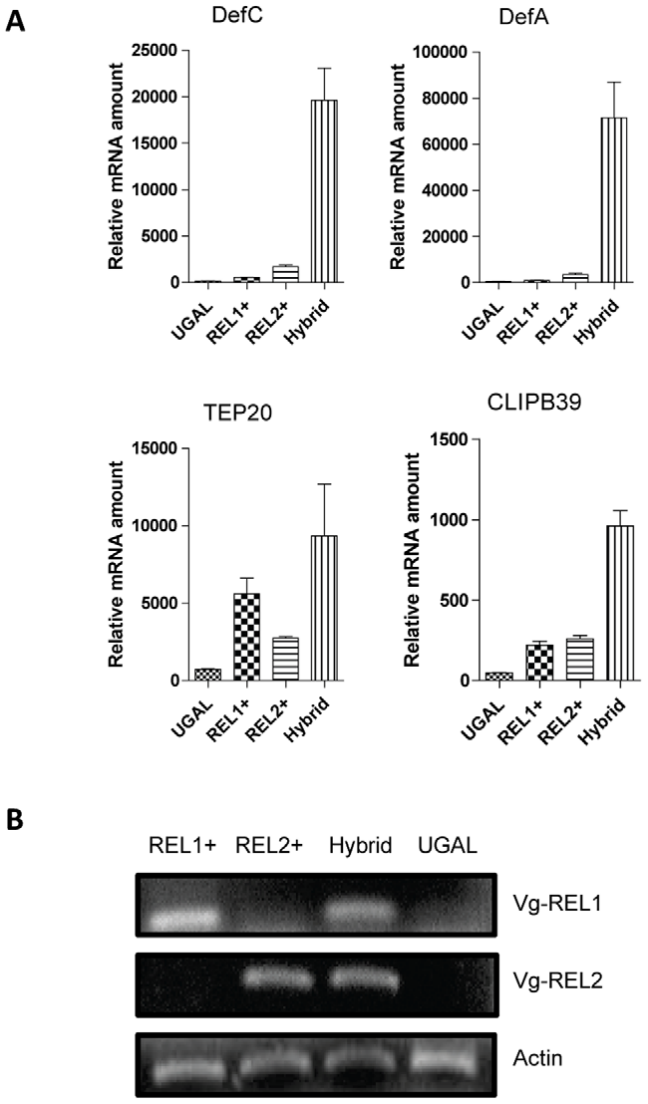
Synergistic action of REL1 and REL2 in activating immune genes. **A**) Transgenic mosquitoes with over-expressed *REL1* or *REL2* were used to generate hybrid transgenic mosquitoes over-expressing both of these factors. Fat bodies of REL1+, REL2+, and RE1+/REL2+ mosquitoes were dissected 24 h PBM and analyzed by means of quantitative RT-PCR for selected immune genes. A. Defensin A; B. Defensin C; C. TEP 20; D. CLIPB39. UGAL was used as a control. **B**) Both *Vg-REL1* and *Vg-REL2* transgenes were expressed in the hybrid mosquitoes. Total RNA isolated from females 24 h PBM were analyzed using specific primers for vitellogenin that recognize exclusively hybrid transgene mRNA. RT-PCR analysis indicated that both *Vg-REL1* and *Vg-REL2* transgenes were expressed in the hybrid mosquitoes. The same RNA samples were tested using actin specific primers as controls.

To decipher whether genes represented in both REL1+ and REL2+ fat body transcriptomes were synergistically regulated by these NF-κB transcription factors, we generated a hybrid REL+/REL2+ transgenic mosquito strain by crossing REL1+ and REL2+ strains. Unlike parental RE11+ and REL2+ strains, the hybrid REL1/REL2 mosquitoes carried both *Vg-REL1* and *Vg-REL2* transgenes, which were ectopically expressed after a blood meal specifically in female fat bodies (Figure 2). We analyzed transcript abundance of selected genes representing the functional group of 33 immune genes upregulated in both REL1+ and REL2+ transcriptomes. REL1+, REL2+ and REL1+/REL2+ hybrid transgenic mosquitoes were blood fed, RNA was isolated from their fat bodies 24 h PBM and subjected to qRT-PCR analysis. This analysis revealed that the transcript levels of Defensin A, Defensin C, CLIPB39, and TEP20 genes in the REL1+/REL2+ hybrid transgenic female mosquitoes was considerably higher as compared to those in either REL1+ or REL2+ strains (Figure 2). *Defensin A* and *Defensin C* were particularly elevated in the REL1+/REL2+ hybrid mosquitoes (Figures 2A and 2B). A predominant concept in insect immunology is that Toll and IMD pathways act independently, with the Toll pathway responding to fungi and Gram-positive bacterium-derived Lys-type peptidoglycan and the IMD pathway to Gram-negative bacterium-derived diaminoacipimelic acid (DAP)-type peptidoglycan, with each pathway activating a separate set of effector genes [5], [42]. However, the transcriptome analyses of double *Drosophila* mutants of the Toll and IMD pathways have revealed some co-regulated antimicrobial peptides [46]. Regulation of *Drosophila* antifungal AMP Drosomycin mainly depends on Toll pathway and receives a modest input from IMD during a systemic immune response; though, the IMD pathway solely activates *Drosomycin* and *Diptericin*, respective target genes of the Toll and IMD pathways, in the local immune response.[47], [48]. Tanji et al. [49] have shown the synergistic action of Toll and IMD pathway in activating *Drosomycin* and *Diptericin*. Moreover, DIF and Relish form heterodimers to regulate antimicrobial peptides in *Drosophila* [50]. Our transgenic approach strongly suggests the synergistic action of REL1 (an orthologue of Dorsal) and REL2 (an orthologue of Relish) in activation of immune genes in the mosquito *Ae. aegypti*. The high level of up-regulation of immune genes in REL1+/REL2+ hybrid mosquitoes as co-expression of *Vg*-driven REL1 and REL2 clearly indicated the synergy of interaction between these NF-kB factors. Comparable levels of individual ectopic expression of each REL factor in a respective transgenic REL strain did not elicit similarly high up-regulation of immune factors, assayed in this experiment.

Previously, we have shown that simultaneous ectopic expression of *Defensin A* and *Cecropin A* in *Ae. aegypti* leads to a complete elimination of the malaria parasite *P. gallinaceum* and interruption of its transmission in transgenic hybrid CecA/DefA mosquitoes [51]. Ectopic co-expression of REL1 and REL2 in a sex-, tissue- and stage-specific manner that elicits a strong synergistic effect on activation immune factors provides a potent method to study mosquito and pathogen interaction.

### Control of melanization-related genes by REL1 and REL2

Microarray-based transcriptome analysis was used to study the effect of immune signal transduction pathways on melanization-related gene expression in the *Ae. aegypti* fat body. At 24 h PBM, 21 melanization-related genes were significantly upregulated in the fat body of transgenic REL1+ female mosquitoes, while 24 (22 up- and 2 down-) genes were controlled by REL2 ectopic expression in the same tissue (Figure S2, Table S5). CLIP-domain serine proteases can be separated into five subfamilies, among which CLIPA, B, and C are implicated in the activation of melanization. The CLIPA subfamily is composed of the non-catalytic clip domain serine protease homologues, which contain imperative PPO activation cofactors. Mosquito CLIPA14 and CLIPA6 are homologous to the PPO activation cofactors, *Manduca sexta* SPH1, SPH2 (serine protease homologue), and *Holotrichia diomphalia* PPAF2 (PPO activating factor) [52]. CLIPA1 and CLIPA11 were up-regulated in both REL1+ and REL2+. However, CLIPA5, CLIPA6, and CLIPA16 were induced only in the REL1+, while CLIPA14 was enriched in the REL2+. Two melanization proteases (CLIPB39 and CLIP40) [11] and CLIPB79 were induced in both REL1+ and REL2+ mosquitoes. Of all the CLIPC, D, and E subfamilies, only a single gene, CLIPE8, was induced in REL2+ (Table S5).

REL1 and REL2 differently regulated transcription of genes encoding Serpins. Serpins-9, −16, −4B, −4C were up-regulated only in the REL1+ mosquitoes, while Serpins-2, −11, and −23 mRNAs in REL2+ mosquitoes (Table S5). Serpins-1, −8, and, −16 mRNAs were elevated in both REL1+ and REL2+ mosquitoes (Table S5). In *Ae. aegypti*, Serpin 1 is involved in control of immune melanization, while Serpin-2 in tissue melanization, exemplified by the formation of melanotic tumors after RNAi Serpin-2 depletion [11]. *PPO* gene transcripts were not detected in either REL1+ or REL2+ fat-body-specific transcriptomes. This is in agreement with previous data showing that *PPO* genes are expressed in hemocytes in both *Drosophila* and mosquitoes [5], [53].

### Transcriptional responses triggered by *Plasmodium gallinaceum* in *Ae. aegypti*

**Figure 3:**
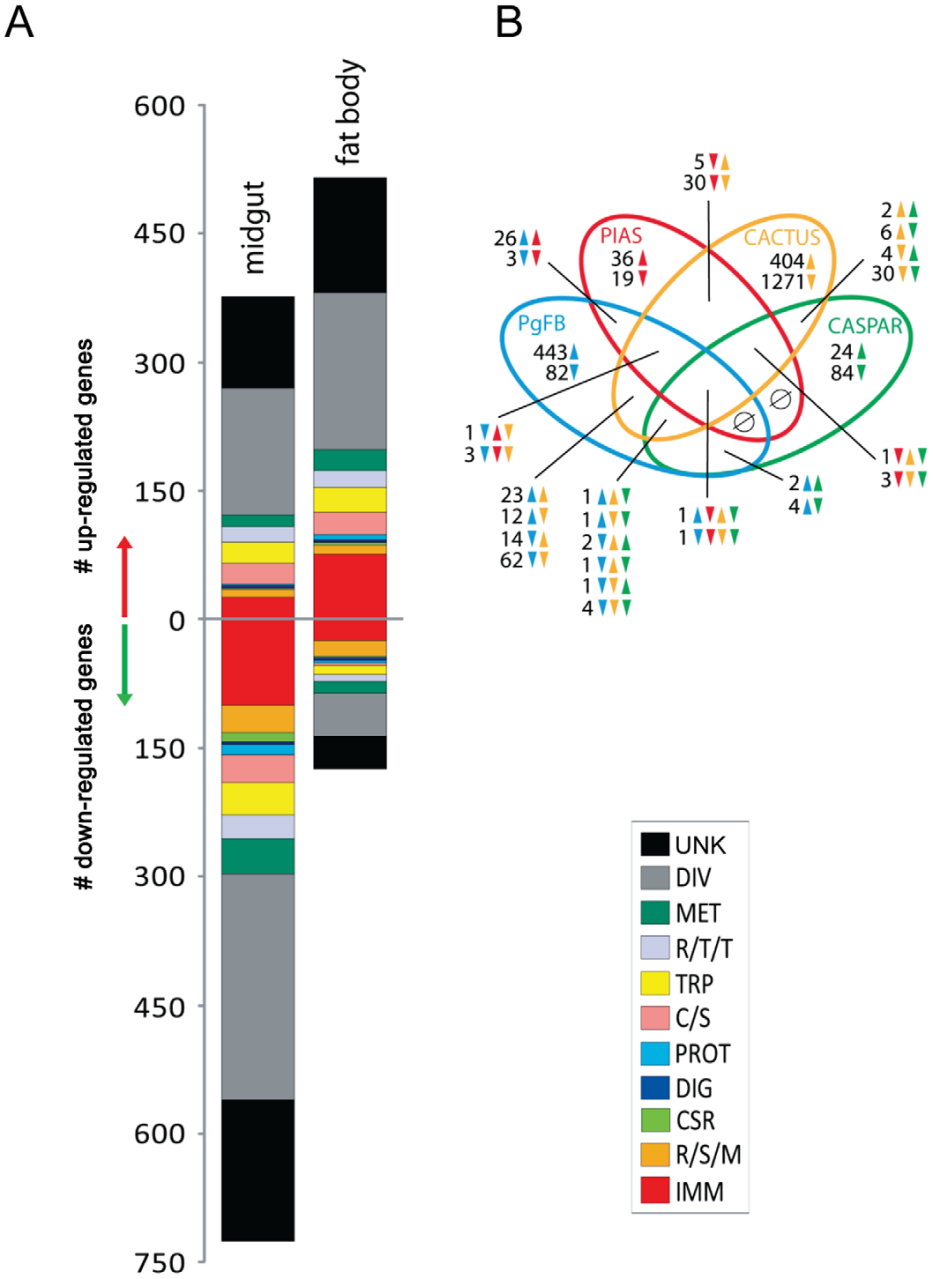
Comparative analysis *P. gallinaceum* midgut- and fat body- responsive transcriptomes. **A**) Functional classification of the PgMG- and PgFB-regulated transcriptomes. Functional group abbreviations are: IMM, immunity; R/S/M, redox, stress and mitochondrion; CSR, chemosensory reception; DIG, digestive; C/S, cytoskeletal and structural; PROT, proteolysis; TRP, transport; R/T/T, replication, transcription, and translation; MET, metabolism; DIV, diverse functions; UNK, unknown functions. **B**) Venn diagram representing unique and shared transcript regulation in PgMG, PgFB, and *cactus*- and *caspar*-depleted mosquitoes. The overlapping regions represent genes that are concomitantly regulated in two, three, or four experimental conditions at the level of transcript abundance. The direction of gene transcript regulation is indicated by upward-pointing and downward-pointing arrows. Green, Brown, Red, and Blue colors represent *caspar*-depleted, *cactus*-depleted, *PIAS*-depleted, and *Plasmodium*-infected mosquitoes, respectively.

To assess the relationship of REL1+ and REL2+ transcriptomes with transcriptional responses induced by *Plasmodium* infection in the mosquito *Ae. aegypti*, we compared transcriptomes of mosquitoes fed on a *Plasmodium*-infected blood meal with those fed on a non-infectious blood meal. In both the midgut and fat body, the majority of genes regulated in the presence of *Plasmodium* belonged to diverse or unknown functional classes, defined as such because of insufficient information for assigning particular known functions. The number of genes that significantly changed their transcript levels after *Plasmodium* infection in the midgut was almost twice higher than that in the fat body; impressively, *Plasmodium* infection envoked the induction of 375 genes and repression of 724 genes in the midgut, while 513 genes were induced and 174 genes were repressed in the fat body (Figure 3, Tables S6 and S7). The quantitative RT-PCR used to verify transcripts levels for 23 genes (12 from PgFB and 11 from PgMD) showed a high degree of correlation (best-fit linear-regression R^2^ = 0.77) with the microarray transcriptome data (Figure S3). The immune-related genes were the third most-represented functional gene group in both the midgut (124 genes) and the fat body (99 genes) transcriptomes in *Plasmodium*-infected mosquitoes (Figure 3).

In the midgut, transcriptional responses affected by *Plasmodium* infection were marked by a significant down-regulation of immune gene transcripts (99 out of 124 regulated immune genes). Among these down-regulated immune genes were several serine proteases (SPs), CLIP-domain serine proteases, Serpins (*serpins-4A*, *−7*, *−11*, *−17*, *−20* and *−21*), *lysozyme C*, various PRR molecules such as the PGRP proteins (*PGRP-LA*, *PGRP-LD*, and *PGRP-SC2*), *GNBP1*, fibrinogen-related proteins (*FBN12*, *FBN12*, *FBN13*, *FBN18*, *FBN24*, and *FBN27*), three AMPs (*defensins A* and *D*, *cecropin D*) (Table S6). Majority of genes putatively related to the melanization cascade were down-regulated in the midgut (26 down-regulated and only 4 up-regulated). The transcript abundance of CLIPA5, CLIPA6, CLIPB13A, CLIPB13B, CLIPB5 CLIPA3, CLIPB1, and Serpin-11 mRNAs were reduced in *Plasmodium* infected midgut (Table S6). CASPS18 was upregulated, while CASPS7 and CASPS20 were downregulated. 32 out of 40 genes related to oxidative stress were down-regulated, including eight cytochrome P450s, three carboxylesterase, and one glutathione peroxidase. Down-regulation of stress response gene expression suggested the existence of mechanism genes could potentially interfere with the proliferation of parasites in the midgut. Genes related to transport processes were differentially regulated in the *Plasmodium*-infected midgut transcriptome, with 38 repressed and 25 induced. A gene encoding oxidoreductase (AAEL003312) was significantly up-regulated, suggesting *Plasmodium*-mediated elevated activity of this enzyme (Table S6). Among potential functions of oxidoreductase is detoxification of reactive oxygen species (ROS), which play a pivotal role in anti-*Plasmodium* gut resistance [54].

A pronounced immune response was detected in the fat body of mosquitoes 24 h after a *Plasmodium*-infected blood meal (Figure 3 and Table S7). Significantly, *Plasmodium* infection resulted in the enrichment of 74 immune gene transcripts in the fat body (out of 99 regulated immune genes in this tissue). REL1 (AAEL007696) and REL2 (AAEL007624) were induced in the fat body transcriptome of the Plasmodium-infected mosquitoes, suggesting that infection with the parasite activated both the Toll and IMD pathways (Table S7). Components of the Toll pathway - *GNBP3,* TOLL8, TOLL11, and *spätzle 6 -* were also upregulated. The IMD receptors PGRP-LP and PGRP-S5 were elevated. However, *IMD* was down-regulated. Attacin C, Defensins C and D were among up-regulated AMPs.

A distinct immune response of the *Aedes* fat body to *Plasmodium* infection was the activation of two Down-syndrome adhesion molecules (Dscam, Table S7). Dscam is a member of the immunoglobulin superfamily, its gene comprises of multiple exons, alternative splicing of which generates 19,000 different extracellular domains and provides [55]. *An. gambiae* orthologue of Dscam contains 101 exons that can produce over 31,000 alternative splice forms [56]. Hemocyte-specific Dscam isoforms have been associated with phagocytotic uptake of bacteria [55], [57]. In *An. gambiae*, Dscam has been implicated in resistance to bacteria and Plasmodium [55]. *Dscam* is also expressed in Drosophila fat body, which is in agreement with our observation [55]. The role of fat body-specific Dscam isoforms remains to be elucidated.

Multiple *TEPs* (*TEP13*, *TEP15*, *TEP20*, *TEP22*, and *TEP23*) and leucine-rich (LRR) proteins were up-regulated in the fat body in response to *Plasmodium* infection as well; however, their functions are not clear. *Plasmodium* infection also caused changes in mRNA abundance in apoptosis related genes; IAP-2 (an inhibitor of apoptosis), CASPS18, and CASPS8 were induced, while CASPS19 was repressed in the fat body of the *Plasmodium*-infected mosquitoes. An interesting feature of *Plasmodium*-affected fat body transcriptome is the down-regulation of DOME, the JAK/STAT receptor, which was up-regulated in the REL1+ fat body transcriptome (Table S7). Another distinct feature of *Plasmodium*-affected fat body transcriptome was elevation transcriptional activity as evident by up-regulation of six zinc finger and forkhead transcription factors (Table S7).

We found that the fat body transcriptome, the gene encoding dual oxidase (DUOX, AAEL007563) was activated by *Plasmodium* infection (Table S7). DUOX enzyme is involved in production of reactive oxygen species (ROS), which have been implicated in anti-microbial immunity [58]. ROS has been implicated in innate immune responses in the gut and anti-Plasmodium defenses [54], [58], [59]. The anti-Plasmodium effect of ROS is mediated by bacterial flora [60]. The adverse effect of ROS is modulated by antioxidants, including Gpx [54]. Our finding of DUOX in the *Plasmodium*-induced fat body transcriptome adds a new aspect in immune function of ROS. A possibility of activation of these enzymes in hemocytes attached to the fat body could not be ruled out.

**Figure 4:**
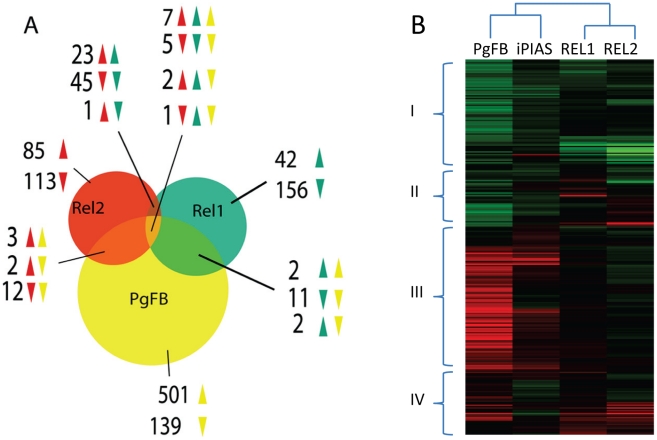
Comparative analysis of the *P. gallinaceum* fat body responsive to the *REL1*- and *REL2*-regulated transcriptomes. **A**) Venn diagram representing unique and shared gene transcript regulation in PgFB, REL1+, and REL2+ transgenic mosquitoes. The overlapping regions represent genes that are concomitantly regulated in two or three experimental conditions. The direction of gene regulation is represented by upward-pointing and downward-pointing arrows. **B**) Hierarchical cluster analysis of the genes that were regulated in REL1+, REL2+, *PIAS*-depleted, and *P. gallinaceum* challenged mosquitoes.

Availability of REL1+- and REL2+-induced fat body transcriptomes permitted us to conduct a comparative analysis with the *P. gallinaceum* fat body (PgFB)-responsive transcriptome. The comparison of fat body of *Plasmodium* infection-responsive gene transcript repertoire with those of REL1+ and REL2+ mosquitoes showed an overlap between these transcriptomes (7 induced, 5 repressed) (Figure 4 and Table S8). Only three immune genes (CLIPB13B, CLIPB15, and Serpin-8) were found in the overlap of these transcriptomes, suggesting that immune responses elicited by the *Plasmodium* infection in the fat body were distinct from those regulated by either REL1+- and REL2+ (Figure 4, Figure S2 and Table S8). Hierarchical clustering showed that cluster I consisted of a large group of down-regulated genes from PgFB-responsive and REL1+ and REL2+ gene repertoire, while cluster II represented genes, which were induced in by REL and repressed by *Plasmodium* challenge. Conversely, cluster IV was largely enriched by up-regulated immune genes (82%), which are putatively involved in melanization and signaling amplification (Serpins, CLIPs) and parasite recognition and killing (TEPs, Lectins) (Table S8).

### The JAK-STAT pathway is involved in mosquito anti-*Plasmodium* defense

The core components of the JAK-STAT pathway are evolutionarily conserved from arthropods to mammals [61]. PIAS (Protein Inhibitor of Activated STAT) has been identified as a negative regulator of the JAK-STAT pathway in mammals, *Drosophila* and *Aedes* [62], [63]. A multiple polypeptide sequence alignment showed that insect PIAS had a domain structure similar to that from vertebrates (Figures S4A and S4B). The sequence of *Aedes* PIAS shared a high level of homology with *Anopheles* PIAS (identity 61%, similarity 71%) and *Drosophila* PIAS (identity 47%, similarity 57%). It also had a lower degree of homology with human PIAS (identity 32%, similarity 46%); but the invertebrate PIAS group formed its own independent clade (Figure S4C). Impressively, *Ae aegypti PIAS* gene contains ten alternative spliced isoforms, however, their respective roles are not known (Figure S4) [3]. We compared the *Plasmodium* infection-responsive fat body transcriptome with that of *PIAS*-gene silenced mosquitoes, which was reported earlier [63] and found a significant overlap between these transcriptomes [63] (29 gene transcripts: 26 induced and 3 repressed) (Figure 3). The transcript of the *SOCS* (suppressor of cytokine signaling) gene, which encodes another JAK-STAT pathway negative regulator, was highly enriched in the fat body in response to *P. gallinaceum* infection. Interestingly, not a single gene displaying transcript enrichment upon *P. gallinaceum* infection seemed to be co-regulated by *cactus* and *PIAS* silencing, suggesting that anti-*Plasmodium* responses mediated by Toll and JAK-STAT pathways were different. Hierarchical cluster analysis revealed several genes with mRNA abundance enriched in both PgFB, and PIAS-depleted fat body transcriptomes, but unaffected in REL1+ and REL2+ fat body transcriptomes (Figure 4, Cluster III; and Table S8). As stated, cluster IV is mainly composed by immune genes, which are involved in melanization, pattern recognition, and signaling amplification (Table S8). Additionally, overlap between *PIAS*-depleted and PgFB transcriptome revealed numerous important immune genes: a putative LRR, whose gene family has been linked to *Plasmodium* killing in *An. gambiae* [33], [64] , *spätzle 6*, and two dengue virus restriction factors (*DVRF1* and *DVRF2*) [63] (Figure S2B).

**Figure 5:**
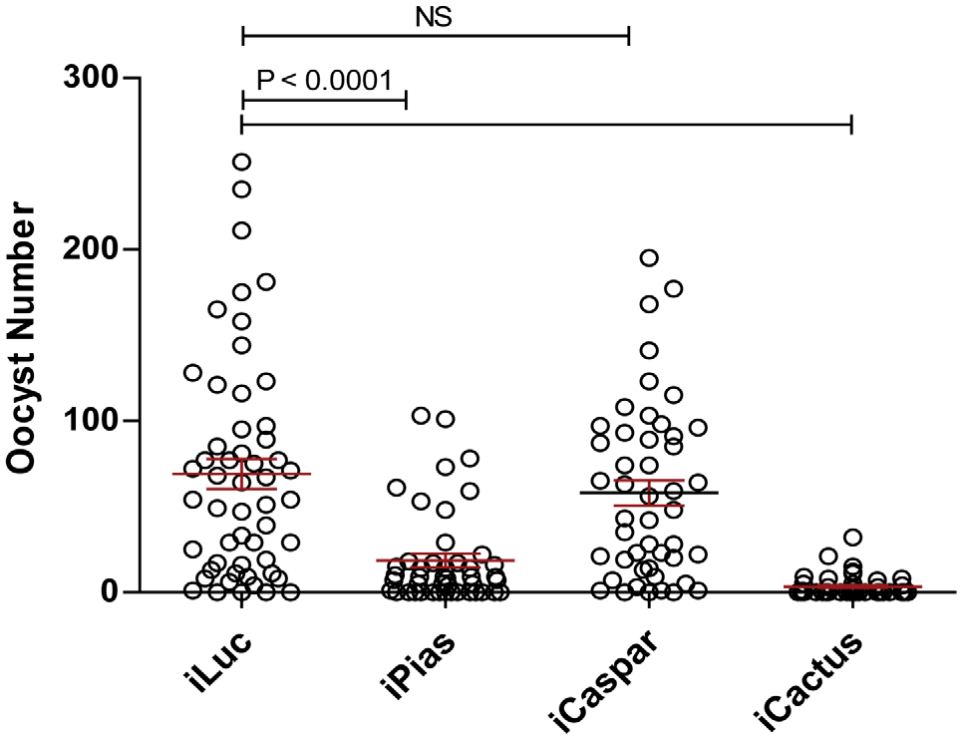
The role of PIAS in the defense against avian malaria parasite. The effect of cactus, caspar, and PIAS in the defense against avian malaria parasite was compared. Depletion of *PIAS* and *cactus* significantly decreased the number of survival oocysts. However, the RNAi knock-down of *caspar* had no effect in the infection of avian malaria parasite to mosquito *Ae. aegypti*. Prefix i indicates the RNAi-mediated depletion of certain genes by direct injection of their corresponding dsRNAs. iLuc (luciferase dsRNA) was used as controls. The number of fully developed oocysts in each midgut was shown as a circle. The mean number of parasite oocysts for each group was indicated by a black bar.

We then evaluated the anti-*P. gallinaceum* activities of three major *Ae aegypti immune pathways* Toll, IMD, and JAK-STAT. One-day-old mosquitoes were injected with dsRNA for either one of the following negative regulator genes of these pathway: *PIAS, caspar, cactus*, or *luc*, as a control (Figure S5), and then fed on *Plasmodium*-infected blood 4 days later. Depletion of *cactus*, and hence activation of the Toll pathway REL1, resulted in the highest level of resistance to *P. gallinaceum* (Figure 5). Knockdown of *PIAS*, which resulted in the activation of the JAK-STAT pathway-regulated immune response, also increased mosquito resistance to parasite infection in the midgut by a six-fold (Figure 5). However, we observed no anti-*P. gallinaceum* effect upon activation of the IMD pathway REL2 factor through depletion of *caspar* (Figure 5). Depletions of the negative regulators of Toll, IMD, and JAK-STAT pathways – *cactus*, *caspar* and *PIAS* – demonstrated differential patterns of resistance in different mosquito-*Plasmodium* infection models. REL1 activation by depletion of *cactus* resulted in the strongest anti– *P. berghei* and anti-*P. gallinaceum* effects in *An. gambiae* and *Ae. aegypti*, respectively [7], [10], [11], [15], [41]. Depletion of *caspar* has shown that the IMD pathway is most effective against the human pathogen *P. falciparum* in *An. gambiae* and other anopheline species [44]. In the present study we have not observed any effect of *caspar* depletion on the resistance of *Ae. aegypti* to *P. gallinaceum*, while our previous study based on overexpression of REL2 in transgenic *Ae. aegypti,* has clearly shown involvement of IMD pathway in defense against this pathogen [14]. This discrepancy, taken together with the findings from our transcriptome studies of *REL2+* and *caspar*-depleted mosquitoes, may suggest that caspar is likely to regulate a branch of the IMD pathway, involving a subset of effector genes. Moreover, we have also demonstrated that simultaneous overexpression of two anti-microbial peptides, Cecropin A and Defensin A, which are under the dual control of Toll and IMD pathways, lead to a complete elimination of *P. gallinaceum* and termination of transmission [51]. In this current study, we have implicated the JAK-STAT pathway in anti-*Plasmodium* defense in *Ae. aegypti*. The STAT pathway is involved in late-phase immunity against *P. berghei and P. falciparum* in *An. gambiae* [65].

In conclusion, utilization of transgenic *Ae. aegypti* mosquitoes with altered immunity by means of ectopic expression of the NF-kB transcription factors REL1 and REL2 has permitted deciphering gene repertoires activated by Toll and IMD pathways in the fat body, the central tissue to mosquito immunity. Importantly, transgenic mosquitoes ectopically expressing both these factors exhibited strong synergistic activation of immune genes. A close correlation has been noted between REL1+ and *cactus*-depleted transcriptomes. In contrast, the REL2+ transcriptome was strikingly different from that of *caspar*-depleted mosquitoes, suggesting that caspar regulates a sub branch of the IMD pathway. Infections of the wild type *Ae. aegypti* with *P. gallinaceum* elicited enrichment of a distinct subset (76 up- and 25 down regulated) of immune gene transcripts relative to that observed in REL1+, REL2+, and cactus-depleted mosquitoes. Considerable overlap was observed between the fat body transcriptome of *Plasmodium*-infected mosquitoes and that of mosquitoes depleted of *PIAS*, the inhibitor of the JAK-STAT pathway. PIAS gene silencing reduced *Plasmodium* proliferation in *Ae. aegypti*, indicating the involvement of the JAK-STAT pathway in anti-*Plasmodium* defense in this infection model in addition to Toll and IMD pathways.

## Materials and Methods

### Ethics statement

This study was carried out in strict accordance with the recommendations in the Guide for the Care and Use of Laboratory Animals of the National Institutes of Health. The protocol was approved by the University of California Riverside Institutional Animal Care and Use Committee (IACUC #A20100016; 05/27/2010) and all efforts were made to minimize suffering.

### Experimental animals

Wild type and transgenic *Ae. aegypti* mosquitoes, of the UGAL/Rockefeller strain, REL1+ [13] and REL2+ [14], were maintained in laboratory culture under conditions of 27°C and 80% humidity. Female mosquitoes 3–5 days post-eclosion were fed on the blood of anesthetized white rats to initiate egg development.

Hybrid REL1+/REL2+ mosquitoes were generated by crossing REL1+ and REL2+. Selection of hybrids was performed as previously described [51]. To generate these hybrids, the two strains, REL1+ and REL2+, were maintained as homozygous for four generations before crossing. The hybrid strain was established by crossing REL1 females with REL2 males; the F1 hybrid females were used for the experiments. Adult mosquitoes were maintained on 10% sucrose solution and water [66]. The avian malaria *P. gallinaceum* was maintained under the natural transmission cycle between the mosquito and chickens. To determine the number of parasite oocysts in the midgut dissected at 8 days post-infection, the tissue was stained with 1% mercurochrome and oocysts were counted under Nikon E400 light microscopy. All dissections (fat body and midgut) were performed in *Aedes* physiological solution (APS) [66]. Abdominal walls with adhering fat body tissue and free from other internal tissues (thereafter called fat body) were washed in APS to removed hemolymph and blood cells before freezing in liquid nitrogen. Midgut preparations included the anterior and posterior (stomach) without Malpighian tubules and hindgut.

### Computational analysis

PIAS sequences from different metazoan species, retrieved from NCBI, Vectorbase, and Ensembl, were analyzed in PROSITE and SMART to confirm conserved domain structures. Multiple sequences were aligned in ClustlX2.0 (Blosum matrixes, gap penalty 10, and extension penalty 0.1). A phylogenetic tree was constructed based on the neighbor-joining method and displayed by Treeview. Parasite oocyst data generated from three independent experiments were analyzed using the Kolmogorov-Smirnov goodness-of-fit test and pooled. The statistically significant difference between samples was calculated using the Mann-Whitney test (Graphpad 5.0).

### RT-PCR, Real-time PCR, and Northern blot analysis

Total RNA was extracted from the fat body of eight mosquitoes using the Trizol method (Invitrogen), according to the manufacturer's protocol. Total RNA (5 µg) from each sample was separated on a formaldehyde gel, blotted and hybridized with the corresponding ^32^P-labeled DNA probe. Probes were generated using PCR and then following the High Prime (Roche) protocol. Actin was used as a loading control. For RT-PCR and Real-time PCR, cDNAs were synthesized from 2 µg total RNA using Omniscript Reverse Transcriptase kit (Qiagen). RNA was treated with DNase I (Invitrogen) before cDNA synthesis. PCR was performed using the Platinum High Fidelity Supermix (Invitrogen). Real-time PCR was performed on the iCycler iQ system (Bio-Rad, Hercules, CA) and we used an IQ SYBR green supermix (Bio-Rad). Quantitative measurements were performed in triplicate and normalized to the internal control of S7 ribosomal protein mRNA for each sample. Primers and probes are listed in Table S9. Real-time data were collected from the software iCycler v3.0. Raw data were exported to EXCEL for analysis.

### Gene expression knockdown

Double-stranded RNA synthesis followed a method described previously [10], [15]. In brief, double-stranded RNA (dsRNA) of specific gene template was synthesized using the MEGAscript kit (Ambion). The luciferase gene was used to generate control iLuc dsRNA. After dsRNA synthesis, samples were treated by means of phenol/chloroform extraction and then ethanol precipitation. DsRNA was then suspended in Rnase-free water to reach a final concentration of 5 µg/µl. Naïve adult female mosquitoes were selected at 24 h post-emergence for dsRNA injection experiments. The Picospritzer II (General Valve, Fairfield, NJ) was used to introduce corresponding dsRNA into the thorax of CO_2_-anesthetized mosquito females, at one or two days post-emergence. DsRNA (300 nl) was injected into the thorax of each adult *Ae. aegypti* female mosquito. Primers used for dsRNA knockdowns are listed in Table S9.

### Microarray assays

Transcription assays and analysis were conducted following standard protocols with a full genome Agilent-based microarray platform [15]. Relative mRNA abundance was compared between treated and control samples. In brief, 2–3 µg total RNA was used for probe synthesis of cy3- and cy5-labeled dCTP. Hybridizations were conducted with an Agilent Technologies *In Situ* Hybridization kit at 60°C, according to the manufacturer's instructions. Hybridization intensities were determined with an Axon GenePix 4200AL scanner, and images were analyzed with Gene Pix software. The expression data were processed and analyzed as described previously [15]. In brief, the background-subtracted median fluorescent values were normalized according to a LOWESS normalization method, and Cy5/Cy3 ratios from replicate assays were subjected to *t*-tests at a significance level of p<0.05, using TIGR, MIDAS, and MeV software [67]. Expression data from all replicate assays were averaged with the GEPAS microarray preprocessing software prior to logarithm (base 2) transformation. Self–self hybridizations were used to determine the cut-off value for the significance of gene regulation on these types of microarrays to 0.8 in log2 scale, which corresponds to 1.74-fold regulation [68]. For genes with p<0.01, the average ratio was used as the final fold change; for genes with p>0.01, the inconsistent replicates (with distance to the median of replicate ratios larger than 0.8) were removed, and only the value from a gene with at least two replicates in the same direction of regulation were further averaged. Three independent biological replicate assays were performed. Numeric microarray gene expression data are presented in Tables S1, S2, S5, S6, S7; validation data by quantitative Real-time PCR- in Table S10 and Figure S3.

## Supporting Information

Figure S1 **Blood meal activated expression of REL2 in transgenic** ***Ae. aegypti*** **mosquitoes.** Transgenic mosquitoes with ectopic expression of *REL2* under the control of the fat body-specific *Vg* promoter were fed blood and total RNA was isolated from fat bodies at time points of 0, 12, 24, 36 h post blood meal (PBM). Samples were analyzed for REL2 transcript abundance by means of quantitative RT-PCR. Data were presented in fold induction relative to S7. Data (means ± standard errors of the means) from three independent experiments are shown.
(PDF)

Figure S2 **Comparative transcriptome analysis of immune genes from fat bodies of** ***Ae. aegypti*** **female mosquitoes after ectopic expression of REL1, REL2 or after Plasmodium infection.** **A)** Venn diagram of melanization gene regulation in REL1+, REL2+, and *Plasmodium*-infected (PgFB) mosquitoes. The overlapping regions represent genes that are concomitantly regulated in two, three experimental conditions at the level of transcript abundance. The direction of gene transcript changes is indicated by upward-pointing and downward-pointing arrows. Green, Yellow, and Dark blue colors represent REL1+, REL2+, and *Plasmodium*-infected mosquitoes, respectively. **B)** Venn diagram of immune gene regulation in *PIAS*-depleted and *Plasmodium*-infected (PgFB) mosquitoes. Blue and Red colors represent *Plasmodium*-infected and *PIAS*-depleted mosquitoes, respectively. **C)** Venn diagram of immune gene regulation in *cactus*-depleted and *Plasmodium*-infected (PgFB) mosquitoes. Blue and Purple colors represent *Plasmodium*-infected and *cactus*-depleted mosquitoes, respectively.
(PDF)

Figure S3 **Validation of microarray expression data by means of quantitative Real-time PCR.** The mean value of the expression data (log2 ratio) for 23 genes (12 from PgFB and 11 from PgMD) obtained by microarray analysis (Y axis) were plotted against the corresponding values obtained using quantitative Real-time RT-PCR (X-axis). The linear regression of data (goodness of fit: R^2^ = 0.77) presented a high degree of correlation between these two assays. The numeric values are presented in Table S10.
(PDF)

Figure S4 **Comparative analysis of the PIAS structure, the negative regulator of JAK-STAT signaling. A)** *Aedes* PIAS shares the same domain structure as those from other insects and mammals. It contains the SAP domain, PINIT motif, Ring finger Like Domain (RL), and also harbors a Serine/Threonine-rich domain in the C-terminal. **B)** PIAS sequences from four Dipteran species are aligned. SAP domain, RL domain, PINIT motif, and S/T rich region were indicated by close box with Red, Blue, Pink, and Green colors respectively. **C)** The constructed phylogenetic tree shows arthropod and vertebrate PIAS undergoing distinct evolutionary routes. Bootstrap values were indicated along with the node. Species name abbreviations: Ce, *Caenorhabditis elegans*; Dm, *Drosophila melanogaster*; Aa, *Aedes aegypti*; Hs, *Homo sapiens*; Tc, *Tribolium castaneum*; Gg, *Gallus gallus*; Ap, *Apis mellifera*; Is, *Ixode scapularis*; Nv, *Nasonia vitripennis*; Bm, *Bombyx mori*; Xt, *Xenopus tropicalis*.
(PDF)

Figure S5 **Transcriptional knockdowns of** ***cactus*** **,** ***caspar*** **, and** ***PIAS*** **confirmed by Northern analysis.** Depletion of PIAS induced expression level of SOCS36E, a JAK-STAT pathway reporter gene. Knock-down of *cactus* induced expression of Toll pathway specific gene CLIPB29, Clip domain serine protease. Depletion of *PIAS* caused the transcription level of JAK-STAT pathway specific gene to increase. However, depletion of *caspar* did not induce the *defensin* gene. Defensin, SOCS36E, and PPO1 were induced by bacteria and fungi challenge. CLIPB29 was induced only by fungi challenge. Action was used as a loading control. The transcriptional knockdown of *cactus*, *caspar*, and *PIAS* were confirmed by means of Northern analysis. Naïve, Naïve UGAL; 5 h EC, 5 hr after *E. cloacae* challenge; 2D BB, 2 days after *B. bassiana* challenge. Septic injuries were performed by pricking female adult mosquitoes in the rear part of the abdomen with an acupuncture needle dipped into either *Enterobacter cloacae* bacterial culture or a fungal spore suspension of *Beauveria bassiana* strain GHA.
(PDF)

Table S1 **Repertoire of genes affected by ectopic expression of REL1 in the fat body of the transgenic** ***Ae. aegypti*** **female mosquitoes.** Data obtained by means of a full genome Agilent-based microarray analysis. Gene ID, gene name, functional group and log fold increase (decrease) are presented. Abbreviations for functional groups: IMM, immunity; R/S/M, redox, stress and mitochondrion; DIG, digestive; C/S, cytoskeletal and structural; PROT, proteolysis; TRP, transport; R/T/T, replication, transcription, and translation; MET, metabolism; DIV, diverse functions; UNK, unknown functions.
(DOCX)

Table S2 **Repertoire of genes affected by ectopic expression of REL2 in the fat body of the transgenic** ***Ae. aegypti*** **female mosquitoes.** Data obtained by means of a full genome Agilent-based microarray analysis. Gene ID, gene name, functional group and log fold increase (decrease) are presented. Abbreviations for functional groups: IMM, immunity; R/S/M, redox, stress and mitochondrion; DIG, digestive; C/S, cytoskeletal and structural; PROT, proteolysis; TRP, transport; R/T/T, replication, transcription, and translation; MET, metabolism; DIV, diverse functions; UNK, unknown functions.
(DOCX)

Table S3 **Hierarchical clustering of genes regulated by fat body-specific ectopic expression of** ***REL1*** **and** ***REL2*** **, RNAi depletions of** ***Cactus*** **(CAC) and** ***Caspar*** **(CASP) in** ***Ae. aegypti*** **females.** Abbreviations for functional groups: IMM, immunity; R/S/M, redox, stress and mitochondrion; DIG, digestive; C/S, cytoskeletal and structural; PROT, proteolysis; TRP, transport; R/T/T, replication, transcription, and translation; MET, metabolism; DIV, diverse functions; UNK, unknown functions.
(DOCX)

Table S4 **Repertoire of genes found in both REL1- and REL2-affected fat body transcriptomes in transgenic** ***Ae. aegypti*** **female mosquitoes.** Gene ID, gene name, functional group and log fold increase (decrease) are presented. IMM, immunity; R/S/M, redox, stress and mitochondrion; C/S, cytoskeletal and structural; PROT, proteolysis; TRP, transport; R/T/T, replication, transcription, and translation; MET, metabolism; DIV, diverse functions; UNK, unknown functions.
(DOCX)

Table S5 **Repertoires of genes putatively related to the melanization pathway in** ***Ae. aegypti*** **female mosquitoes.** Data from transcriptome analyses after - REL1-fat body specific ectopic expression (REL1); REL2-fat body specific ectopic expression (REL2), *Plasmodium gallinaceum*-infected mosquito midgut (Pg midgut); *Pl. gallinaceum*-infected mosquito fat body (Pg FB); RNAi depletion of PIAS; RNAi depletion of cactus (CAC); RNAi depletion of caspar (CASP).
(DOCX)

Table S6 **Gene repertoire induced in the** ***Aedes aegypti*** **midgut, 24 h after infection with** ***P. gallinaceum*** **.** Data obtained by means of a full genome Agilent-based microarray analysis. Gene ID, gene name, functional group and log fold increase (decrease) are presented. Abbreviations are: IMM, immunity; R/S/M, redox, stress and mitochondrion; DIG, blood and sugar food digestive; C/S, cytoskeletal and structural; PROT, proteolysis; TRP, transport; R/T/T, replication, transcription, and translation; MET, metabolism; DIV, diverse functions; UNK, unknown functions.
(DOCX)

Table S7 **Gene repertoire induced in the** ***Aedes aegypti*** **fat body, 24 h after infection with** ***P. gallinaceum*** **.** Data obtained by means of a full genome Agilent-based microarray analysis. Gene ID, gene name, functional group and log fold increase (decrease) are presented. Abbreviations are: IMM, immunity; R/S/M, redox, stress and mitochondrion; DIG, blood and sugar food digestive; C/S, cytoskeletal and structural; PROT, proteolysis; TRP, transport; R/T/T, replication, transcription, and translation; MET, metabolism; DIV, diverse functions; UNK, unknown functions.
(DOCX)

Table S8 **Hierarchical clustering of genes regulated by** ***P. gallinaceum*** **in the fat bodies (PgFB), RNAi PIAS depletion, REL1 and REL2 fat body-specific ectopic expression.** Abbreviations are: IMM, immunity; R/S/M, redox, stress and mitochondrion;; DIG, digestive; C/S, cytoskeletal and structural; PROT, proteolysis; TRP, transport; R/T/T, replication, transcription, and translation; MET, metabolism; DIV, diverse functions; UNK, unknown functions.
(DOCX)

Table S9 **Primers used for gene expression knockdowns and for quantitative Real-Time PCR.**
(DOCX)

Table S10 **Validation of microarray expression data by means of quantitative Real-time PCR.** PgFB – Plasmodium affected fat body transcriptome; PgMD - Plasmodium affected midgut transcriptome.
(DOCX)
