# Supplementary figures and images for: Transcriptome Analysis of Aedes aegypti Transgenic Mosquitoes with Altered Immunity
Source: PLoS Pathog. 2011 Nov 17;7(11):e1002394. doi: 10.1371/journal.ppat.1002394 (PMC3219725; doi:10.1371/journal.ppat.1002394)

## REL2

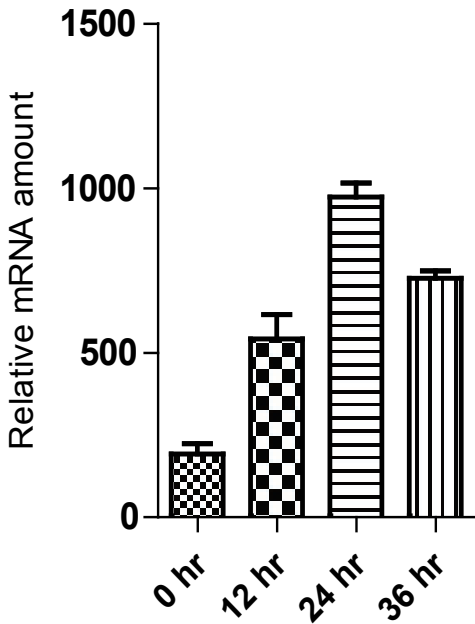

Supplement: Figure S1 — Blood meal activated expression of REL2 in transgenic Ae. aegypti mosquitoes. Transgenic mosquitoes with ectopic expression of REL2 under the control of the fat body-specific Vg promoter were fed blood and total RNA was isolated from fat bodies at time points of 0, 12, 24, 36 h post blood meal (PBM). Samples were analyzed for REL2 transcript abundance by means of quantitative RT-PCR. Data were presented in fold induction relative to S7. Data (means ± standard errors of the means) from three independent experiments are shown. (PDF) [file ppat.1002394.s001.pdf]

**A**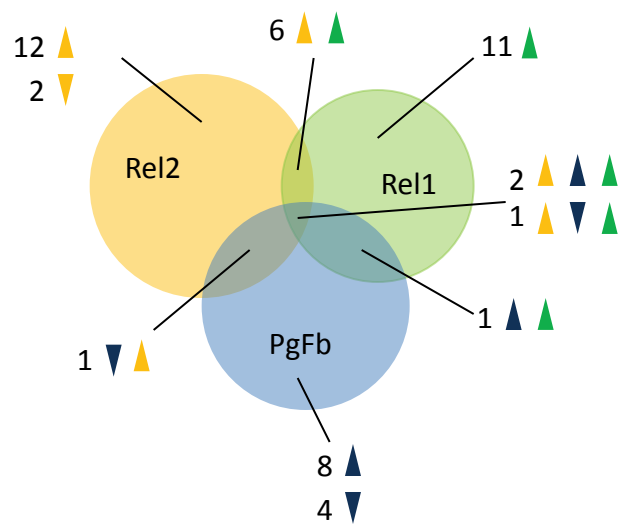**B**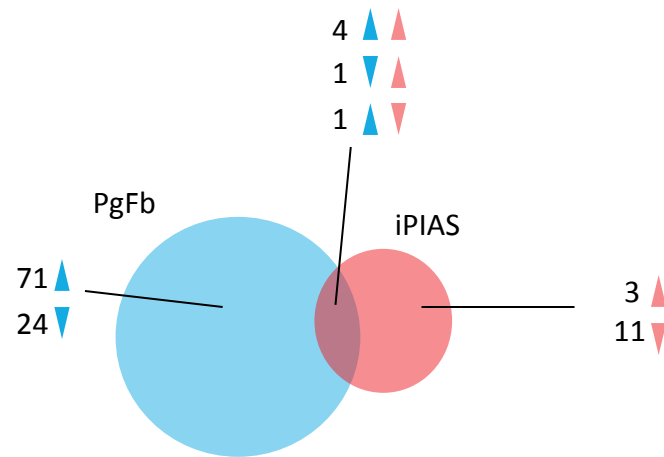**C**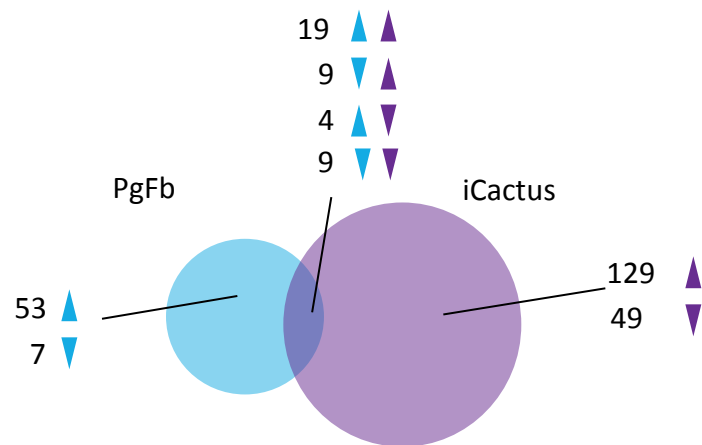

Supplement: Figure S2 — Comparative transcriptome analysis of immune genes from fat bodies of Ae. aegypti female mosquitoes after ectopic expression of REL1, REL2 or after Plasmodium infection. A) Venn diagram of melanization gene regulation in REL1+, REL2+, and Plasmodium-infected (PgFB) mosquitoes. The overlapping regions represent genes that are concomitantly regulated in two, three experimental conditions at the level of transcript abundance. The direction of gene transcript changes is indicated by upward-pointing and downward-pointing arrows. Green, Yellow, and Dark blue colors represent REL1+, REL2+, and Plasmodium-infected mosquitoes, respectively. B) Venn diagram of immune gene regulation in PIAS-depleted and Plasmodium-infected (PgFB) mosquitoes. Blue and Red colors represent Plasmodium-infected and PIAS-depleted mosquitoes, respectively. C) Venn diagram of immune gene regulation in cactus-depleted and Plasmodium-infected (PgFB) mosquitoes. Blue and Purple colors represent Plasmodium-infected and cactus-depleted mosquitoes, respectively. (PDF) [file ppat.1002394.s002.pdf]

$$R^2=0.77$$

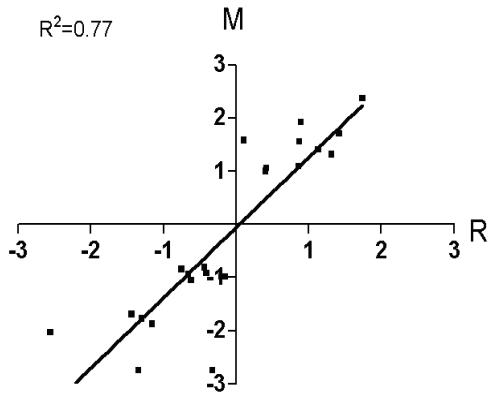

Supplement: Figure S3 — Validation of microarray expression data by means of quantitative Real-time PCR. The mean value of the expression data (log2 ratio) for 23 genes (12 from PgFB and 11 from PgMD) obtained by microarray analysis (Y axis) were plotted against the corresponding values obtained using quantitative Real-time RT-PCR (X-axis). The linear regression of data (goodness of fit: R2 = 0.77) presented a high degree of correlation between these two assays. The numeric values are presented in Table S10. (PDF) [file ppat.1002394.s003.pdf]

Naive  
5h EC  
2D BB  
iLuc  
iCactus  
iCaspar  
iPIAS

B29

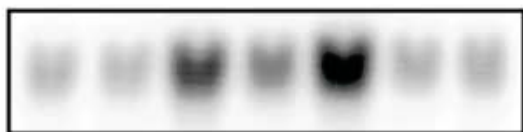

PPO1

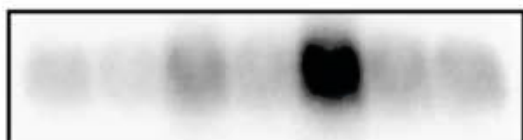

DefA

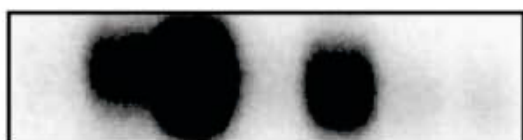

SOCS36E

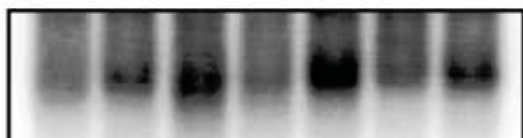

Actin

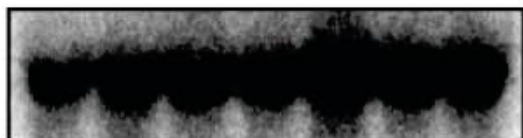

Cactus

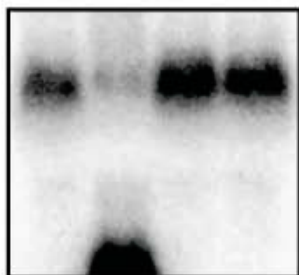

Caspar

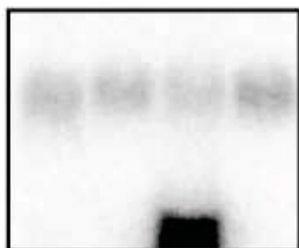

PIAS

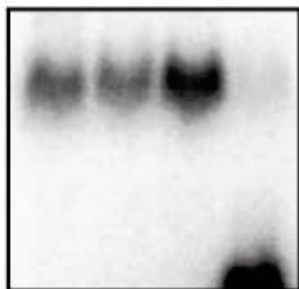

Supplement: Figure S5 — Transcriptional knockdowns of cactus , caspar , and PIAS confirmed by Northern analysis. Depletion of PIAS induced expression level of SOCS36E, a JAK-STAT pathway reporter gene. Knock-down of cactus induced expression of Toll pathway specific gene CLIPB29, Clip domain serine protease. Depletion of PIAS caused the transcription level of JAK-STAT pathway specific gene to increase. However, depletion of caspar did not induce the defensin gene. Defensin, SOCS36E, and PPO1 were induced by bacteria and fungi challenge. CLIPB29 was induced only by fungi challenge. Action was used as a loading control. The transcriptional knockdown of cactus, caspar, and PIAS were confirmed by means of Northern analysis. Naïve, Naïve UGAL; 5 h EC, 5 hr after E. cloacae challenge; 2D BB, 2 days after B. bassiana challenge. Septic injuries were performed by pricking female adult mosquitoes in the rear part of the abdomen with an acupuncture needle dipped into either Enterobacter cloacae bacterial culture or a fungal spore suspension of Beauveria bassiana strain GHA. (PDF) [file ppat.1002394.s005.pdf]
