# Supplementary material for: Transcriptome Analysis of Aedes aegypti Transgenic Mosquitoes with Altered Immunity
Source: PLoS Pathog. 2011 Nov 17;7(11):e1002394. doi: 10.1371/journal.ppat.1002394 (PMC3219725; doi:10.1371/journal.ppat.1002394)
Supplement: Figure S4 — Comparative analysis of the PIAS structure, the negative regulator of JAK-STAT signaling. A) Aedes PIAS shares the same domain structure as those from other insects and mammals. It contains the SAP domain, PINIT motif, Ring finger Like Domain (RL), and also harbors a Serine/Threonine-rich domain in the C-terminal. B) PIAS sequences from four Dipteran species are aligned. SAP domain, RL domain, PINIT motif, and S/T rich region were indicated by close box with Red, Blue, Pink, and Green colors respectively. C) The constructed phylogenetic tree shows arthropod and vertebrate PIAS undergoing distinct evolutionary routes. Bootstrap values were indicated along with the node. Species name abbreviations: Ce, Caenorhabditis elegans; Dm, Drosophila melanogaster; Aa, Aedes aegypti; Hs, Homo sapiens; Tc, Tribolium castaneum; Gg, Gallus gallus; Ap, Apis mellifera; Is, Ixode scapularis; Nv, Nasonia vitripennis; Bm, Bombyx mori; Xt, Xenopus tropicalis. (PDF) [file ppat.1002394.s004.pdf]

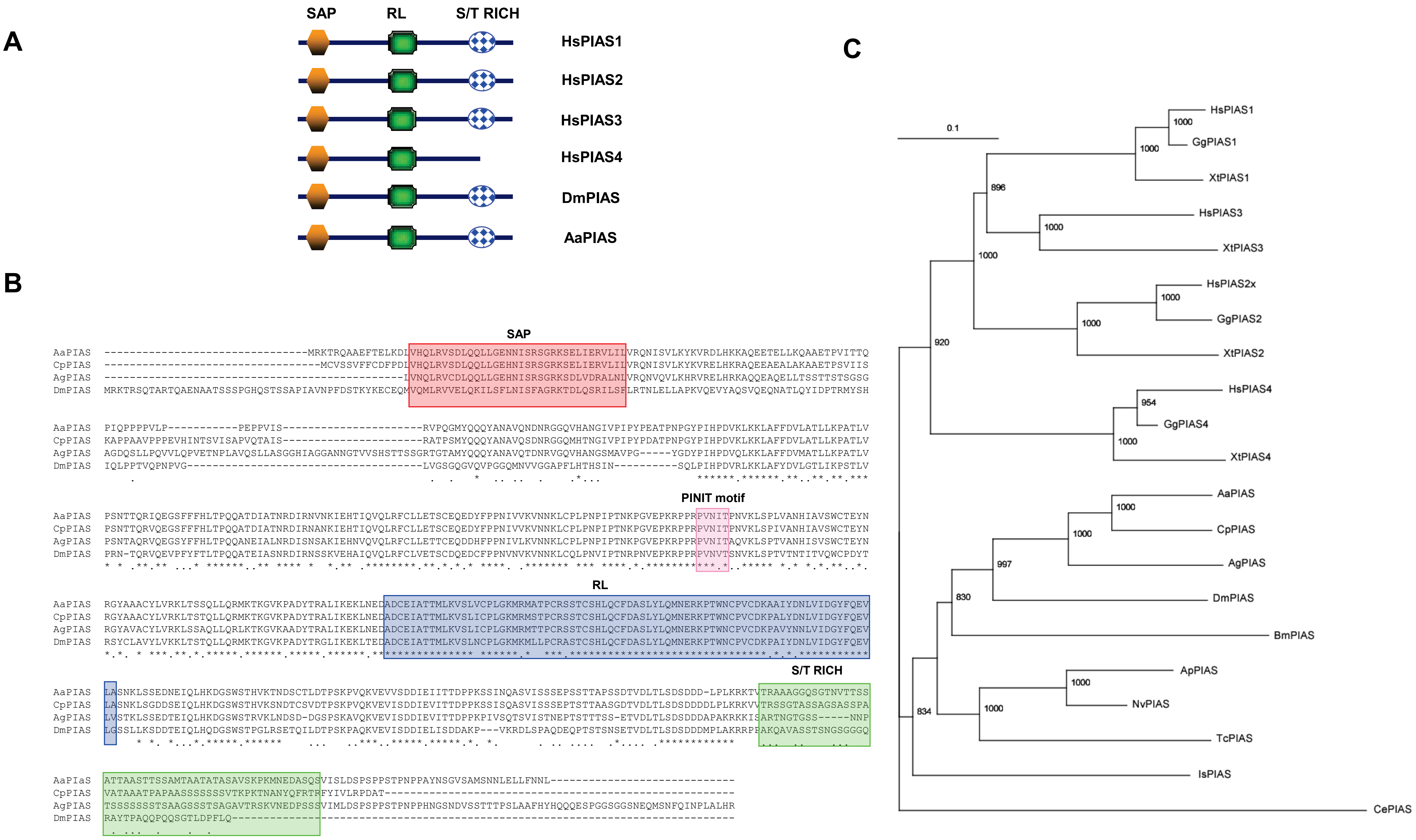
A
SAP
RL
S/T RICH
HsPIAS1
HsPIAS2
HsPIAS3
HsPIAS4
DmPIAS
AaPIAS
B
SAP
PINIT motif
RL
S/T RICH
AaPIAS
CpPIAS
AgPIAS
DmPIAS
C
0.1
HsPIAS1
GgPIAS1
XtPIAS1
HsPIAS3
XtPIAS3
HsPIAS2x
GgPIAS2
XtPIAS2
HsPIAS4
GgPIAS4
XtPIAS4
AaPIAS
CpPIAS
AgPIAS
DmPIAS
BmPIAS
ApPIAS
NvPIAS
TcPIAS
IsPIAS
CePIAS
1000
1000
896
1000
1000
1000
1000
920
954
1000
1000
1000
997
830
1000
1000
834

Fig. 4

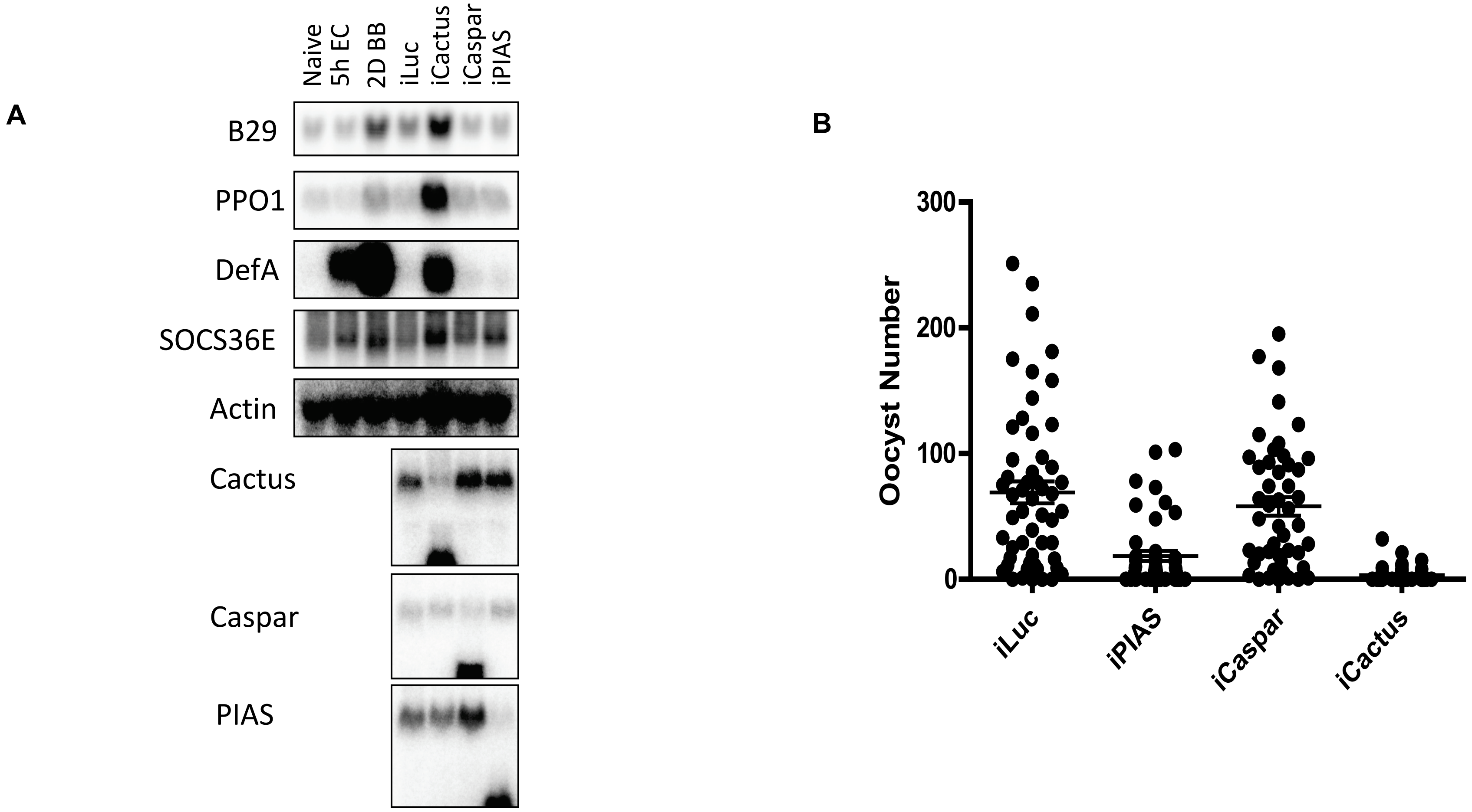
A
Naive
5h EC
2D BB
iLuc
iCactus
iCaspar
iPIAS
B29
PPO1
DefA
SOCS36E
Actin
Cactus
Caspar
PIAS
B
Oocyst Number
300
200
100
0
iLuc
iPIAS
iCaspar
iCactus
