# Supplementary material for: Transcriptome Analysis of Aedes aegypti Transgenic Mosquitoes with Altered Immunity
Source: PLoS Pathog. 2011 Nov 17;7(11):e1002394. doi: 10.1371/journal.ppat.1002394 (PMC3219725; doi:10.1371/journal.ppat.1002394)
Supplement: Table S1 — Repertoire of genes affected by ectopic expression of REL1 in the fat body of the transgenic Ae. aegypti female mosquitoes. Data obtained by means of a full genome Agilent-based microarray analysis. Gene ID, gene name, functional group and log fold increase (decrease) are presented. Abbreviations for functional groups: IMM, immunity; R/S/M, redox, stress and mitochondrion; DIG, digestive; C/S, cytoskeletal and structural; PROT, proteolysis; TRP, transport; R/T/T, replication, transcription, and translation; MET, metabolism; DIV, diverse functions; UNK, unknown functions. (DOCX) [file ppat.1002394.s006.docx]

Table S1. Repertoire of genes affected by ectopic expression of REL1 in the fat body of the transgenic *Ae. aegypti* female mosquitoes. Data obtained by means of a full genome Agilent-based microarray analysis. Gene ID, gene name, functional group and log fold increase (decrease) are presented. Abbreviations for functional groups: IMM, immunity; R/S/M, redox, stress and mitochondrion; DIG, digestive; C/S, cytoskeletal and structural; PROT, proteolysis; TRP, transport; R/T/T, replication, transcription, and translation; MET, metabolism; DIV, diverse functions; UNK, unknown functions.

| GENE ID | Name | Func group | Logfold |
| --- | --- | --- | --- |
| AAEL001392 | hypothetical protein | UNK | 1.99 |
| AAEL012471 | DOME | IMM | 1.98 |
| AAEL008207 | serine protease | IMM | 1.85 |
| AAEL005253 | abc transporter | DIV | 1.35 |
| AAEL009842 | keratinocyte lectin, putative | IMM | 1.35 |
| AAEL003832 | DEFC | IMM | 1.34 |
| AAEL007969 | serine protease | IMM | 1.33 |
| AAEL011453 | galactose-specific C-type lectin, putative | IMM | 1.29 |
| AAEL002702 | hypothetical protein | UNK | 1.22 |
| AAEL003841 | DEFA | IMM | 1.19 |
| AAEL007107 | serine protease, putative | IMM | 1.17 |
| AAEL008757 | juvenile hormone esterase | R/S/M | 1.17 |
| AAEL014139 | proacrosin, putative | MET | 1.16 |
| AAEL008208 | serine protease | IMM | 1.16 |
| AAEL002559 | Cu-Zn-superoxide dismutase precursor | DIV | 1.15 |
| AAEL011407 | type II transmembrane receptor OtB7, putative | IMM | 1.14 |
| AAEL002492 | Gasp-PA | DIV | 1.14 |
| AAEL003857 | DEFD | IMM | 1.13 |
| AAEL015527 | serine protease, putative | PROT | 1.11 |
| AAEL003723 | LYSC11 | IMM | 1.11 |
| AAEL002585 | serine protease | IMM | 1.1 |
| AAEL002255 | arylalkylamine N-acetyltransferase | DIV | 1.1 |
| AAEL000709 | CACTUS | IMM | 1.09 |
| AAEL006334 | sulfotransferase (sult) | DIV | 1.08 |
| AAEL012953 | elastase, putative | PROT | 1.08 |
| AAEL003632 | CLIPB39 | IMM | 1.06 |
| AAEL004667 | conserved hypothetical protein | UNK | 1.05 |
| AAEL011455 | galactose-specific C-type lectin, putative | IMM | 1.04 |
| AAEL000087 | TEP22 | IMM | 1.02 |
| AAEL012028 | proacrosin, putative | MET | 1.02 |
| AAEL003568 | threonine dehydratase/deaminase | DIV | 1.01 |
| AAEL001508 | conserved hypothetical protein | UNK | 0.99 |
| AAEL008364 | SRPN9 | IMM | 0.98 |
| AAEL000024 | dopachrome-conversion enzyme (DCE), putative | IMM | 0.96 |
| AAEL008607 | tep3 | IMM | 0.96 |
| AAEL013933 | SRPN4B | IMM | 0.96 |
| AAEL014141 | SRPN5 | IMM | 0.95 |
| AAEL003243 | serine protease | IMM | 0.94 |
| AAEL007696 | REL1 | IMM | 0.93 |
| AAEL001802 | TEP21 | IMM | 0.93 |
| AAEL014755 | tep2 | IMM | 0.93 |
| AAEL006137 | serpin-19 | IMM | 0.93 |
| AAEL002672 | matrix metalloproteinase | PROT | 0.92 |
| AAEL002624 | serine protease | IMM | 0.92 |
| AAEL011607 | galactose-specific C-type lectin, putative | IMM | 0.92 |
| AAEL014349 | serine protease | IMM | 0.92 |
| AAEL008596 | Sp?tzle 3A (Spz3A) | IMM | 0.92 |
| AAEL012956 | elastase, putative | IMM | 0.91 |
| AAEL005064 | serine protease | IMM | 0.91 |
| AAEL014079 | SRPN1 | IMM | 0.91 |
| AAEL002576 | sodium/solute symporter | TRP | 0.9 |
| AAEL012353 | antifreeze protein, putative | IMM | 0.9 |
| AAEL014382 | galactose-specific C-type lectin, putative | IMM | 0.9 |
| AAEL002301 | serine protease | IMM | 0.9 |
| AAEL008609 | zinc carboxypeptidase | PROT | 0.9 |
| AAEL012518 | Low density lipoprotein receptor adapter protein | DIV | 0.89 |
| AAEL011404 | galactose-specific C-type lectin, putative | IMM | 0.89 |
| AAEL002688 | glucosyl/glucuronosyl transferases | MET | 0.88 |
| AAEL013937 | SRPN4C | IMM | 0.87 |
| AAEL015458 | transferrin | IMM | 0.86 |
| AAEL014138 | SRPN16 | IMM | 0.85 |
| AAEL014950 | conserved hypothetical protein | UNK | 0.85 |
| AAEL000611 | CECE | IMM | 0.85 |
| AAEL001794 | TEP20 | IMM | 0.85 |
| AAEL003889 | GNBP1 | IMM | 0.85 |
| AAEL011777 | SRPN8 | IMM | 0.85 |
| AAEL002580 | predicted protein | IMM | 0.85 |
| AAEL008404 | Trypsin, putative | DIG | 0.85 |
| AAEL002601 | serine protease, putative | IMM | 0.84 |
| AAEL005641 | galactose-specific C-type lectin, putative | IMM | 0.84 |
| AAEL000059 | proacrosin, putative | MET | 0.84 |
| AAEL003253 | CLIPB13B | IMM | 0.83 |
| AAEL003816 | hypothetical protein | UNK | 0.83 |
| AAEL005670 | SRPN1 | IMM | 0.82 |
| AAEL003614 | CLIPB40 | IMM | 0.82 |
| AAEL002629 | serine protease | IMM | 0.81 |
| AAEL003616 | conserved hypothetical protein | UNK | 0.8 |
| AAEL009195 | Major facilitator superfamily domain-containing protein | DIV | 0.8 |
| AAEL014350 | serine protease, putative | PROT | 0.8 |
| AAEL010146 | 3-hydroxyacyl-coa dehyrogenase | MET | -0.8 |
| AAEL015524 | 3-hydroxyacyl-coa dehyrogenase | MET | -0.8 |
| AAEL002331 | mannose-6-phosphate isomerase | MET | -0.8 |
| AAEL013069 | receptor for activated protein kinase c (rack1) | DIV | -0.8 |
| AAEL000776 | Tripartite motif-containing protein 37 | DIV | -0.8 |
| AAEL003150 | alpha-n-acetylglucosaminidase | DIV | -0.8 |
| AAEL012733 | 60S ribosomal protein L19 | R/T/T | -0.8 |
| AAEL005749 | lysosomal alpha-mannosidase (mannosidase alpha class 2b member 1) | MET | -0.8 |
| AAEL012763 | metalloproteinase | DIV | -0.8 |
| AAEL003345 | argininosuccinate lyase | DIV | -0.8 |
| AAEL007692 | DNA polymerase subunit alpha B | R/T/T | -0.8 |
| AAEL006081 | nadph fad oxidoreductase | R/S/M | -0.81 |
| AAEL010168 | 40S ribosomal protein S2 | R/T/T | -0.81 |
| AAEL000010 | ribosomal protein L36, putative | R/T/T | -0.81 |
| AAEL015304 | conserved hypothetical protein | UNK | -0.81 |
| AAEL007962 | glutathione-s-transferase theta, gst | R/S/M | -0.81 |
| AAEL009825 | 60S ribosomal protein L13a | R/T/T | -0.81 |
| AAEL012855 | lipoprotein | DIV | -0.81 |
| AAEL006702 | fibrinogen and fibronectin | IMM | -0.81 |
| AAEL001401 | membrane glycoprotein LIG-1 | DIV | -0.82 |
| AAEL005298 | aminopeptidase N | R/T/T | -0.82 |
| AAEL010573 | ribosomal protein S25, putative | R/T/T | -0.82 |
| AAEL009994 | 60S ribosomal protein L4 | R/T/T | -0.82 |
| AAEL008329 | 60S ribosomal protein L24 | R/T/T | -0.82 |
| AAEL000227 | epithelial membrane protein | IMM | -0.82 |
| AAEL003716 | ribonuclease UK114, putative | R/T/T | -0.83 |
| AAEL015450 | ribonuclease UK114, putative | R/T/T | -0.83 |
| AAEL009642 | cathepsin b | IMM | -0.83 |
| AAEL000650 | membrane glycoprotein LIG-1 | DIV | -0.83 |
| AAEL002833 | cathepsin l | IMM | -0.83 |
| AAEL010318 | polyadenylate-binding protein | R/T/T | -0.83 |
| AAEL015438 | mannose-6-phosphate isomerase | MET | -0.83 |
| AAEL005823 | Trypsin, putative | PROT | -0.84 |
| AAEL006602 | TPA_inf | DIV | -0.84 |
| AAEL008478 | conserved hypothetical protein | UNK | -0.84 |
| AAEL012029 | CCR4-NOT transcription complex subunit | DIV | -0.84 |
| AAEL005266 | 40S ribosomal protein S14 | R/T/T | -0.84 |
| AAEL008485 | conserved hypothetical protein | UNK | -0.84 |
| AAEL001840 | zinc carboxypeptidase | PROT | -0.84 |
| AAEL006860 | ribosomal protein S28, putative | R/T/T | -0.84 |
| AAEL012686 | ribosomal protein S12, putative | R/T/T | -0.84 |
| AAEL012764 | Glycine N-methyltransferase | DIV | -0.85 |
| AAEL004325 | ribosomal protein L5 | R/T/T | -0.85 |
| AAEL009607 | lingerer | DIV | -0.85 |
| AAEL001173 | amidase | DIG | -0.85 |
| AAEL002889 | hypothetical protein | UNK | -0.85 |
| AAEL003877 | ubiquitin | DIV | -0.85 |
| AAEL009653 | 40S ribosomal protein S30 | R/T/T | -0.85 |
| AAEL007705 | hect E3 ubiquitin ligase | DIV | -0.85 |
| AAEL011746 | succinyl-coa synthetase beta chain | MET | -0.86 |
| AAEL013728 | conserved hypothetical protein | UNK | -0.86 |
| AAEL014583 | 60S acidic ribosomal protein P2 | R/T/T | -0.86 |
| AAEL000258 | conserved hypothetical protein | UNK | -0.86 |
| AAEL014714 | conserved hypothetical protein | UNK | -0.86 |
| AAEL013625 | 40S ribosomal protein S5 | R/T/T | -0.87 |
| AAEL008192 | 40S ribosomal protein S3 | R/T/T | -0.87 |
| AAEL015260 | phosphatidylethanolamine-binding protein, putative | DIV | -0.87 |
| AAEL010756 | 40S ribosomal protein S19 | R/T/T | -0.87 |
| AAEL000221 | mediator complex, subunit, putative | DIV | -0.88 |
| AAEL013515 | Pupal cuticle protein, putative | C/S | -0.88 |
| AAEL002696 | serine protease, putative | PROT | -0.88 |
| AAEL008188 | 60S ribosomal protein L6 | R/T/T | -0.89 |
| AAEL010429 | putative protein G12 | DIV | -0.89 |
| AAEL012860 | conserved hypothetical protein | UNK | -0.89 |
| AAEL015036 | protease S51 alpha-aspartyl dipeptidase | PROT | -0.9 |
| AAEL012417 | transmembrane protein 68 | DIV | -0.9 |
| AAEL002194 | uricase | DIV | -0.9 |
| AAEL002036 | hypothetical protein | UNK | -0.91 |
| AAEL007771 | 60S ribosomal protein L22 | R/T/T | -0.91 |
| AAEL008481 | 60S ribosomal protein L18 | R/T/T | -0.91 |
| AAEL006141 | 40S ribosomal protein S3a | R/T/T | -0.91 |
| AAEL004728 | 5-methyltetrahydrofolate:homocysteine methyltransferase | MET | -0.91 |
| AAEL003530 | acidic ribosomal protein P1, putative | R/T/T | -0.92 |
| AAEL005027 | acidic ribosomal protein P1, putative | R/T/T | -0.92 |
| AAEL001209 | sodium-dependent phosphate transporter | TRP | -0.92 |
| AAEL001844 | zinc carboxypeptidase | PROT | -0.93 |
| AAEL004401 | peroxinectin | IMM | -0.93 |
| AAEL005901 | 40S ribosomal protein S3a | R/T/T | -0.93 |
| AAEL015136 | Niemann-Pick Type C-2, putative | IMM | -0.93 |
| AAEL008103 | 40S ribosomal protein S8 | R/T/T | -0.93 |
| AAEL010758 | hypothetical protein | UNK | -0.93 |
| AAEL011811 | DNA replication licensing factor MCM3 | R/T/T | -0.93 |
| AAEL001668 | enolase | DIV | -0.93 |
| AAEL011408 | galactose-specific C-type lectin, putative | IMM | -0.94 |
| AAEL006668 | FR47-like protein | DIV | -0.94 |
| AAEL004833 | attacin precursor | IMM | -0.94 |
| AAEL009962 | VirB8 | DIV | -0.94 |
| AAEL000105 | beta-alanine synthase, putative | DIV | -0.94 |
| AAEL000032 | ribosomal protein S6 | R/T/T | -0.94 |
| AAEL003467 | conserved hypothetical protein | UNK | -0.94 |
| AAEL013097 | 60S ribosomal protein L23 | R/T/T | -0.94 |
| AAEL013583 | 60S ribosomal protein L23 | R/T/T | -0.94 |
| AAEL015006 | 60S ribosomal protein L23 | R/T/T | -0.94 |
| AAEL005102 | conserved hypothetical protein | UNK | -0.94 |
| AAEL009875 | alanine aminotransferase | DIV | -0.94 |
| AAEL001022 | anterior fat body protein | DIV | -0.95 |
| AAEL000670 | methionine sulfoxide reductase | DIV | -0.95 |
| AAEL002970 | conserved hypothetical protein | UNK | -0.96 |
| AAEL011790 | conserved hypothetical protein | UNK | -0.96 |
| AAEL010684 | trehalose-6-phosphate synthase 1 | DIV | -0.96 |
| AAEL007162 | gaba(a) receptor-associated protein | C/S | -0.96 |
| AAEL014551 | triacylglycerol lipase, pancreatic | MET | -0.96 |
| AAEL009601 | pyridoxine kinase | MET | -0.96 |
| AAEL002200 | fatty acid synthase | MET | -0.96 |
| AAEL011206 | aminoacylase, putative | PROT | -0.96 |
| AAEL005129 | 40S ribosomal protein S30 | R/T/T | -0.96 |
| AAEL015606 | mucin-like peritrophin | C/S | -0.96 |
| AAEL014662 | AMP dependent coa ligase | MET | -0.97 |
| AAEL000647 | predicted protein | DIG | -0.97 |
| AAEL009406 | n(4)-(beta-n-acetylglucosaminyl)-l-asparaginase | PROT | -0.98 |
| AAEL005931 | 6-phosphogluconate dehydrogenase | MET | -0.98 |
| AAEL012439 | POSSIBLE ENOYL-CoA HYDRATASE ECHA12 (ENOYL HYDRASE) (UNSATURATED ACYL-CoA HYDRATASE) (CROTONASE), putative | MET | -0.98 |
| AAEL006070 | phosphoglycerate mutase | DIV | -0.99 |
| AAEL000101 | AMP dependent coa ligase | MET | -1 |
| AAEL010697 | 3-ketoacyl-coa thiolase, mitochondrial (beta- ketothiolase) (acetyl-coa acyltransferase) (mitochondrial 3-oxoacyl- coa thiolase) | R/S/M | -1 |
| AAEL003666 | leukotriene a-4 hydrolase | PROT | -1.01 |
| AAEL005722 | 60S ribosomal protein L7a | R/T/T | -1.01 |
| AAEL004175 | 40S ribosomal protein S17 | R/T/T | -1.01 |
| AAEL007715 | 60S ribosomal protein L21 | R/T/T | -1.01 |
| AAEL011400 | fibrinogen and fibronectin | IMM | -1.01 |
| AAEL009029 | aldehyde dehydrogenase | MET | -1.01 |
| AAEL013525 | Timp-3, putative | MET | -1.01 |
| AAEL003203 | fatty acid desaturase, putative | MET | -1.02 |
| AAEL013158 | 40S ribosomal protein S3a | R/T/T | -1.02 |
| AAEL001402 | membrane glycoprotein LIG-1 | DIV | -1.02 |
| AAEL003872 | translationally controlled tumor protein | R/T/T | -1.02 |
| AAEL001293 | cellular retinaldehyde-binding protein | DIV | -1.02 |
| AAEL012567 | synaptic vesicle protein | DIV | -1.02 |
| AAEL013118 | putative protein G12 | DIV | -1.03 |
| AAEL010921 | organic anion transporter | TRP | -1.03 |
| AAEL000823 | ribosomal protein L35A, putative | R/T/T | -1.03 |
| AAEL004976 | conserved hypothetical protein | UNK | -1.03 |
| AAEL006169 | cathepsin d | IMM | -1.04 |
| AAEL001020 | anterior fat body protein | DIV | -1.04 |
| AAEL003406 | conserved hypothetical protein | UNK | -1.05 |
| AAEL008744 | hypothetical protein | UNK | -1.05 |
| AAEL011447 | 60S ribosomal protein L14 | R/T/T | -1.05 |
| AAEL005097 | cold induced protein (BnC24A), putative | R/T/T | -1.06 |
| AAEL014275 | molybdopterin cofactor sulfurase (mosc) | DIV | -1.06 |
| AAEL005123 | carboxylesterase | R/S/M | -1.06 |
| AAEL003770 | bifunctional coenzyme A synthase | R/S/M | -1.06 |
| AAEL003404 | hypothetical protein | UNK | -1.07 |
| AAEL004987 | odorant binding protein | TRP | -1.07 |
| AAEL013987 | conserved hypothetical protein | UNK | -1.07 |
| AAEL007103 | p37NB protein, putative(LRR) | IMM | -1.07 |
| AAEL010436 | microvilli membrane protein | DIV | -1.07 |
| AAEL009165 | conserved hypothetical protein | UNK | -1.08 |
| AAEL013126 | putative protein G12 | DIV | -1.08 |
| AAEL009038 | prolylcarboxypeptidase, putative | MET | -1.08 |
| AAEL002228 | fatty acid synthase | MET | -1.08 |
| AAEL014309 | conserved hypothetical protein | UNK | -1.08 |
| AAEL004284 | mitochondrial ATPase inhibitor, putative | R/S/M | -1.09 |
| AAEL009899 | conserved hypothetical protein | UNK | -1.09 |
| AAEL012862 | hypothetical protein | UNK | -1.09 |
| AAEL013851 | conserved hypothetical protein | UNK | -1.09 |
| AAEL001307 | SEC14, putative | DIV | -1.09 |
| AAEL008620 | D7 protein, putative | TRP | -1.1 |
| AAEL003160 | conserved hypothetical protein | UNK | -1.11 |
| AAEL002047 | 40S ribosomal protein S10 | R/T/T | -1.11 |
| AAEL013656 | bm-40 precursor | DIV | -1.12 |
| AAEL004841 | conserved hypothetical protein | UNK | -1.12 |
| AAEL004988 | phosphoglycerate kinase | MET | -1.12 |
| AAEL001863 | zinc carboxypeptidase | PROT | -1.13 |
| AAEL009166 | putative protein G12 | DIV | -1.13 |
| AAEL012464 | alanine-glyoxylate aminotransferase | DIV | -1.14 |
| AAEL010028 | sarcosine dehydrogenase | DIV | -1.14 |
| AAEL002908 | hypothetical protein | UNK | -1.14 |
| AAEL013287 | conserved hypothetical protein | UNK | -1.14 |
| AAEL001414 | conserved hypothetical protein | UNK | -1.16 |
| AAEL012357 | conserved hypothetical protein | UNK | -1.16 |
| AAEL009097 | Cathepsin K | PROT | -1.17 |
| AAEL005651 | ethanolamine-phosphate cytidylyltransferase | DIV | -1.18 |
| AAEL002360 | serine-type enodpeptidase, putative | PROT | -1.18 |
| AAEL006598 | serine-type enodpeptidase, putative | DIG | -1.18 |
| AAEL011656 | 40S ribosomal protein S15 | R/T/T | -1.18 |
| AAEL012944 | 60S ribosomal protein L11 | R/T/T | -1.18 |
| AAEL003079 | glucosyl/glucuronosyl transferases | MET | -1.19 |
| AAEL009513 | adenylate cyclase | TRP | -1.21 |
| AAEL002416 | short-chain dehydrogenase | R/S/M | -1.21 |
| AAEL013885 | conserved hypothetical protein | UNK | -1.21 |
| AAEL002283 | Bap3p | DIV | -1.22 |
| AAEL006670 | vitelline membrane protein | DIV | -1.22 |
| AAEL004388 | peroxinectin | IMM | -1.23 |
| AAEL000923 | secreted protein, putative | DIV | -1.23 |
| AAEL006458 | alcohol dehydrogenase | MET | -1.23 |
| AAEL004390 | peroxinectin | IMM | -1.23 |
| AAEL007226 | nidogen | DIV | -1.23 |
| AAEL012858 | hypothetical protein | UNK | -1.25 |
| AAEL014871 | methylenetetrahydrofolate dehydrogenase | MET | -1.26 |
| AAEL006834 | glutamate semialdehyde dehydrogenase | DIV | -1.26 |
| AAEL007439 | myosin light chain 1, putative | C/S | -1.26 |
| AAEL003612 | peroxinectin | IMM | -1.28 |
| AAEL005474 | hypothetical protein | UNK | -1.28 |
| AAEL008574 | acyl-CoA oxidase | MET | -1.28 |
| AAEL012644 | peritrophin-like protein | C/S | -1.3 |
| AAEL004386 | peroxinectin | IMM | -1.3 |
| AAEL005783 | conserved hypothetical protein | UNK | -1.31 |
| AAEL010821 | 60S acidic ribosomal protein P0 | R/T/T | -1.31 |
| AAEL007046 | mitochondrial brown fat uncoupling protein | R/S/M | -1.34 |
| AAEL014936 | sarcosine dehydrogenase | DIV | -1.34 |
| AAEL010751 | methylenetetrahydrofolate dehydrogenase | MET | -1.35 |
| AAEL002920 | NlpC/P60 family protein 1 | DIV | -1.38 |
| AAEL008789 | apolipophorin-III, putative | DIV | -1.39 |
| AAEL004450 | cytochrome b5, putative | R/S/M | -1.41 |
| AAEL000667 | alpha-amylase | DIG | -1.43 |
| AAEL002473 | nonstructural protein 1 | DIV | -1.46 |
| AAEL006446 | trehalose-6-phosphate synthase | DIV | -1.46 |
| AAEL007536 | carnitine o-acyltransferase | DIV | -1.47 |
| AAEL004701 | argininosuccinate synthase | DIV | -1.47 |
| AAEL002662 | elongase, putative | MET | -1.52 |
| AAEL007590 | cathepsin b | IMM | -1.53 |
| AAEL004342 | odorant-binding protein | CSR | -1.65 |
| AAEL007703 | conserved hypothetical protein | UNK | -1.67 |
| AAEL007097 | 4-nitrophenylphosphatase | MET | -1.73 |
| AAEL014452 | acyl-coa dehydrogenase | MET | -1.79 |
| AAEL014556 | conserved hypothetical protein | UNK | -1.83 |
| AAEL015053 | conserved hypothetical protein | UNK | -1.83 |
| AAEL013338 | lethal(2)essential for life protein, l2efl | DIV | -1.89 |
