# Supplementary material for: Transcriptome Analysis of Aedes aegypti Transgenic Mosquitoes with Altered Immunity
Source: PLoS Pathog. 2011 Nov 17;7(11):e1002394. doi: 10.1371/journal.ppat.1002394 (PMC3219725; doi:10.1371/journal.ppat.1002394)
Supplement: Table S2 — Repertoire of genes affected by ectopic expression of REL2 in the fat body of the transgenic Ae. aegypti female mosquitoes. Data obtained by means of a full genome Agilent-based microarray analysis. Gene ID, gene name, functional group and log fold increase (decrease) are presented. Abbreviations for functional groups: IMM, immunity; R/S/M, redox, stress and mitochondrion; DIG, digestive; C/S, cytoskeletal and structural; PROT, proteolysis; TRP, transport; R/T/T, replication, transcription, and translation; MET, metabolism; DIV, diverse functions; UNK, unknown functions. (DOCX) [file ppat.1002394.s007.docx]

Table S2. Repertoire of genes affected by ectopic expression of REL2 in the fat body of the transgenic *Ae. aegypti* female mosquitoes. Data obtained by means of a full genome Agilent-based microarray analysis. Gene ID, gene name, functional group and log fold increase (decrease) are presented. Abbreviations for functional groups: IMM, immunity; R/S/M, redox, stress and mitochondrion; DIG, digestive; C/S, cytoskeletal and structural; PROT, proteolysis; TRP, transport; R/T/T, replication, transcription, and translation; MET, metabolism; DIV, diverse functions; UNK, unknown functions.

| GENE ID | Name | Func group | Logfold |
| --- | --- | --- | --- |
| AAEL003832 | DEFC | IMM | 3.49 |
| AAEL003841 | DEFA | IMM | 2.76 |
| AAEL014382 | galactose-specific C-type lectin, putative | IMM | 2.51 |
| AAEL005792 | CLIPC7 | IMM | 2.28 |
| AAEL011607 | galactose-specific C-type lectin, putative | IMM | 2.24 |
| AAEL010139 | serine protease, putative | IMM | 2.23 |
| AAEL002559 | Cu-Zn-superoxide dismutase precursor | MET | 2.2 |
| AAEL015465 | clip-domain serine protease, putative | IMM | 2.17 |
| AAEL003857 | DEFD | IMM | 2.15 |
| AAEL003614 | CLIPB40 | IMM | 2.14 |
| AAEL005641 | galactose-specific C-type lectin, putative | IMM | 2.02 |
| AAEL012353 | C-type lectin | IMM | 2 |
| AAEL001794 | TEP20 | IMM | 1.93 |
| AAEL001802 | TEP21 | IMM | 1.92 |
| AAEL014390 | galactose-specific C-type lectin, putative | IMM | 1.91 |
| AAEL015639 | transferrin | IMM | 1.89 |
| AAEL008080 | trypsin-eta, putative | DIG | 1.86 |
| AAEL014381 | molybdenum cofactor sulfurase | UNK | 1.77 |
| AAEL002610 | serine protease | IMM | 1.64 |
| AAEL012430 | AMP dependent ligase | MET | 1.54 |
| AAEL005431 | CLIPB37 | IMM | 1.53 |
| AAEL000548 | carboxylesterase | PROT | 1.52 |
| AAEL007624 | REL2 | IMM | 1.49 |
| AAEL008050 | hypothetical protein | UNK | 1.47 |
| AAEL003642 | serine protease | IMM | 1.42 |
| AAEL001508 | Neurotransmitter-gated ion-channel ligand binding | DIV | 1.41 |
| AAEL003712 | LYSC10 | IMM | 1.38 |
| AAEL012086 | APL1B (LRR) | DIV | 1.35 |
| AAEL011619 | galactose-specific C-type lectin, putative | IMM | 1.35 |
| AAEL004591 | hypothetical protein | UNK | 1.34 |
| AAEL014385 | CTLG4 | IMM | 1.33 |
| AAEL014349 | serine protease | IMM | 1.33 |
| AAEL013586 | hypothetical protein | UNK | 1.32 |
| AAEL002585 | serine protease | IMM | 1.26 |
| AAEL000037 | serine protease | IMM | 1.25 |
| AAEL003723 | LYSC11 | IMM | 1.25 |
| AAEL002601 | serine protease, putative | IMM | 1.25 |
| AAEL014300 | hypothetical protein | UNK | 1.25 |
| AAEL014004 | CLIPB39 | IMM | 1.23 |
| AAEL011130 | alcohol dehydrogenase | MET | 1.23 |
| AAEL006585 | clip-domain serine protease, putative | IMM | 1.23 |
| AAEL012534 | hypothetical protein | UNK | 1.22 |
| AAEL005670 | SRPN1 | IMM | 1.21 |
| AAEL004667 | conserved hypothetical protein | UNK | 1.21 |
| AAEL013262 | endochitinase A | DIG | 1.19 |
| AAEL003632 | clip-domain serine protease, putative | IMM | 1.19 |
| AAEL004931 | beta-hexosaminidase b | DIV | 1.18 |
| AAEL002580 | suppressor of tumorigenicity protein 14 | IMM | 1.18 |
| AAEL014139 | proacrosin, putative | MET | 1.18 |
| AAEL003422 | hypothetical protein | UNK | 1.18 |
| AAEL012480 | sodium/calcium exchanger | TRP | 1.17 |
| AAEL009474 | PGRPS1 | IMM | 1.16 |
| AAEL000271 | gamma-glutamyl hydrolase, putative | MET | 1.15 |
| AAEL001627 | UDP-n-acteylglucosamine pyrophosphorylase | DIV | 1.14 |
| AAEL002582 | coagulation factor X, putative | DIV | 1.12 |
| AAEL011404 | galactose-specific C-type lectin, putative | IMM | 1.12 |
| AAEL014079 | SRPN1 | IMM | 1.11 |
| AAEL008468 | cysteine synthase | MET | 1.11 |
| AAEL011610 | galactose-specific C-type lectin, putative | IMM | 1.1 |
| AAEL011616 | serine protease, putative | IMM | 1.1 |
| AAEL003483 | hemolymph protein-like protein | DIV | 1.1 |
| AAEL014387 | coagulation factor X, putative | PROT | 1.09 |
| AAEL008753 | lumican | DIV | 1.07 |
| AAEL000087 | TEP22 | IMM | 1.05 |
| AAEL014494 | methionine-tRNA synthetase | MET | 1.05 |
| AAEL008757 | juvenile hormone esterase | R/S/M | 1.05 |
| AAEL002595 | serine protease | IMM | 1.03 |
| AAEL009859 | nucleolar GTP-binding protein | DIV | 1.01 |
| AAEL005673 | serine protease inhibitor, serpin | IMM | 0.99 |
| AAEL014078 | SRPN2 | IMM | 0.99 |
| AAEL000044 | ornithine decarboxylase | MET | 0.99 |
| AAEL008106 | hypothetical protein | UNK | 0.98 |
| AAEL001423 | acid phosphatase-1 | MET | 0.96 |
| AAEL011137 | succinyl-coa:3-ketoacid-coenzyme a transferase | MET | 0.96 |
| AAEL007626 | GNBPA1 | IMM | 0.95 |
| AAEL004672 | conserved hypothetical protein | UNK | 0.95 |
| AAEL004150 | fibrinogen and fibronectin | DIV | 0.94 |
| AAEL002959 | brain chitinase and chia | MET | 0.94 |
| AAEL011453 | galactose-specific C-type lectin, putative | IMM | 0.93 |
| AAEL000024 | dopachrome-conversion enzyme (DCE), putative | IMM | 0.93 |
| AAEL001052 | heat shock protein, putative | R/S/M | 0.93 |
| AAEL010490 | Htur_4994 | DIV | 0.92 |
| AAEL002704 | SRPN23 | IMM | 0.91 |
| AAEL000038 | serine protease | IMM | 0.9 |
| AAEL005093 | serine protease | IMM | 0.9 |
| AAEL001565 | peptidyl-glycine alpha-amidating monooxygenase | MET | 0.9 |
| AAEL004493 | ribosome biogenesis protein tsr1 (20S rrna accumulation protein 1) | R/T/T | 0.9 |
| AAEL012687 | Juvenile hormone-inducible protein, putative | DIV | 0.89 |
| AAEL007878 | ornithine decarboxylase | MET | 0.89 |
| AAEL002194 | uricase | DIV | 0.88 |
| AAEL012120 | fad oxidoreductase | DIV | 0.88 |
| AAEL003686 | SRPN11 | IMM | 0.88 |
| AAEL013411 | Basement membrane-specific heparan sulfate proteoglycan core protein | C/S | 0.87 |
| AAEL003593 | fatty acid synthase alpha subunit | CSR | 0.87 |
| AAEL009520 | APL1C | DIV | 0.87 |
| AAEL011777 | SRPN8 | IMM | 0.87 |
| AAEL002898 | pterin-4-alpha-carbinolamine dehydratase | R/T/T | 0.86 |
| AAEL006131 | hypothetical protein | UNK | 0.85 |
| AAEL012685 | Juvenile hormone-inducible protein | DIV | 0.84 |
| AAEL003279 | clip-domain serine protease, putative | IMM | 0.84 |
| AAEL003253 | CLIPB13B | IMM | 0.84 |
| AAEL003006 | 2-deoxyglucose-6-phosphate phosphatase | MET | 0.84 |
| AAEL008668 | MASP-2 protein, putative | MET | 0.84 |
| AAEL001289 | permease, putative | TRP | 0.84 |
| AAEL014755 | tep2 | IMM | 0.83 |
| AAEL005777 | conserved hypothetical protein | UNK | 0.83 |
| AAEL008936 | hypothetical protein | UNK | 0.83 |
| AAEL014980 | serine protease inhibitor (serpin-2), putative | IMM | 0.82 |
| AAEL011622 | serine protease, putative | IMM | 0.82 |
| AAEL009018 | cytochrome P450 | R/S/M | 0.82 |
| AAEL003676 | myosin I, putative | C/S | 0.81 |
| AAEL002720 | SRPN20 | IMM | 0.81 |
| AAEL011455 | galactose-specific C-type lectin, putative | IMM | 0.81 |
| AAEL003114 | conserved hypothetical protein | UNK | 0.81 |
| AAEL000438 | epoxide hydrolase | MET | 0.8 |
| AAEL012398 | metallothionein | PROT | 0.8 |
| AAEL003816 | hypothetical protein | UNK | 0.8 |
| AAEL006834 | glutamate semialdehyde dehydrogenase | DIV | -0.8 |
| AAEL007059 | membrane protein | DIV | -0.8 |
| AAEL003153 | zinc finger protein 25 | DIV | -0.8 |
| AAEL008793 | lin-10 protein homolog | DIV | -0.8 |
| AAEL011708 | heat shock protein | R/S/M | -0.8 |
| AAEL002737 | cytochrome c oxidase, subunit VIIC, putative | R/S/M | -0.8 |
| AAEL003862 | histone h2a | R/T/T | -0.8 |
| AAEL005601 | conserved hypothetical protein | UNK | -0.8 |
| AAEL013492 | prophenoloxidase | IMM | -0.81 |
| AAEL008397 | glutathione peroxidase | R/S/M | -0.81 |
| AAEL011802 | allergen, putative | DIV | -0.82 |
| AAEL007226 | nidogen | DIV | -0.82 |
| AAEL002510 | serine hydroxymethyltransferase | DIV | -0.82 |
| AAEL007883 | fk506-binding protein | MET | -0.82 |
| AAEL003903 | acid phosphatase-1 | MET | -0.82 |
| AAEL005521 | prolylcarboxypeptidase | MET | -0.82 |
| AAEL011112 | alcohol dehydrogenase | MET | -0.82 |
| AAEL001209 | sodium-dependent phosphate transporter | TRP | -0.82 |
| AAEL010590 | aldose-1-epimerase | MET | -0.83 |
| AAEL006542 | retinoid-inducible serine carboxypeptidase (serine carboxypeptidase | PROT | -0.83 |
| AAEL014843 | heat shock protein | R/S/M | -0.83 |
| AAEL005693 | mitochondrial NADH:ubiquinone oxidoreductase B16.6 subunit, putative | R/S/M | -0.83 |
| AAEL013935 | conserved hypothetical protein | UNK | -0.83 |
| AAEL001022 | anterior fat body protein | DIV | -0.84 |
| AAEL013613 | pyruvate dehydrogenase | DIV | -0.84 |
| AAEL006949 | suppressor of cytokine signaling 7 | IMM | -0.84 |
| AAEL009637 | cathepsin b | IMM | -0.84 |
| AAEL006458 | alcohol dehydrogenase | MET | -0.84 |
| AAEL014605 | cytochrome P450 | R/S/M | -0.84 |
| AAEL012994 | glucose-6-phosphate isomerase | MET | -0.85 |
| AAEL008802 | conserved hypothetical protein | UNK | -0.85 |
| AAEL014846 | innexin | C/S | -0.86 |
| AAEL013656 | bm-40 precursor | DIV | -0.86 |
| AAEL007590 | cathepsin b | IMM | -0.86 |
| AAEL004755 | 3-2trans-enoyl-CoA isomerase, putative | R/S/M | -0.86 |
| AAEL004450 | cytochrome b5, putative | R/S/M | -0.86 |
| AAEL003345 | argininosuccinate lyase | DIV | -0.87 |
| AAEL011129 | alcohol dehydrogenase | MET | -0.87 |
| AAEL010697 | 3-ketoacyl-coa thiolase, mitochondrial (beta- ketothiolase) (acetyl-coa acyltransferase) (mitochondrial 3-oxoacyl- coa thiolase) | R/S/M | -0.87 |
| AAEL002426 | endoplasmic reticulum metallopeptidase 1 | DIV | -0.88 |
| AAEL001118 | conserved hypothetical protein | UNK | -0.88 |
| AAEL006271 | superoxide dismutase | IMM | -0.89 |
| AAEL006936 | suppressor of cytokine signaling 7 | IMM | -0.9 |
| AAEL008478 | conserved hypothetical protein | UNK | -0.9 |
| AAEL014551 | triacylglycerol lipase, pancreatic | MET | -0.91 |
| AAEL005178 | juvenile hormone esterase | R/S/M | -0.91 |
| AAEL006793 | cytochrome P450 | R/S/M | -0.92 |
| AAEL010284 | aliphatic nitrilase, putative | DIV | -0.93 |
| AAEL008849 | selenophosphate synthase | MET | -0.93 |
| AAEL004126 | sterol desaturase | MET | -0.93 |
| AAEL008166 | malate dehydrogenase | MET | -0.94 |
| AAEL009691 | carboxylase:pyruvate/acetyl-coa/propionyl-coa | MET | -0.94 |
| AAEL014845 | heat shock protein | R/S/M | -0.94 |
| AAEL003154 | aldo-keto reductase | R/S/M | -0.94 |
| AAEL007162 | gaba(a) receptor-associated protein | C/S | -0.95 |
| AAEL010206 | xylulose kinase | MET | -0.95 |
| AAEL013821 | xylulose kinase | MET | -0.95 |
| AAEL005651 | ethanolamine-phosphate cytidylyltransferase | DIV | -0.96 |
| AAEL011608 | peptidoglycan recognition protein-1, putative | IMM | -0.96 |
| AAEL001488 | ribosomal protein L15 | R/T/T | -0.96 |
| AAEL009291 | retinoid-inducible serine carboxypeptidase (serine carboxypeptidase | MET | -0.98 |
| AAEL012433 | short-chain dehydrogenase | R/S/M | -0.98 |
| AAEL008609 | zinc carboxypeptidase | PROT | -0.99 |
| AAEL006070 | phosphoglycerate mutase | DIV | -1.01 |
| AAEL006323 | aminopeptidase N | PROT | -1.02 |
| AAEL005626 | exostosin-2 | DIV | -1.03 |
| AAEL001844 | zinc carboxypeptidase | PROT | -1.03 |
| AAEL013707 | trypsin | DIG | -1.04 |
| AAEL012064 | Niemann-Pick Type C-2, putative | IMM | -1.04 |
| AAEL014662 | AMP dependent coa ligase | MET | -1.04 |
| AAEL013774 | conserved hypothetical protein | UNK | -1.04 |
| AAEL008006 | 3-hydroxyacyl-coa dehyrogenase | MET | -1.05 |
| AAEL014275 | molybdopterin cofactor sulfurase (mosc) | DIV | -1.06 |
| AAEL013623 | trypsin | DIG | -1.06 |
| AAEL004805 | potassium-dependent sodium-calcium exchanger, putative | TRP | -1.07 |
| AAEL013525 | Timp-3, putative | MET | -1.08 |
| AAEL013628 | trypsin-eta, putative | DIG | -1.09 |
| AAEL000105 | beta-alanine synthase, putative | DIV | -1.1 |
| AAEL000101 | AMP dependent coa ligase | MET | -1.1 |
| AAEL012576 | pyruvate kinase | DIV | -1.11 |
| AAEL007097 | 4-nitrophenylphosphatase | MET | -1.11 |
| AAEL000650 | membrane glycoprotein LIG-1 | DIV | -1.12 |
| AAEL009948 | aldehyde dehydrogenase | MET | -1.13 |
| AAEL005221 | internalin A, putative | DIV | -1.14 |
| AAEL014947 | cdp-diacylglycerol--serine o-phosphatidyltransferase | MET | -1.15 |
| AAEL014556 | conserved hypothetical protein | UNK | -1.15 |
| AAEL015053 | conserved hypothetical protein | UNK | -1.15 |
| AAEL015417 | zinc carboxypeptidase | PROT | -1.17 |
| AAEL006668 | FR47-like protein | DIV | -1.18 |
| AAEL005199 | juvenile hormone esterase | DIV | -1.18 |
| AAEL007126 | sugar transporter | TRP | -1.19 |
| AAEL004701 | argininosuccinate synthase | DIV | -1.2 |
| AAEL008167 | aspartate ammonia lyase | DIV | -1.2 |
| AAEL006389 | cathepsin l | IMM | -1.2 |
| AAEL013515 | Pupal cuticle protein, putative | C/S | -1.21 |
| AAEL008485 | conserved hypothetical protein | UNK | -1.21 |
| AAEL001293 | cellular retinaldehyde-binding protein | DIV | -1.22 |
| AAEL011980 | fliB domain protein | DIV | -1.23 |
| AAEL011414 | high mobility group non-histone protein, putative | MET | -1.25 |
| AAEL000459 | Transmembrane protein C2orf18 | DIV | -1.26 |
| AAEL012856 | hypothetical protein | UNK | -1.26 |
| AAEL008598 | melanization-related protein | DIV | -1.27 |
| AAEL008876 | deoxyribonuclease I, putative | DIV | -1.27 |
| AAEL015304 | conserved hypothetical protein | UNK | -1.31 |
| AAEL013611 | AMP dependent ligase | MET | -1.32 |
| AAEL013835 | Niemann-Pick Type C-2 | DIV | -1.33 |
| AAEL000322 | hypothetical protein | UNK | -1.35 |
| AAEL003313 | alkaline phosphatase | DIV | -1.39 |
| AAEL014913 | pyruvate kinase | DIV | -1.4 |
| AAEL005102 | conserved hypothetical protein | UNK | -1.4 |
| AAEL013715 | trypsin | DIG | -1.43 |
| AAEL004390 | peroxinectin | IMM | -1.44 |
| AAEL002381 | high mobility group protein D | DIV | -1.45 |
| AAEL013981 | hexamerin 2 beta | IMM | -1.46 |
| AAEL004798 | mucin-like peritrophin | DIV | -1.5 |
| AAEL015451 | hypothetical protein | UNK | -1.57 |
| AAEL007591 | Niemann-Pick Type C-2, putative | DIV | -1.62 |
| AAEL013717 | sphingomyelin phosphodiesterase | MET | -1.62 |
| AAEL011400 | fibrinogen and fibronectin | IMM | -1.64 |
| AAEL007592 | Niemann-Pick Type C-2, putative | DIV | -1.67 |
| AAEL006381 | sphingomyelin phosphodiesterase | MET | -1.73 |
| AAEL005536 | tetraspanin 29fb | DIV | -1.74 |
| AAEL013936 | serine protease inhibitor, serpin | IMM | -1.77 |
| AAEL008789 | apolipophorin-III, putative | DIV | -1.85 |
| AAEL002495 | putative mucin-like protein | DIV | -1.87 |
| AAEL008093 | trypsin | DIG | -1.88 |
| AAEL012697 | sterol carrier protein-2, putative | MET | -1.9 |
| AAEL015661 | sterol carrier protein-2, putative | MET | -1.9 |
| AAEL004386 | peroxinectin | IMM | -2.03 |
| AAEL010776 | carboxypeptidase | PROT | -2.03 |
| AAEL010203 | trypsin | PROT | -2.04 |
| AAEL009330 | carbonic anhydrase II, putative | MET | -2.05 |
| AAEL012499 | histone h2a | R/T/T | -2.06 |
|  |  |  |  |
| AAEL004388 | peroxinectin | IMM | -2.16 |
| AAEL005835 | tetraspanin 97e | DIV | -2.18 |
| AAEL002889 | hypothetical protein | UNK | -2.2 |
| AAEL001274 | glycine-rich protein-like | DIV | -2.23 |
| AAEL013885 | conserved hypothetical protein | UNK | -2.24 |
| AAEL013712 | trypsin | PROT | -2.25 |
| AAEL007926 | retinoid-inducible serine carboxypeptidase (serine carboxypeptidase | MET | -2.32 |
| AAEL004401 | peroxinectin | IMM | -2.35 |
| AAEL013714 | trypsin, putative | IMM | -2.39 |
| AAEL010195 | trypsin | PROT | -2.46 |
| AAEL010196 | trypsin | PROT | -2.59 |
| AAEL003060 | serine-type enodpeptidase, putative | DIG | -2.67 |
| AAEL001693 | serine-type enodpeptidase, putative | PROT | -2.67 |
| AAEL000859 | LSD1 subclass family protein | DIV | -2.76 |
| AAEL013338 | lethal(2)essential for life protein, l2efl | DIV | -2.81 |
| AAEL001611 | putative protein G12 | DIV | -2.96 |
| AAEL009985 | conserved hypothetical protein | UNK | -2.99 |
| AAEL015136 | Niemann-Pick Type C-2, putative | IMM | -3 |
| AAEL002347 | serine-type enodpeptidase, putative | DIG | -3.04 |
| AAEL013284 | serine-type enodpeptidase, putative | PROT | -3.17 |
| AAEL001690 | serine-type enodpeptidase, putative | PROT | -3.24 |
| AAEL000500 | secreted juvenile hormone binding protein | DIV | -3.35 |
| AAEL010202 | trypsin | DIG | -3.39 |
| AAEL013713 | trypsin | DIG | -3.39 |
| AAEL001701 | serine-type enodpeptidase, putative | PROT | -3.48 |
| AAEL008769 | serine-type enodpeptidase, putative | PROT | -3.48 |
| AAEL013118 | putative protein G12 | DIV | -3.54 |
| AAEL014188 | serine-type enodpeptidase, putative | DIG | -3.54 |
| AAEL013126 | putative protein G12 | DIV | -3.56 |
| AAEL009165 | conserved hypothetical protein | UNK | -3.56 |
| AAEL010338 | methyl-accepting chemotaxis protein | DIV | -3.65 |
| AAEL009166 | putative protein G12 | DIV | -3.69 |
| AAEL013127 | putative protein G12 | DIV | -3.72 |
| AAEL010920 | putative protein G12 | DIV | -3.72 |
| AAEL001621 | conserved hypothetical protein | UNK | -3.72 |
| AAEL006594 | serine-type enodpeptidase, putative | DIG | -3.81 |
| AAEL010782 | carboxypeptidase | PROT | -3.96 |
| AAEL010436 | microvilli membrane protein | DIV | -3.99 |
| AAEL003467 | conserved hypothetical protein | UNK | -4.1 |
| AAEL010429 | putative protein G12 | DIV | -4.18 |
