# Supplementary material for: Transcriptome Analysis of Aedes aegypti Transgenic Mosquitoes with Altered Immunity
Source: PLoS Pathog. 2011 Nov 17;7(11):e1002394. doi: 10.1371/journal.ppat.1002394 (PMC3219725; doi:10.1371/journal.ppat.1002394)
Supplement: Table S3 — Hierarchical clustering of genes regulated by fat body-specific ectopic expression of REL1 and REL2 , RNAi depletions of Cactus (CAC) and Caspar (CASP) in Ae. aegypti females. Abbreviations for functional groups: IMM, immunity; R/S/M, redox, stress and mitochondrion; DIG, digestive; C/S, cytoskeletal and structural; PROT, proteolysis; TRP, transport; R/T/T, replication, transcription, and translation; MET, metabolism; DIV, diverse functions; UNK, unknown functions. (DOCX) [file ppat.1002394.s008.docx]

Table S3. Hierarchical clustering of genes regulated by fat body-specific ectopic expression of *REL1* and *REL2*, RNAi depletions of *Cactus* (CAC) and *Caspar* (CASP) in *Ae. aegypti* females

Abbreviations for functional groups: IMM, immunity; R/S/M, redox, stress and mitochondrion; DIG, digestive; C/S, cytoskeletal and structural; PROT, proteolysis; TRP, transport; R/T/T, replication, transcription, and translation; MET, metabolism; DIV, diverse functions; UNK, unknown functions.

|  |  |  | Logfold | | | |  |
| --- | --- | --- | --- | --- | --- | --- | --- |
| Gene ID | Name | FC | Rel2 | Rel1 | CAC | CASP | Cluster |
| AAEL000024 | dopachrome-conversion enzyme (DCE), putative | IMM | 0.93 | 0.96 | 0.85 | -0.82 | I |
| AAEL001802 | macroglobulin/complement | IMM | 1.92 | 0.93 | 1.7 | -1.23 | I |
| AAEL003723 | lysozyme P, putative | IMM | 1.25 | 1.11 | 0.03 | -1.24 | I |
| AAEL003832 | DEFC | IMM | 3.49 | 1.34 | 0.95 | -1.67 | I |
| AAEL003841 | DEFA | IMM | 2.76 | 1.19 | 0.97 | -2.16 | I |
| AAEL003857 | DEFD | IMM | 2.15 | 1.13 | 1 | -1.96 | I |
| AAEL011404 | galactose-specific C-type lectin, putative | IMM | 1.12 | 0.89 | 0.11 | -0.11 | I |
| AAEL003593 | fatty acid synthase alpha subunit | CSR | 0.87 | 0.25 | 2.72 | -0.35 | I |
| AAEL000038 | serine protease | IMM | 0.9 | -0.03 | 1.44 | 0.18 | I |
| AAEL008050 | hypothetical protein | UNK | 1.47 | 0.3 | 2.33 | -0.07 | I |
| AAEL002585 | serine protease | IMM | 1.26 | 1.1 | 2.29 | 0.22 | I |
| AAEL003253 | clip-domain serine protease, putative | IMM | 0.84 | 0.83 | 1.64 | 0 | I |
| AAEL003632 | clip-domain serine protease, putative | IMM | 1.19 | 1.06 | 2.03 | 0.05 | I |
| AAEL002580 | predicted protein | IMM | 1.18 | 0.85 | 1.74 | 0.22 | I |
| AAEL004672 | conserved hypothetical protein | UNK | 0.95 | 0.65 | 1.39 | -0.03 | I |
| AAEL002720 | serine protease inhibitors, serpins | IMM | 0.81 | 0.45 | 0.98 | 0.24 | I |
| AAEL003279 | clip-domain serine protease, putative | IMM | 0.84 | 0.5 | 1.42 | 0.17 | I |
| AAEL002595 | serine protease | IMM | 1.03 | 0.31 | 1.62 | 0.36 | I |
| AAEL014980 | serine protease inhibitor (serpin-2), putative | IMM | 0.82 | 0.29 | 1.36 | 0.26 | I |
| AAEL006131 | hypothetical protein | UNK | 0.85 | 0.54 | 1.26 | 0.5 | I |
| AAEL002624 | serine protease | IMM | 0.57 | 0.92 | 1.77 | 0.5 | I |
| AAEL002629 | serine protease | IMM | 0.57 | 0.81 | 1.81 | 0.14 | I |
| AAEL011455 | galactose-specific C-type lectin, putative | IMM | 0.81 | 1.04 | 2.47 | 0.22 | I |
| AAEL011453 | galactose-specific C-type lectin, putative | IMM | 0.93 | 1.29 | 2.41 | 0.24 | I |
| AAEL000087 | macroglobulin/complement | IMM | 1.05 | 1.02 | 1.9 | 0.32 | I |
| AAEL011777 | serine protease inhibitor, serpin | IMM | 0.87 | 0.85 | 1.38 | 0.22 | I |
| AAEL008757 | juvenile hormone esterase | R/S/M | 1.05 | 1.17 | 1.91 | 0.17 | I |
| AAEL014755 | tep2 | IMM | 0.83 | 0.93 | 1.63 | 0.17 | I |
| AAEL007107 | serine protease, putative | IMM | 0.79 | 1.17 | 1.59 | 0.24 | I |
| AAEL008607 | tep3 | IMM | 0.73 | 0.96 | 1.4 | 0.19 | I |
| AAEL015527 | conserved hypothetical protein | PROT | 0.68 | 1.11 | 1.47 | 0.13 | I |
| AAEL002301 | serine protease | IMM | 0.72 | 0.9 | 0.93 | 0.21 | I |
| AAEL008404 | Trypsin, putative | DIG | 0.66 | 0.85 | 0.92 | 0.03 | I |
| AAEL002601 | serine protease, putative | IMM | 1.25 | 0.84 | 1.26 | 0.07 | I |
| AAEL004667 | conserved hypothetical protein | UNK | 1.21 | 1.05 | 1.43 | 0.1 | I |
| AAEL001508 | conserved hypothetical protein | UNK | 1.41 | 0.99 | 1.6 | 0.17 | I |
| AAEL014349 | serine protease | IMM | 1.33 | 0.92 | 1.7 | 0.21 | I |
| AAEL014079 | serine protease inhibitor, serpin | IMM | 1.11 | 0.91 | 1 | -0.03 | I |
| AAEL005670 | serine protease inhibitor, serpin | IMM | 1.21 | 0.82 | 0.88 | -0.26 | I |
| AAEL008468 | cysteine synthase | MET | 1.11 | 0.52 | 0.82 | -0.25 | I |
| AAEL001794 | macroglobulin/complement | IMM | 1.93 | 0.85 | 1.52 | 0.31 | I |
| AAEL009474 | peptidoglycan recognition protein-lc isoform | IMM | 1.16 | 0.59 | 0.94 | 0.11 | I |
| AAEL003614 | clip-domain serine protease, putative | IMM | 2.14 | 0.82 | 1.21 | 0.15 | I |
| AAEL005431 | clip-domain serine protease, putative | IMM | 1.53 | 0.49 | 0.9 | 0.03 | I |
| AAEL010139 | serine protease, putative | IMM | 2.23 | 0.74 | 1.5 | 0.03 | I |
| AAEL012353 | C-type lectin | IMM | 2 | 0.9 | 0.84 | 0.13 | I |
| AAEL005641 | galactose-specific C-type lectin, putative | IMM | 2.02 | 0.84 | 2.28 | -0.46 | I |
| AAEL003006 | 2-deoxyglucose-6-phosphate phosphatase | MET | 0.84 | 0.09 | 0.92 | -0.13 | I |
| AAEL001565 | peptidyl-glycine alpha-amidating monooxygenase | MET | 0.9 | 0.2 | 0.92 | 0.09 | I |
| AAEL008080 | trypsin-eta, putative | DIG | 1.86 | 0.41 | 1.02 | -0.41 | I |
| AAEL003642 | serine protease | IMM | 1.42 | 0.27 | 1 | 0.07 | I |
| AAEL007624 | REL2 | IMM | 1.49 | 0.23 | 0.92 | 0.19 | I |
| AAEL014390 | galactose-specific C-type lectin, putative | IMM | 1.91 | 0.37 | 0.94 | 0.19 | I |
| AAEL005093 | serine protease | IMM | 0.9 | 0.06 | 1.12 | 0.34 | I |
| AAEL004591 | hypothetical protein | UNK | 1.34 | -0.04 | 1.08 | 0.23 | I |
| AAEL011610 | galactose-specific C-type lectin, putative | IMM | 1.1 | 0.1 | 0.84 | 0.17 | I |
| AAEL000271 | gamma-glutamyl hydrolase, putative | MET | 1.15 | 0.32 | 0.89 | 0.08 | I |
| AAEL002582 | coagulation factor X, putative | DIV | 1.12 | 0.36 | 0.93 | 0.12 | I |
| AAEL014494 | methionine-tRNA synthetase | MET | 1.05 | 0.42 | 0.85 | 0.05 | I |
| AAEL007626 | gram-negative bacteria binding protein | IMM | 0.95 | 0.37 | 0.95 | 0.15 | I |
| AAEL005792 | CLIPC7 | IMM | 2.28 | 0.58 | 1.85 | 0.41 | I |
| AAEL011619 | galactose-specific C-type lectin, putative | IMM | 1.35 | 0.36 | 1.13 | 0.28 | I |
| AAEL011607 | galactose-specific C-type lectin, putative | IMM | 2.24 | 0.92 | 1.65 | 0.9 | I |
| AAEL014382 | galactose-specific C-type lectin, putative | IMM | 2.51 | 0.9 | 1.76 | 0.89 | I |
| AAEL005194 | fibrinogen and fibronectin | IMM | 0.26 | -0.02 | 1.15 | 0.87 | I |
| AAEL015606 | mucin-like peritrophin | C/S | 0.57 | -0.96 | 1.95 | 0.4 | I |
| AAEL000074 | serine protease | IMM | 0.08 | 0.64 | 1.26 | -1.53 | I |
| AAEL015515 | antibacterial peptide, putative | IMM | -0.16 | 0.21 | 1.39 | -2.99 | I |
| AAEL000059 | proacrosin, putative | MET | 0.29 | 0.84 | 1.22 | 0.01 | I |
| AAEL003243 | serine protease | IMM | 0.36 | 0.94 | 1.76 | 0.05 | I |
| AAEL005064 | serine protease | IMM | 0.24 | 0.91 | 1.55 | 0.32 | I |
| AAEL003889 | gram-negative bacteria binding protein | IMM | 0.18 | 0.85 | 1.12 | 0.21 | I |
| AAEL013933 | serine protease inhibitor, serpin | IMM | 0.14 | 0.96 | 1.3 | 0.16 | I |
| AAEL013937 | serine protease inhibitor, serpin | IMM | 0.07 | 0.87 | 1.12 | 0.3 | I |
| AAEL007696 | embryonic polarity dorsal | IMM | -0.03 | 0.93 | 1.01 | 0.16 | I |
| AAEL008364 | serine protease inhibitor, serpin | IMM | -0.27 | 0.98 | 1.28 | 0.17 | I |
| AAEL009195 | Major facilitator superfamily domain-containing protein | DIV | -0.14 | 0.8 | 2.46 | -0.05 | I |
| AAEL014138 | serine protease inhibitor, serpin | IMM | 0.09 | 0.85 | 1.97 | 0.22 | I |
| AAEL014141 | serine protease inhibitor, serpin | IMM | -0.27 | 0.95 | 2 | 0.21 | I |
| AAEL012471 | protein tyrosine phosphatase, putative | IMM | -0.33 | 1.98 | 1.56 | -0.87 | I |
| AAEL003154 | aldo-keto reductase | R/S/M | -0.94 | 0.48 | 1.43 | -0.07 | I |
| AAEL013936 | serine protease inhibitor, serpin | IMM | -1.77 | 0.33 | 1.43 | 0.16 | I |
| AAEL012697 | sterol carrier protein-2, putative | MET | -1.9 | 0.64 | 1.17 | -0.04 | I |
| AAEL015661 | sterol carrier protein-2, putative | MET | -1.9 | 0.64 | 1.17 | -0.04 | I |
| AAEL004284 | mitochondrial ATPase inhibitor, putative | R/S/M | 0.74 | -1.09 | -1.51 | 0.11 | II |
| AAEL004175 | 40S ribosomal protein S17 | R/T/T | 0.18 | -1.01 | -1.11 | -0.12 | II |
| AAEL005298 | aminopeptidase N | R/T/T | 0.04 | -0.82 | -0.81 | -0.05 | II |
| AAEL005931 | 6-phosphogluconate dehydrogenase | MET | 0.3 | -0.98 | -0.93 | -0.18 | II |
| AAEL003203 | fatty acid desaturase, putative | MET | 0.13 | -1.02 | -0.83 | 0.29 | II |
| AAEL006446 | trehalose-6-phosphate synthase | DIV | 0.11 | -1.46 | -0.91 | 0.31 | II |
| AAEL009994 | 60S ribosomal protein L4 | R/T/T | -0.14 | -0.82 | -0.81 | 0.19 | II |
| AAEL014936 | sarcosine dehydrogenase | DIV | -0.11 | -1.34 | -1.06 | 0.28 | II |
| AAEL003862 | histone h2a | R/T/T | -0.8 | -0.55 | -1.59 | 0.58 | II |
| AAEL002036 | hypothetical protein | UNK | -0.59 | -0.91 | -2.72 | 0.14 | II |
| AAEL011811 | DNA replication licensing factor MCM3 | R/T/T | -0.31 | -0.93 | -1.98 | 0.4 | II |
| AAEL011734 | leucinech transmembrane protein | IMM | -0.17 | -0.02 | -1.36 | 0.84 | II |
| AAEL002704 | serine protease inhibitor (serpin-4), putative | IMM | 0.91 | 0.06 | -0.92 | 0.19 | II |
| AAEL001423 | acid phosphatase-1 | MET | 0.96 | -0.07 | -0.97 | 0.09 | II |
| AAEL004493 | ribosome biogenesis protein tsr1 (20S rrna accumulation protein 1) | R/T/T | 0.9 | -0.1 | -0.97 | 0.14 | II |
| AAEL004150 | fibrinogen and fibronectin | DIV | 0.94 | -0.02 | -1.07 | -0.05 | II |
| AAEL014381 | molybdenum cofactor sulfurase | DIV | 1.77 | 0.34 | -1.21 | -0.26 | II |
| AAEL000044 | ornithine decarboxylase | MET | 0.99 | -0.41 | -1.26 | -0.02 | II |
| AAEL003676 | myosin I, putative | C/S | 0.81 | -0.35 | -1.2 | -0.13 | II |
| AAEL013262 | endochitinase A | DIG | 1.19 | -0.57 | -1.97 | -0.07 | II |
| AAEL012398 | metallothionein | PROT | 0.8 | -0.35 | -1.03 | -0.55 | II |
| AAEL000685 | conserved hypothetical protein | UNK | 0.23 | -0.19 | -1.06 | -0.8 | II |
| AAEL003245 | Inhibitor of nuclear factor kappa B kinase beta subunit | IMM | -0.28 | -0.02 | -0.96 | -1.24 | II |
| AAEL002124 | serine protease | IMM | -0.2 | -0.08 | -1.15 | -0.82 | II |
| AAEL011629 | tyrosyl-dna phosphodiesterase | DIV | -0.16 | 0.09 | -0.97 | -0.82 | II |
| AAEL015141 | tyrosyl-dna phosphodiesterase | DIV | -0.16 | 0.09 | -0.97 | -0.82 | II |
| AAEL014863 | glycogenin | MET | -0.13 | -0.13 | -0.89 | -0.85 | II |
| AAEL006811 | cytochrome P450 | R/S/M | -0.34 | 0.22 | -1.79 | -1.04 | II |
| AAEL014608 | cytochrome P450 | R/S/M | -0.34 | 0.22 | -1.79 | -1.04 | II |
| AAEL012073 | nad dehydrogenase | DIV | 0.09 | 0.21 | -0.9 | -0.83 | II |
| AAEL000101 | AMP dependent coa ligase | MET | -1.1 | -1 | -0.99 | -0.47 | III |
| AAEL006070 | phosphoglycerate mutase | DIV | -1.01 | -0.99 | -0.92 | -0.26 | III |
| AAEL006668 | FR47-like protein | DIV | -1.18 | -0.94 | -1.35 | -0.37 | III |
| AAEL000258 | conserved hypothetical protein | UNK | -0.79 | -0.86 | -1.01 | -0.56 | III |
| AAEL011129 | alcohol dehydrogenase | MET | -0.87 | -0.7 | -1.21 | -0.55 | III |
| AAEL011400 | fibrinogen and fibronectin | IMM | -1.64 | -1.01 | -1 | -1.02 | III |
| AAEL002200 | fatty acid synthase | MET | -0.34 | -0.96 | -0.57 | -0.92 | III |
| AAEL004813 | M-phase phosphoprotein, putative | DIV | -0.38 | -0.39 | -0.82 | -0.84 | III |
| AAEL006224 | short-chain dehydrogenase | R/S/M | -0.56 | -0.77 | -1.31 | -0.93 | III |
| AAEL007705 | hect E3 ubiquitin ligase | DIV | -0.62 | -0.85 | -1.31 | -0.88 | III |
| AAEL012207 | myosin light chain 1, putative | C/S | -0.4 | -0.65 | -0.91 | -0.84 | III |
| AAEL000105 | beta-alanine synthase, putative | DIV | -1.1 | -0.94 | -0.47 | 0.15 | III |
| AAEL000650 | membrane glycoprotein LIG-1 | DIV | -1.12 | -0.83 | -0.62 | 0.22 | III |
| AAEL005102 | conserved hypothetical protein | UNK | -1.4 | -0.94 | -0.71 | 0.3 | III |
| AAEL004386 | peroxinectin | IMM | -2.03 | -1.3 | -1.75 | 0.05 | III |
| AAEL004388 | peroxinectin | IMM | -2.16 | -1.23 | -2.05 | 0.09 | III |
| AAEL013338 | lethal(2)essential for life protein, l2efl | DIV | -2.81 | -1.89 | -2.95 | 0.39 | III |
| AAEL004390 | peroxinectin | IMM | -1.44 | -1.23 | -1.36 | 0.27 | III |
| AAEL001293 | cellular retinaldehyde-binding protein | DIV | -1.22 | -1.02 | -1.44 | 0 | III |
| AAEL014843 | heat shock protein | R/S/M | -0.83 | -0.63 | -0.84 | 0 | III |
| AAEL001844 | zinc carboxypeptidase | PROT | -1.03 | -0.93 | -1.14 | 0.12 | III |
| AAEL014662 | AMP dependent coa ligase | MET | -1.04 | -0.97 | -1.26 | 0.12 | III |
| AAEL006834 | glutamate semialdehyde dehydrogenase | DIV | -0.8 | -1.26 | -0.53 | -0.1 | III |
| AAEL005651 | ethanolamine-phosphate cytidylyltransferase | DIV | -0.96 | -1.18 | -0.85 | -0.16 | III |
| AAEL001022 | anterior fat body protein | DIV | -0.84 | -0.95 | -0.52 | 0.02 | III |
| AAEL013656 | bm-40 precursor | DIV | -0.86 | -1.12 | -0.78 | 0.05 | III |
| AAEL014275 | molybdopterin cofactor sulfurase (mosc) | DIV | -1.06 | -1.06 | -0.87 | 0.05 | III |
| AAEL007097 | 4-nitrophenylphosphatase | MET | -1.11 | -1.73 | -1.3 | 0.6 | III |
| AAEL001414 | conserved hypothetical protein | UNK | -0.77 | -1.16 | -1.06 | 0.11 | III |
| AAEL004450 | cytochrome b5, putative | R/S/M | -0.86 | -1.41 | -1.09 | -0.05 | III |
| AAEL014556 | conserved hypothetical protein | UNK | -1.15 | -1.83 | -1.63 | -0.07 | III |
| AAEL015053 | conserved hypothetical protein | UNK | -1.15 | -1.83 | -1.63 | -0.07 | III |
| AAEL001020 | anterior fat body protein | DIV | -0.26 | -1.04 | -1.19 | -0.67 | III |
| AAEL002228 | fatty acid synthase | MET | -0.5 | -1.08 | -1.04 | -0.75 | III |
| AAEL002416 | short-chain dehydrogenase | R/S/M | -0.33 | -1.21 | -1.03 | -0.34 | III |
| AAEL002970 | conserved hypothetical protein | UNK | -0.24 | -0.96 | -0.81 | -0.18 | III |
| AAEL011790 | conserved hypothetical protein | UNK | -0.24 | -0.96 | -0.81 | -0.18 | III |
| AAEL007715 | 60S ribosomal protein L21 | R/T/T | -0.41 | -1.01 | -0.9 | -0.18 | III |
| AAEL011206 | aminoacylase, putative | PROT | -0.32 | -0.96 | -1 | -0.14 | III |
| AAEL005474 | hypothetical protein | UNK | -0.59 | -1.28 | -0.9 | -0.36 | III |
| AAEL014452 | acyl-coa dehydrogenase | MET | -0.79 | -1.79 | -1.57 | -0.63 | III |
| AAEL000227 | epithelial membrane protein | IMM | -0.46 | -0.82 | -0.84 | 0.03 | III |
| AAEL010684 | trehalose-6-phosphate synthase 1 | DIV | -0.44 | -0.96 | -1.07 | 0.04 | III |
| AAEL002283 | Bap3p | DIV | -0.31 | -1.22 | -1.2 | 0.05 | III |
| AAEL001668 | enolase | DIV | -0.35 | -0.93 | -0.98 | 0.15 | III |
| AAEL009601 | pyridoxine kinase | MET | -0.35 | -0.96 | -0.99 | 0.15 | III |
| AAEL008329 | 60S ribosomal protein L24 | R/T/T | -0.3 | -0.82 | -0.82 | 0.06 | III |
| AAEL004701 | argininosuccinate synthase | DIV | -1.2 | -1.47 | -2.25 | -0.06 | III |
| AAEL001209 | sodium-dependent phosphate transporter | TRP | -0.82 | -0.92 | -1.25 | 0.04 | III |
| AAEL010318 | polyadenylate-binding protein | R/T/T | -0.7 | -0.83 | -0.97 | 0.01 | III |
| AAEL006458 | alcohol dehydrogenase | MET | -0.84 | -1.23 | -1.34 | 0.05 | III |
| AAEL007226 | nidogen | DIV | -0.82 | -1.23 | -1.35 | 0.05 | III |
| AAEL009029 | aldehyde dehydrogenase | MET | -0.76 | -1.01 | -1.21 | 0.06 | III |
| AAEL007962 | glutathione-s-transferase theta, gst | R/S/M | -0.53 | -0.81 | -0.99 | 0.02 | III |
| AAEL004988 | phosphoglycerate kinase | MET | -0.76 | -1.12 | -1.37 | -0.08 | III |
| AAEL012029 | CCR4-NOT transcription complex subunit | DIV | -0.58 | -0.84 | -0.96 | -0.11 | III |
| AAEL006081 | nadph fad oxidoreductase | R/S/M | -0.37 | -0.81 | -1.25 | -0.25 | III |
| AAEL009607 | lingerer | DIV | -0.44 | -0.85 | -1.55 | -0.37 | III |
| AAEL004987 | conserved hypothetical protein | TRP | -0.15 | -1.07 | -1.33 | -0.21 | III |
| AAEL010756 | 40S ribosomal protein S19 | R/T/T | -0.2 | -0.87 | -1.1 | -0.33 | III |
| AAEL008620 | D7 protein, putative | TRP | -0.5 | -1.1 | -1.64 | 0.28 | III |
| AAEL009406 | n(4)-(beta-n-acetylglucosaminyl)-l-asparaginase | PROT | -0.12 | -0.98 | -1.25 | 0.01 | III |
| AAEL012464 | alanine-glyoxylate aminotransferase | DIV | -0.14 | -1.14 | -1.33 | 0.14 | III |
| AAEL012860 | conserved hypothetical protein | UNK | -0.15 | -0.89 | -1.49 | 0.06 | III |
| AAEL000859 | LSD1 subclass family protein | DIV | -2.76 | 0.14 | -1.91 | -1.29 | III |
| AAEL002510 | serine hydroxymethyltransferase | DIV | -0.82 | -0.15 | -1.01 | -0.25 | III |
| AAEL003313 | alkaline phosphatase | DIV | -1.39 | -0.38 | -1.64 | -0.82 | III |
| AAEL009691 | carboxylase:pyruvate/acetyl-coa/propionyl-coa | MET | -0.94 | -0.05 | -1.35 | -0.61 | III |
| AAEL003153 | zinc finger protein 25 | DIV | -0.8 | -0.03 | -1.16 | -0.92 | III |
| AAEL013774 | conserved hypothetical protein | UNK | -1.04 | 0.24 | -1.02 | -1.25 | III |
| AAEL002381 | high mobility group protein D | DIV | -1.45 | -0.47 | -1.31 | 0.22 | III |
| AAEL004401 | peroxinectin | IMM | -2.35 | -0.93 | -1.74 | 0.11 | III |
| AAEL006271 | superoxide dismutase | IMM | -0.89 | -0.26 | -1.1 | 0 | III |
| AAEL006949 | suppressor of cytokine signaling 7 | IMM | -0.84 | -0.22 | -0.97 | 0.01 | III |
| AAEL006936 | suppressor of cytokine signaling 7 | IMM | -0.9 | -0.32 | -1.06 | -0.15 | III |
| AAEL002426 | endoplasmic reticulum metallopeptidase 1 | DIV | -0.88 | -0.28 | -0.83 | 0.02 | III |
| AAEL014845 | heat shock protein | R/S/M | -0.94 | -0.34 | -0.9 | -0.07 | III |
| AAEL008093 | trypsin | DIG | -1.88 | 0.01 | -1.25 | -0.09 | III |
| AAEL005693 | mitochondrial NADH:ubiquinone oxidoreductase B16.6 subunit, putative | R/S/M | -0.83 | -0.09 | -0.84 | 0.03 | III |
| AAEL010206 | xylulose kinase | MET | -0.95 | -0.11 | -0.85 | 0.13 | III |
| AAEL013821 | xylulose kinase | MET | -0.95 | -0.11 | -0.85 | 0.13 | III |
| AAEL005536 | tetraspanin 29fb | DIV | -1.74 | -0.64 | -0.96 | 0.14 | III |
| AAEL009985 | conserved hypothetical protein | UNK | -2.99 | -0.69 | -1.41 | 0.19 | III |
| AAEL015136 | Niemann-Pick Type C-2, putative | IMM | -3 | -0.93 | -1.38 | -0.27 | III |
| AAEL010284 | aliphatic nitrilase, putative | DIV | -0.93 | -0.41 | -0.8 | -0.16 | III |
| AAEL013885 | conserved hypothetical protein | UNK | -2.24 | -1.21 | -1.57 | -0.66 | III |
| AAEL006793 | cytochrome P450 | R/S/M | -0.92 | -0.55 | -1.54 | -0.49 | III |
| AAEL006323 | aminopeptidase N | PROT | -1.02 | -0.79 | -1.98 | -0.05 | III |
| AAEL014605 | cytochrome P450 | R/S/M | -0.84 | -0.44 | -1.56 | -0.2 | III |
| AAEL004126 | sterol desaturase | MET | -0.93 | -0.27 | -1.49 | 0.16 | III |
| AAEL013623 | trypsin | DIG | -1.06 | -0.11 | -1.37 | 0.09 | III |
| AAEL013628 | trypsin-eta, putative | DIG | -1.09 | -0.1 | -1.26 | 0.14 | III |
| AAEL000500 | secreted juvenile hormone binding protein | DIV | -3.35 | -0.01 | -1.13 | -0.57 | IV |
| AAEL006594 | serine-type enodpeptidase, putative | DIG | -3.81 | -0.64 | -1.05 | -0.44 | IV |
| AAEL010196 | trypsin | PROT | -2.59 | -0.23 | 0.01 | -0.88 | IV |
| AAEL003060 | serine-type enodpeptidase, putative | DIG | -2.67 | 0.29 | -1.42 | 0.21 | IV |
| AAEL009330 | carbonic anhydrase II, putative | MET | -2.05 | 0.08 | -0.97 | 0.26 | IV |
| AAEL014188 | serine-type enodpeptidase, putative | DIG | -3.54 | 0.34 | -1.02 | 0.2 | IV |
| AAEL008609 | zinc carboxypeptidase | PROT | -0.99 | 0.9 | -0.2 | -0.02 | IV |
| AAEL002889 | hypothetical protein | UNK | -2.2 | -0.85 | -0.08 | 0.49 | IV |
| AAEL008485 | conserved hypothetical protein | UNK | -1.21 | -0.84 | 0.01 | 0.24 | IV |
| AAEL003467 | conserved hypothetical protein | UNK | -4.1 | -0.94 | -0.64 | 0.15 | IV |
| AAEL009165 | conserved hypothetical protein | UNK | -3.56 | -1.08 | -0.17 | 0.35 | IV |
| AAEL013126 | putative protein G12 | DIV | -3.56 | -1.08 | -0.17 | 0.35 | IV |
| AAEL013118 | putative protein G12 | DIV | -3.54 | -1.03 | 0.05 | 0.19 | IV |
| AAEL010429 | putative protein G12 | DIV | -4.18 | -0.89 | 0.11 | -0.4 | IV |
| AAEL008478 | conserved hypothetical protein | UNK | -0.9 | -0.84 | -0.24 | -0.06 | IV |
| AAEL010697 | 3-ketoacyl-coa thiolase, mitochondrial (beta- ketothiolase) (acetyl-coa acyltransferase) (mitochondrial 3-oxoacyl- coa thiolase) | R/S/M | -0.87 | -1 | -0.19 | -0.13 | IV |
| AAEL007162 | gaba(a) receptor-associated protein | C/S | -0.95 | -0.96 | -0.12 | 0.27 | IV |
| AAEL014551 | triacylglycerol lipase, pancreatic | MET | -0.91 | -0.96 | 0.18 | 0.31 | IV |
| AAEL013525 | Timp-3, putative | MET | -1.08 | -1.01 | 0.82 | -0.07 | IV |
