# Supplementary material for: Transcriptome Analysis of Aedes aegypti Transgenic Mosquitoes with Altered Immunity
Source: PLoS Pathog. 2011 Nov 17;7(11):e1002394. doi: 10.1371/journal.ppat.1002394 (PMC3219725; doi:10.1371/journal.ppat.1002394)
Supplement: Table S4 — Repertoire of genes found in both REL1- and REL2-affected fat body transcriptomes in transgenic Ae. aegypti female mosquitoes. Gene ID, gene name, functional group and log fold increase (decrease) are presented. IMM, immunity; R/S/M, redox, stress and mitochondrion; C/S, cytoskeletal and structural; PROT, proteolysis; TRP, transport; R/T/T, replication, transcription, and translation; MET, metabolism; DIV, diverse functions; UNK, unknown functions. (DOCX) [file ppat.1002394.s009.docx]

Table S4. Repertoire of genes found in both REL1- and REL2-affected fat body transcriptomes in transgenic *Ae. aegypti* female mosquitoes. Gene ID, gene name, functional group and log fold increase (decrease) are presented. IMM, immunity; R/S/M, redox, stress and mitochondrion; C/S, cytoskeletal and structural; PROT, proteolysis; TRP, transport; R/T/T, replication, transcription, and translation; MET, metabolism; DIV, diverse functions; UNK, unknown functions.

|  |  |  | Logfold | |
| --- | --- | --- | --- | --- |
| **GENE ID** | **Name** | **FC** | **REL2** | **REL1** |
| AAEL003841 | DEFA | IMM | 2.76 | 1.19 |
| AAEL003832 | DEFC | IMM | 3.49 | 1.34 |
| AAEL003857 | DEFD | IMM | 2.15 | 1.13 |
| AAEL003723 | LYSC11 | IMM | 1.25 | 1.11 |
| AAEL001794 | TEP20 | IMM | 1.93 | 0.85 |
| AAEL001802 | TEP21 | IMM | 1.92 | 0.93 |
| AAEL000087 | TEP22 | IMM | 1.05 | 1.02 |
| AAEL014349 | serine protease | IMM | 1.33 | 0.92 |
| AAEL012353 | C-type lectin | IMM | 2.00 | 0.90 |
| AAEL002585 | serine protease | IMM | 1.26 | 1.10 |
| AAEL002601 | serine protease, putative | IMM | 1.25 | 0.84 |
| AAEL003614 | CLIPB40 | IMM | 2.14 | 0.82 |
| AAEL005670 | serpin-1 | IMM | 1.21 | 0.82 |
| AAEL014079 | serpin-1 | IMM | 1.11 | 0.91 |
| AAEL011777 | serpin-16 | IMM | 0.87 | 0.85 |
| AAEL008757 | juvenile hormone esterase | R/S/M | 1.05 | 1.17 |
| AAEL004667 | conserved hypothetical protein | UNK | 1.21 | 1.05 |
| AAEL003816 | hypothetical protein | UNK | 0.80 | 0.83 |
| AAEL007590 | cathepsin b | IMM | -0.86 | -1.53 |
| AAEL011400 | fibrinogen and fibronectin | IMM | -1.64 | -1.01 |
| AAEL004388 | HPX8A | IMM | -2.16 | -1.23 |
| AAEL004390 | HPX8B | IMM | -1.44 | -1.23 |
| AAEL004386 | HPX8C | IMM | -2.03 | -1.30 |
| AAEL015136 | ML6 | IMM | -3.00 | -0.93 |
| AAEL007097 | 4-nitrophenylphosphatase | MET | -1.11 | -1.73 |
| AAEL000101 | AMP dependent coa ligase | MET | -1.10 | -1.00 |
| AAEL013525 | Timp-3, putative | MET | -1.08 | -1.01 |
| AAEL014662 | AMP dependent coa ligase | MET | -1.04 | -0.97 |
| AAEL014551 | triacylglycerol lipase, pancreatic | MET | -0.91 | -0.96 |
| AAEL006458 | alcohol dehydrogenase | MET | -0.84 | -1.23 |
| AAEL008609 | zinc carboxypeptidase | PROT | -0.99 | 0.90 |
| AAEL001844 | zinc carboxypeptidase | PROT | -1.03 | -0.93 |
| AAEL010697 | acetyl-coa acyltransferase | R/S/M | -0.87 | -1.00 |
| AAEL004450 | cytochrome b5, putative | R/S/M | -0.86 | -1.41 |
| AAEL001209 | sodium-dependent phosphate transporter | TRP | -0.82 | -0.92 |
| AAEL001508 | Neurotransmitter-gated ion-channel ligand binding | DIV | 1.41 | 0.99 |
| AAEL009165 | putative protein G12 | DIV | -3.56 | -1.08 |
| AAEL005102 | ester hydrolase C11orf54-like protein | DIV | -1.40 | -0.94 |
| AAEL015304 | ester hydrolase C11orf54-like protein | DIV | -1.31 | -0.81 |
| AAEL002889 | hypothetical protein | UNK | -2.20 | -0.85 |
|  |  |  |  |  |
