# Supplementary material for: Transcriptome Analysis of Aedes aegypti Transgenic Mosquitoes with Altered Immunity
Source: PLoS Pathog. 2011 Nov 17;7(11):e1002394. doi: 10.1371/journal.ppat.1002394 (PMC3219725; doi:10.1371/journal.ppat.1002394)
Supplement: Table S5 — Repertoires of genes putatively related to the melanization pathway in Ae. aegypti female mosquitoes. Data from transcriptome analyses after - REL1-fat body specific ectopic expression (REL1); REL2-fat body specific ectopic expression (REL2), Plasmodium gallinaceum-infected mosquito midgut (Pg midgut); Pl. gallinaceum-infected mosquito fat body (Pg FB); RNAi depletion of PIAS; RNAi depletion of cactus (CAC); RNAi depletion of caspar (CASP). (DOCX) [file ppat.1002394.s010.docx]

Table S5. Repertoires of genes putatively related to the melanization pathway in *Ae. aegypti* female mosquitoes. Data from transcriptomes after - REL1-fat body specific ectopic expression (REL1); REL2-fat body specific ectopic expression (REL2), *Plasmodium gallinaceum*-infected mosquito midgut (Pg midgut); Pl. *gallinaceum*-infected mosquito fat body (Pg FB); RNAi depletion of PIAS; RNAi depletion of cactus (CAC); RNAi depletion of caspar (CASP)

| GENE ID | PNAME | REL2 | REL1 | Pg midgut | Pg FB | PIAS | CAC | CASP | Name |
| --- | --- | --- | --- | --- | --- | --- | --- | --- | --- |
| AAEL002601 | CLIPA1 | 1.25 | 0.84 |  |  |  | 1.26 |  | Clip domain serine protease homolog |
| AAEL001675 | CLIPA10 |  |  |  |  |  |  |  | Clip domain serine protease homolog |
| AAEL002126 | CLIPA15 |  |  |  |  |  |  |  | Clip domain serine protease homolog |
| AAEL008404 | CLIPA16 |  | 0.85 |  |  |  | 0.92 |  | Clip domain serine protease homolog |
| AAEL007006 | CLIPA17 |  |  |  | -0.81 |  |  |  | Clip domain serine protease homolog |
| AAEL005718 | CLIPA3 |  |  | -1.04 |  |  |  |  | Clip domain serine protease homolog |
| AAEL002288 | CLIPA4 |  |  |  |  |  |  |  | Clip domain serine protease homolog |
| AAEL002301 | CLIPA5 |  | 0.90 | -1.20 |  |  | 0.93 |  | Clip domain serine protease homolog |
| AAEL002585 | CLIPA11 | 1.26 | 1.10 |  |  |  | 2.29 |  | Clip domain serine protease homolog |
| AAEL002590 | CLIPA12 |  |  |  |  |  | 0.84 |  | Clip domain serine protease homolog |
| AAEL002595 | CLIPA14 | 1.03 |  |  |  |  | 1.62 |  | Clip domain serine protease homolog |
| AAEL002629 | CLIPA6 |  | 0.81 | -0.84 |  |  | 1.81 |  | Clip domain serine protease homolog |
| AAEL000074 | CLIPB1 |  |  | -0.97 |  |  | 1.26 | -1.53 | Clip domain serine protease |
| AAEL003243 | CLIPB13A |  | 0.94 | -0.91 |  |  | 1.76 |  | Clip domain serine protease |
| AAEL003253 | CLIPB13B | 0.84 | 0.83 | -1.12 | -0.91 |  | 1.64 |  | Clip domain serine protease |
| AAEL014349 | CLIPB15 | 1.33 | 0.92 |  | 1.58 |  | 1.70 |  | Clip domain serine protease |
| AAEL005648 | CLIPB16 |  |  |  |  |  |  |  | Clip domain serine protease |
| AAEL000059 | CLIPB19 |  | 0.84 | 1.17 |  |  | 1.22 |  | Clip domain serine protease |
| AAEL001084 | CLIPB21 |  |  |  |  |  |  |  | Clip domain serine protease |
| AAEL008668 | CLIPB22 | 0.84 |  |  |  |  |  |  | Clip domain serine protease |
| AAEL012785 | CLIPB23 |  |  | -1.07 |  |  | 1.42 |  | Clip domain serine protease |
| AAEL014140 | CLIPB24 |  |  |  |  |  |  |  | Clip domain serine protease |
| AAEL014137 | CLIPB25 |  |  |  |  |  |  |  | Clip domain serine protease |
| AAEL003280 | CLIPB26 |  |  |  |  |  | 1.19 |  | Clip domain serine protease |
| AAEL007993 | CLIPB27 |  |  |  |  |  | 0.86 |  | Clip domain serine protease |
| AAEL013245 | CLIPB28 | 1.30 |  |  |  |  | 1.53 |  | Clip domain serine protease |
| AAEL006674 | CLIPB29 |  |  |  | 0.92 |  | 0.83 |  | Clip domain serine protease |
| AAEL000760 | CLIPB30 |  |  | -0.93 |  |  |  |  | Clip domain serine protease |
| AAEL006161 | CLIPB31 |  |  |  |  |  |  |  | Clip domain serine protease |
| AAEL000086 | CLIPB32 |  |  |  |  |  |  |  | Clip domain serine protease |
| AAEL000099 | CLIPB33 |  |  | -1.48 |  |  | 0.88 |  | Clip domain serine protease |
| AAEL000028 | CLIPB34 |  |  |  |  |  | 1.04 |  | Clip domain serine protease |
| AAEL000037 | CLIPB35 | 1.25 |  |  |  |  |  |  | Clip domain serine protease |
| AAEL005431 | CLIPB37 | 1.53 |  |  |  |  | 0.90 |  | Clip domain serine protease |
| AAEL003628 | CLIPB38 |  |  |  |  |  | 0.97 |  | Clip domain serine protease |
| AAEL003632 | CLIPB39 | 1.19 | 1.06 | 1.41 |  |  | 2.03 |  | Clip domain serine protease |
| AAEL003614 | CLIPB40 | 2.14 | 0.82 |  |  |  | 1.21 |  | Clip domain serine protease |
| AAEL003631 | CLIPB41 |  |  | 1.10 |  |  |  |  | Clip domain serine protease |
| AAEL006168 | CLIPB42 |  |  |  |  |  | 0.97 |  | Clip domain serine protease |
| AAEL014354 | CLIPB43 |  |  |  |  |  | 1.20 |  | Clip domain serine protease |
| AAEL005060 | CLIPB44 |  |  |  |  |  |  |  | Clip domain serine protease |
| AAEL001077 | CLIPB45 |  |  | -1.00 |  |  | 1.54 |  | Clip domain serine protease |
| AAEL005093 | CLIPB46 | 0.90 |  |  |  |  | 1.12 |  | Clip domain serine protease |
| AAEL005064 | CLIPB5 |  | 0.91 | -1.52 |  |  | 1.55 |  | Clip domain serine protease |
| AAEL000038 | CLIPB6-B36 | 0.90 |  |  |  |  | 1.44 |  | Clip domain serine protease |
| AAEL003625 | CLIPB8 |  |  |  |  |  |  |  | Clip domain serine protease |
| AAEL003610 | CLIPB9 |  |  |  |  |  |  |  | Clip domain serine protease |
| AAEL003642 | CLIPB10 | 1.42 |  |  | -0.85 |  | 1.00 |  | Clip domain serine protease |
| AAEL006371 | CLIPB47-76 |  |  |  |  |  |  |  | Clip domain serine protease |
| AAEL006427 | CLIPB48-77 |  |  |  |  |  |  |  | Clip domain serine protease |
| AAEL007992 | CLIPB49-78 |  |  |  |  |  | 2.03 |  | Clip domain serine protease |
| AAEL014139 | CLIPB50-79 | 1.18 | 1.16 |  |  |  | 2.34 | 0.84 | Clip domain serine protease |
| AAEL011991 | CLIPC1 |  |  | -1.03 |  |  | 1.30 |  | Clip domain serine protease |
| AAEL011593 | CLIPC11 |  |  |  |  |  | 1.11 |  | Clip domain serine protease |
| AAEL012711 | CLIPC12 |  |  |  | 0.82 |  |  |  | Clip domain serine protease |
| AAEL012712 | CLIPC13 |  |  |  |  |  | 1.54 |  | Clip domain serine protease |
| AAEL004948 | CLIPC14 |  |  | 0.84 |  |  |  | -0.95 | Clip domain serine protease |
| AAEL010270 | CLIPC15 |  |  |  |  |  |  |  | Clip domain serine protease |
| AAEL012713 | CLIPC16 |  |  |  |  |  |  |  | Clip domain serine protease |
| AAEL007593 | CLIPC2 |  |  | -0.93 |  |  | 1.38 |  | Clip domain serine protease |
| AAEL007597 | CLIPC3 |  |  |  |  |  |  |  | Clip domain serine protease |
| AAEL004518 | CLIPC5A |  |  |  |  |  | 0.91 |  | Clip domain serine protease |
| AAEL004524 | CLIPC5B |  |  | -1.17 |  |  |  |  | Clip domain serine protease |
| AAEL004540 | CLIPC6 |  |  |  |  |  | 1.32 |  | Clip domain serine protease |
| AAEL007796 | CLIPD1 |  |  |  |  |  |  |  | Clip domain serine protease |
| AAEL015109 | CLIPD10 |  |  | -1.14 |  |  |  |  | Clip domain serine protease |
| AAEL011375 | CLIPD11 |  |  |  |  |  |  |  | Clip domain serine protease |
| AAEL004979 | CLIPD2 |  |  |  |  |  |  |  | Clip domain serine protease |
| AAEL002997 | CLIPD3 |  |  | -1.04 |  |  |  |  | Clip domain serine protease |
| AAEL002124 | CLIPD6 |  |  |  |  |  | -1.15 | -0.82 | Clip domain serine protease |
| AAEL015439 | CLIPD7 |  |  |  |  |  |  |  | Clip domain serine protease |
| AAEL000238 | CLIPD9 |  |  |  |  |  |  |  | Clip domain serine protease |
| AAEL010773 | CLIPE10 |  |  |  |  |  |  |  | Clip domain serine protease homolog |
| AAEL005800 | CLIPE11 |  |  |  |  |  | 2.64 |  | Clip domain serine protease homolog |
| AAEL005644 | CLIPE12 |  |  |  |  |  |  |  | Clip domain serine protease homolog |
| AAEL005792 | CLIPE8 | 2.28 |  |  |  |  | 1.85 |  | Clip domain serine protease homolog |
| AAEL001233 | CLIPE9 |  |  |  | 1.34 |  |  | -0.98 | Clip domain serine protease homolog |
| AAEL014079 | serpin-1 | 1.11 | 0.91 |  |  |  | 1.00 |  | serine protease inhibitor, serpin |
| AAEL002699 | serpin-7 |  |  | -1.14 |  |  |  |  | serine protease inhibitor, serpin |
| AAEL002720 | serpin-20 | 0.81 |  | -1.32 |  |  | 0.98 |  | serine protease inhibitor, serpin |
| AAEL002730 | serpin-21 |  |  | -0.86 |  |  |  |  | serine protease inhibitor, serpin |
| AAEL002715 | serpin-22 |  |  |  | 1.46 |  |  |  | serine protease inhibitor, serpin |
| AAEL011777 | serpin-8 | 0.87 | 0.85 |  | 0.96 |  | 1.38 |  | serine protease inhibitor, serpin |
| AAEL008364 | serpin-9 |  | 0.98 |  |  |  | 1.28 |  | serine protease inhibitor, serpin |
| AAEL007765 | serpin-10A |  |  |  | -0.83 |  | 0.84 |  | serine protease inhibitor, serpin |
| AAEL007765 | serpin-10B |  |  |  | -0.83 |  | 0.84 |  | serine protease inhibitor, serpin |
| AAEL007765 | serpin-10C |  |  |  | -0.83 |  | 0.84 |  | serine protease inhibitor, serpin |
| AAEL007765 | serpin-10D |  |  |  | -0.83 |  | 0.84 |  | serine protease inhibitor, serpin |
| AAEL014078 | serpin-2 | 0.99 |  |  |  |  |  | -2.03 | serine protease inhibitor, serpin |
| AAEL014138 | serpin-16 |  | 0.85 |  |  |  | 1.97 |  | serine protease inhibitor, serpin |
| AAEL005665 | serpin-3 |  |  |  |  |  | 1.15 |  | serine protease inhibitor, serpin |
| AAEL013933 | serpin-4B |  | 0.96 |  |  |  | 1.30 |  | serine protease inhibitor, serpin |
| AAEL013936 | serpin-4A | -1.77 |  | -0.98 |  |  | 1.43 |  | serine protease inhibitor, serpin |
| AAEL013937 | serpin-4C |  | 0.87 |  |  |  | 1.12 |  | serine protease inhibitor, serpin |
| AAEL013934 | serpin-4D |  |  |  | -0.87 |  | 0.91 |  | serine protease inhibitor, serpin |
| AAEL014141 | serpin-5 |  | 0.95 |  |  |  | 2.00 |  | serine protease inhibitor, serpin |
| AAEL010769 | serpin-6 |  |  |  | 0.98 |  | 1.63 |  | serine protease inhibitor, serpin |
| AAEL003686 | serpin-11 | 0.88 |  | -0.85 |  |  |  |  | serine protease inhibitor, serpin |
| AAEL003653 | serpin-12 |  |  |  |  |  |  |  | serine protease inhibitor, serpin |
| AAEL002731 | serpin-14 |  |  |  | 1.43 |  |  |  | serine protease inhibitor, serpin |
| AAEL002704 | serpin-23 | 0.91 |  |  |  |  | -0.92 |  | serine protease inhibitor, serpin |
| AAEL006137 | serpin-19 |  | 0.93 |  | 1.20 |  |  |  | serine protease inhibitor, serpin |
| AAEL007420 | serpin-25 |  |  |  |  |  |  |  | serine protease inhibitor, serpin |
| AAEL003697 | serpin-17 |  |  | -1.00 |  |  | 1.40 |  | serine protease inhibitor, serpin |
| AAEL003182 | serpin-26 |  |  |  |  |  |  |  | serine protease inhibitor, serpin |
| AAEL013498 | PPO1 |  |  |  | -0.86 |  |  |  | prophenoloxidase |
| AAEL011764 | PPO10 |  |  | -0.99 |  |  | -0.84 |  | prophenoloxidase |
| AAEL013499 | PPO2 |  |  |  |  |  |  |  | prophenoloxidase |
| AAEL013499 | PPO2 |  |  | -0.88 |  |  | 1.33 |  | prophenoloxidase |
| AAEL011763 | PPO3 |  |  |  |  |  | -1.16 |  | prophenoloxidase |
| AAEL013501/006877 | PPO4 |  |  | -0.91 | 1.14 |  |  |  | prophenoloxidase |
| AAEL013492 | PPO5 | -0.81 |  |  |  |  |  |  | prophenoloxidase |
| AAEL014544 | PPO6 |  |  |  | 1.11 |  |  |  | prophenoloxidase |
| AAEL013496 | PPO8 |  |  |  |  |  |  |  | prophenoloxidase |
| AAEL014837 | PPO9 |  |  | -0.94 |  |  |  |  | prophenoloxidase |
| AAEL013493 | PPO7 |  |  |  |  |  |  |  | prophenoloxidase |

Log2 transformed expression ratio data is shown.
