# Supplementary material for: Transcriptome Analysis of Aedes aegypti Transgenic Mosquitoes with Altered Immunity
Source: PLoS Pathog. 2011 Nov 17;7(11):e1002394. doi: 10.1371/journal.ppat.1002394 (PMC3219725; doi:10.1371/journal.ppat.1002394)
Supplement: Table S6 — Gene repertoire induced in the Aedes aegypti midgut, 24 h after infection with P. gallinaceum . Data obtained by means of a full genome Agilent-based microarray analysis. Gene ID, gene name, functional group and log fold increase (decrease) are presented. Abbreviations are: IMM, immunity; R/S/M, redox, stress and mitochondrion; DIG, blood and sugar food digestive; C/S, cytoskeletal and structural; PROT, proteolysis; TRP, transport; R/T/T, replication, transcription, and translation; MET, metabolism; DIV, diverse functions; UNK, unknown functions. (DOCX) [file ppat.1002394.s011.docx]

Table S6. Gene repertoire induced in the *Aedes aegypti* midgut 24 h after infection with *P. gallinaceum*. Data obtained by means of a full genome Agilent-based microarray analysis. Gene ID, gene name, functional group and log fold increase (decrease) are presented. Abbreviations are: IMM, immunity; R/S/M, redox, stress and mitochondrion; DIG, blood and sugar food digestive; C/S, cytoskeletal and structural; PROT, proteolysis; TRP, transport; R/T/T, replication, transcription, and translation; MET, metabolism; DIV, diverse functions; UNK, unknown functions.

| GENE ID | Name | Func group | Logfold |
| --- | --- | --- | --- |
| AAEL001195 | Juvenile hormone-inducible protein, putative | DIV | 9.23 |
| AAEL013797 | Kinesin-like protein | DIV | 1.96 |
| AAEL011532 | hypothetical protein | UNK | 1.94 |
| AAEL015418 | neuropeptide y receptor (npy-r) (pr4 receptor) | DIV | 1.93 |
| AAEL005745 | neurokinin-3 receptor, putative | TRP | 1.87 |
| AAEL015257 | Probable palmitoyltransferase ZDHHC5 | PROT | 1.84 |
| AAEL000631 | Transcription factor glial cells missing | R/T/T | 1.82 |
| AAEL014962 | conserved hypothetical protein | UNK | 1.82 |
| AAEL005381 | Dissatisfaction (Dsf) | DIV | 1.8 |
| AAEL015289 | hypothetical protein | UNK | 1.8 |
| AAEL007454 | conserved hypothetical protein | UNK | 1.79 |
| AAEL000969 | hypothetical protein | UNK | 1.79 |
| AAEL002996 | dolichyl glycosyltransferase | DIV | 1.78 |
| AAEL009155 | conserved hypothetical protein | UNK | 1.75 |
| AAEL003339 | pita, isoform A | DIV | 1.73 |
| AAEL011845 | tryptophan/tyrosine permease | DIV | 1.73 |
| AAEL012582 | RrnaAD, ribosomal RNA adenine dimethylase, putative | DIV | 1.72 |
| AAEL006092 | tartan (LRR) | IMM | 1.72 |
| AAEL012570 | acid phosphatase SurE | DIV | 1.7 |
| AAEL011903 | zinc finger protein | DIV | 1.7 |
| AAEL002355 | BarH-like 1 homeobox protein | DIV | 1.7 |
| AAEL008690 | apterous | DIV | 1.7 |
| AAEL003312 | oxidoreductase | DIV | 1.67 |
| AAEL014526 | sideroflexin 1,2,3 | DIV | 1.66 |
| AAEL006719 | alpha-amylase | DIG | 1.66 |
| AAEL008937 | hypothetical protein | UNK | 1.65 |
| AAEL008338 | ion channel nompc | TRP | 1.64 |
| AAEL004116 | hypothetical protein | UNK | 1.64 |
| AAEL015467 | segmentation protein cap'n'collar | DIV | 1.63 |
| AAEL004174 | t-box transcription factor tbx6 | R/T/T | 1.63 |
| AAEL006057 | heterogeneous nuclear ribonucleoprotein | R/T/T | 1.63 |
| AAEL004625 | conserved hypothetical protein | UNK | 1.63 |
| AAEL000896 | conserved hypothetical protein | UNK | 1.61 |
| AAEL004140 | hypothetical protein | UNK | 1.61 |
| AAEL013866 | wdpeat protein | DIV | 1.6 |
| AAEL001349 | zinc finger protein | DIV | 1.6 |
| AAEL002625 | mucin-like peritrophin | C/S | 1.58 |
| AAEL008961 | brother of odd with entrails limited, isoform C | DIV | 1.58 |
| AAEL004112 | TPX2 | IMM | 1.58 |
| AAEL010851 | synaptotagmin-12 | DIV | 1.57 |
| AAEL013184 | open rectifier K[+] channel 1, isoform B | DIV | 1.57 |
| AAEL009322 | hypothetical protein | UNK | 1.57 |
| AAEL006154 | chitin binding domain 3 protein | C/S | 1.56 |
| AAEL002621 | blom7, putative | DIV | 1.56 |
| AAEL015522 | f-box and wd40 domain protein 7 | DIV | 1.56 |
| AAEL013747 | phosphodiesterase 6 | DIV | 1.55 |
| AAEL008028 | monocarboxylate transporter | TRP | 1.55 |
| AAEL008773 | laminin A chain, putative | C/S | 1.54 |
| AAEL004358 | moira, isoform B | DIV | 1.54 |
| AAEL007665 | AraC family transcription regulator | DIV | 1.52 |
| AAEL002206 | rap gtpase-activating protein | IMM | 1.52 |
| AAEL004168 | syntaxin | TRP | 1.52 |
| AAEL007441 | translocon-associated protein, gamma subunit | DIV | 1.51 |
| AAEL009382 | nucleolar protein 66 | DIV | 1.51 |
| AAEL011771 | hypothetical protein | UNK | 1.51 |
| AAEL010333 | probable ribosome biogenesis protein | DIV | 1.5 |
| AAEL006167 | runt | DIV | 1.5 |
| AAEL003364 | protein phosphatase 1-binding protein Bifocal | DIV | 1.5 |
| AAEL012480 | sodium/calcium exchanger | TRP | 1.5 |
| AAEL001692 | conserved hypothetical protein | UNK | 1.5 |
| AAEL008306 | mitogen activated protein kinase kinase kinase 5, mapkkk5, mekk5 | DIV | 1.49 |
| AAEL007833 | fatty acid desaturase | DIV | 1.49 |
| AAEL011265 | abc transporter | TRP | 1.49 |
| AAEL004344 | zinc finger protein | DIV | 1.48 |
| AAEL007744 | hypothetical protein | UNK | 1.48 |
| AAEL004149 | hypothetical protein | UNK | 1.48 |
| AAEL010272 | conserved hypothetical protein | UNK | 1.48 |
| AAEL009587 | organic cation transporter | DIV | 1.47 |
| AAEL011417 | synaptojanin | DIV | 1.47 |
| AAEL004741 | aquaporin transporter | TRP | 1.47 |
| AAEL002693 | venom allergen | MET | 1.46 |
| AAEL011679 | ion channel nompc | TRP | 1.46 |
| AAEL009863 | sodium/dicarboxylate cotransporter, putative | TRP | 1.46 |
| AAEL011064 | hypothetical protein | UNK | 1.46 |
| AAEL010488 | ets | DIV | 1.45 |
| AAEL008365 | conserved hypothetical protein | UNK | 1.45 |
| AAEL014131 | hypothetical protein | UNK | 1.44 |
| AAEL006997 | putative trypsin-like inhibitor protein | DIV | 1.43 |
| AAEL014727 | Osiris, putative | DIV | 1.43 |
| AAEL012234 | cytoplasmic dynein intermediate chain, (dhic) | DIV | 1.43 |
| AAEL001585 | predicted protein | DIV | 1.43 |
| AAEL008323 | conserved hypothetical protein | UNK | 1.42 |
| AAEL000856 | germ cell-less protein | DIV | 1.41 |
| AAEL013175 | connector enhancer of ksr | DIV | 1.41 |
| AAEL003632 | CLIPB39 | IMM | 1.41 |
| AAEL004911 | DEAD box ATP-dependent RNA helicase | R/T/T | 1.41 |
| AAEL006502 | conserved hypothetical protein | UNK | 1.4 |
| AAEL002545 | vacuolar H+-ATPase v0 sector accessory subunit | DIV | 1.39 |
| AAEL006855 | UDP-galactose transporter | TRP | 1.39 |
| AAEL005265 | conserved hypothetical protein | UNK | 1.39 |
| AAEL009473 | conserved hypothetical protein | UNK | 1.39 |
| AAEL010898 | conserved hypothetical protein | UNK | 1.39 |
| AAEL006567 | max binding protein, mnt | DIV | 1.37 |
| AAEL004354 | hypothetical protein | UNK | 1.37 |
| AAEL009305 | numb-associated kinase | DIV | 1.36 |
| AAEL011167 | cathepsin l | IMM | 1.36 |
| AAEL014041 | hypothetical protein | UNK | 1.36 |
| AAEL012754 | hypothetical protein | UNK | 1.36 |
| AAEL007437 | conserved hypothetical protein | UNK | 1.36 |
| AAEL008092 | hypothetical protein | UNK | 1.36 |
| AAEL003335 | ionotropic receptor 7c | DIV | 1.35 |
| AAEL008839 | zinc finger protein 32 | DIV | 1.35 |
| AAEL001034 | adenylate cyclase type vi | DIV | 1.35 |
| AAEL003372 | tankyrase | R/T/T | 1.35 |
| AAEL010636 | hypothetical protein | UNK | 1.35 |
| AAEL003064 | Sugar phosphate exchanger 2 | DIV | 1.34 |
| AAEL004346 | prosap | DIV | 1.34 |
| AAEL003399 | cytochrome P450 | R/S/M | 1.34 |
| AAEL000098 | RNA polymerase II largest subunit | R/T/T | 1.34 |
| AAEL004114 | UNC93A protein, putative | DIV | 1.33 |
| AAEL004426 | Syntaxin 18, putative | DIV | 1.33 |
| AAEL006101 | carboxylesterase | R/S/M | 1.33 |
| AAEL013169 | conserved hypothetical protein | UNK | 1.33 |
| AAEL006587 | thiamin pyrophosphokinase 1 | DIV | 1.31 |
| AAEL009360 | serine/threonine protein kinase | DIV | 1.31 |
| AAEL000662 | conserved hypothetical protein | UNK | 1.31 |
| AAEL005409 | regulator of g protein signaling | DIV | 1.3 |
| AAEL001843 | ski oncogene | R/T/T | 1.3 |
| AAEL011213 | conserved hypothetical protein | UNK | 1.3 |
| AAEL015416 | conserved hypothetical protein | UNK | 1.3 |
| AAEL012927 | hypothetical protein | UNK | 1.3 |
| AAEL012831 | bestrophin 2,3,4 | DIV | 1.29 |
| AAEL014056 | hypothetical protein | UNK | 1.28 |
| AAEL006062 | neuralized | DIV | 1.28 |
| AAEL000902 | sugar transporter | TRP | 1.28 |
| AAEL007867 | hypothetical protein | UNK | 1.28 |
| AAEL000700 | cadherin | C/S | 1.27 |
| AAEL011774 | sarcolemmal associated protein, putative | DIV | 1.27 |
| AAEL012566 | zinc finger protein Xfin-like | DIV | 1.27 |
| AAEL006854 | ML13 | IMM | 1.27 |
| AAEL008877 | conserved hypothetical protein | UNK | 1.27 |
| AAEL003226 | pupal cuticle protein 78E, putative | C/S | 1.25 |
| AAEL002012 | Phosphorylated adaptor for RNA export, putative | TRP | 1.25 |
| AAEL004447 | hypothetical protein | UNK | 1.25 |
| AAEL007746 | bves, isoform A | DIV | 1.24 |
| AAEL005012 | glutamate receptor delta-1 subunit precursor | DIV | 1.24 |
| AAEL007397 | ecdysone-induced protein 75b | DIV | 1.24 |
| AAEL012586 | conserved hypothetical protein | UNK | 1.24 |
| AAEL008432 | high-affinity choline transporter | DIV | 1.23 |
| AAEL010819 | Vacuolar ATP synthase subunit H | TRP | 1.23 |
| AAEL004032 | acetylcholine receptor protein alpha 1, 2, 3, 4 invertebrate | TRP | 1.23 |
| AAEL008612 | hypothetical protein | UNK | 1.23 |
| AAEL011250 | vulcan, isoform B | DIV | 1.22 |
| AAEL012721 | conserved hypothetical protein | UNK | 1.22 |
| AAEL006337 | conserved hypothetical protein | UNK | 1.22 |
| AAEL007053 | receptor protein kinase, putative | DIV | 1.21 |
| AAEL010354 | homeobox protein nk-2 | R/T/T | 1.21 |
| AAEL008874 | hypothetical protein | UNK | 1.21 |
| AAEL012078 | Cation transport regulator-like protein 2 | DIV | 1.2 |
| AAEL008686 | vulcan, isoform B | DIV | 1.2 |
| AAEL004691 | ring finger | DIV | 1.2 |
| AAEL015190 | frizzled, isoform B | DIV | 1.19 |
| AAEL014244 | glucosyl/glucuronosyl transferases | MET | 1.19 |
| AAEL014255 | aquaporin, putative | TRP | 1.19 |
| AAEL005784 | hypothetical protein | UNK | 1.19 |
| AAEL013178 | hypothetical protein | UNK | 1.19 |
| AAEL009508 | zinc finger protein | DIV | 1.18 |
| AAEL007199 | hypothetical protein | DIV | 1.18 |
| AAEL002951 | forkhead protein/ forkhead protein domain | R/T/T | 1.18 |
| AAEL003209 | lachesin | DIV | 1.17 |
| AAEL013965 | RING finger protein 126-B | DIV | 1.17 |
| AAEL005945 | beta adrenergic receptor | DIV | 1.17 |
| AAEL005578 | conserved hypothetical protein | UNK | 1.17 |
| AAEL006534 | protocadherin | C/S | 1.16 |
| AAEL005570 | replication initiator 1, isoform CRA_a | DIV | 1.16 |
| AAEL008457 | similar to protein tyrosine phosphatase, receptor type | DIG | 1.16 |
| AAEL006555 | hypothetical protein | UNK | 1.16 |
| AAEL013908 | conserved hypothetical protein | UNK | 1.16 |
| AAEL011505 | integral membrane pore glycoprotein gp210, putative | TRP | 1.15 |
| AAEL011138 | hypothetical protein | UNK | 1.15 |
| AAEL015340 | hypothetical protein | UNK | 1.15 |
| AAEL013180 | hypothetical protein | UNK | 1.15 |
| AAEL007123 | receptor tyrosine kinase | DIV | 1.14 |
| AAEL005293 | GALE8A | IMM | 1.14 |
| AAEL014490 | conserved hypothetical protein | UNK | 1.14 |
| AAEL005179 | hypothetical protein | UNK | 1.14 |
| AAEL014047 | hypothetical protein | UNK | 1.14 |
| AAEL002095 | conserved hypothetical protein | UNK | 1.14 |
| AAEL002859 | anaphase-promoting complex subunit 2 | DIV | 1.13 |
| AAEL014233 | pickpocket | TRP | 1.13 |
| AAEL012367 | hypothetical protein | UNK | 1.13 |
| AAEL012809 | peptidylprolyl isomerase | MET | 1.12 |
| AAEL014344 | adam (a disintegrin and metalloprotease | MET | 1.12 |
| AAEL009905 | DNA polymerase subunit alpha B | R/T/T | 1.12 |
| AAEL003433 | copper-transporting atpase 1, 2 (copper pump 1, 2) | TRP | 1.12 |
| AAEL012058 | hypothetical protein | UNK | 1.12 |
| AAEL005669 | conserved hypothetical protein | UNK | 1.12 |
| AAEL004912 | hypothetical protein | UNK | 1.12 |
| AAEL001930 | pra1 protein | DIV | 1.11 |
| AAEL010916 | tyrosine-protein phosphatase, nonceptor type 23, putative | DIV | 1.11 |
| AAEL004208 | hypothetical protein | UNK | 1.11 |
| AAEL014194 | conserved hypothetical protein | UNK | 1.11 |
| AAEL010947 | conserved hypothetical protein | UNK | 1.11 |
| AAEL007020 | hypothetical protein | UNK | 1.1 |
| AAEL011779 | zinc finger protein and BTB domain-containin | DIV | 1.1 |
| AAEL000781 | ubiquitin ligase sia-1, putative | DIV | 1.1 |
| AAEL014635 | engrailed | DIV | 1.1 |
| AAEL003631 | CLIPB41 | IMM | 1.1 |
| AAEL006146 | hypothetical protein | UNK | 1.1 |
| AAEL006757 | hypothetical protein | UNK | 1.1 |
| AAEL009577 | obstractor B | C/S | 1.09 |
| AAEL002094 | Zinc finger protein 516 | DIV | 1.09 |
| AAEL007296 | Fatty acyl-CoA reductase 1 | DIV | 1.09 |
| AAEL008042 | vegetable | DIV | 1.08 |
| AAEL011079 | galactose-specific C-type lectin, putative | IMM | 1.08 |
| AAEL013506 | FBN29 | IMM | 1.08 |
| AAEL004947 | elongase, putative | MET | 1.08 |
| AAEL013161 | heat shock protein, putative | R/S/M | 1.08 |
| AAEL010557 | paraxis protein | R/T/T | 1.08 |
| AAEL002460 | conserved hypothetical protein | UNK | 1.08 |
| AAEL001149 | Serine-enriched protein | DIV | 1.07 |
| AAEL011652 | Putative ribonuclease ZC3H12C | DIV | 1.07 |
| AAEL010869 | hypothetical protein | UNK | 1.07 |
| AAEL006904 | stromal membrane-associated protein | DIV | 1.06 |
| AAEL011621 | serine protease | IMM | 1.06 |
| AAEL003439 | caspase-s18 | IMM | 1.06 |
| AAEL007213 | delta(9)-desaturase, putative | MET | 1.06 |
| AAEL004273 | short-chain dehydrogenase | R/S/M | 1.06 |
| AAEL010226 | daughterless | R/S/M | 1.06 |
| AAEL005850 | expressed protein (HR4) | DIV | 1.05 |
| AAEL012135 | GALE2 | IMM | 1.05 |
| AAEL006386 | mitochondrial 39S ribosomal protein L39 | R/S/M | 1.05 |
| AAEL009565 | conserved hypothetical protein | UNK | 1.05 |
| AAEL007389 | rhodopsin | DIV | 1.04 |
| AAEL004167 | zinc finger protein | DIV | 1.04 |
| AAEL003745 | similar to RNA polymerase II tran | DIV | 1.04 |
| AAEL015091 | cys-loop ligand-gated ion channel subunit | DIV | 1.04 |
| AAEL003098 | glucosyl/glucuronosyl transferases | MET | 1.04 |
| AAEL012227 | conserved hypothetical protein | UNK | 1.04 |
| AAEL013488 | glucose inhibited division protein a | DIV | 1.03 |
| AAEL005296 | fatty acyl-CoA reductase 2 | DIV | 1.03 |
| AAEL002453 | zinc finger protein | DIV | 1.03 |
| AAEL010360 | nucleotide binding protein 2 (nbp 2) | DIV | 1.03 |
| AAEL005512 | speckle-type poz protein | DIV | 1.03 |
| AAEL014710 | alpha-amylase | DIG | 1.03 |
| AAEL012958 | leucine-rich repeat (LRR) | IMM | 1.03 |
| AAEL009254 | aaa atpase | TRP | 1.03 |
| AAEL011699 | yellow-e | IMM | 1.02 |
| AAEL014246 | glucosyl/glucuronosyl transferases | MET | 1.02 |
| AAEL007116 | peritrophic matrix protein 14 | C/S | 1.01 |
| AAEL009139 | poor gastrulation, isoform B | DIV | 1.01 |
| AAEL011000 | pickpocket | TRP | 1.01 |
| AAEL012595 | lipoprotein NlpD | DIV | 1 |
| AAEL009021 | peptidylprolyl isomerase | MET | 1 |
| AAEL004222 | voltage-dependent p/q type calcium channel | TRP | 1 |
| AAEL011818 | hypothetical protein | UNK | 1 |
| AAEL004801 | hypothetical protein | UNK | 1 |
| AAEL009032 | phosphatidylinositol transfer protein SEC14 | DIV | 0.99 |
| AAEL010862 | BIP2 protein | DIV | 0.99 |
| AAEL012815 | hypothetical protein | UNK | 0.99 |
| AAEL002821 | zinc finger protein 709 | DIV | 0.98 |
| AAEL012178 | dappled | DIV | 0.98 |
| AAEL005994 | similar to SIFamide receptor | DIV | 0.98 |
| AAEL000508 | fbn15 | IMM | 0.98 |
| AAEL006001 | conserved hypothetical protein | UNK | 0.98 |
| AAEL008910 | hypothetical protein | UNK | 0.98 |
| AAEL003316 | hypothetical protein | UNK | 0.98 |
| AAEL000745 | hypothetical protein | UNK | 0.98 |
| AAEL004546 | coatomer beta subunit | DIV | 0.97 |
| AAEL002615 | leucinech transmembrane protein | IMM | 0.97 |
| AAEL001456 | division cycle and apoptosis regulator protein | R/T/T | 0.97 |
| AAEL005575 | transient receptor potential channel 4, putative | TRP | 0.97 |
| AAEL005379 | conserved hypothetical protein | UNK | 0.97 |
| AAEL007743 | hypothetical protein | UNK | 0.97 |
| AAEL001065 | conserved hypothetical protein | UNK | 0.97 |
| AAEL003014 | zinc finger protein 395-like | DIV | 0.96 |
| AAEL005814 | Protogenin | DIV | 0.96 |
| AAEL015227 | ionotropic receptor 7c | DIV | 0.96 |
| AAEL008492 | conserved hypothetical protein | UNK | 0.96 |
| AAEL015259 | hypothetical protein | UNK | 0.96 |
| AAEL004666 | focal adhesion kinase | C/S | 0.95 |
| AAEL003080 | Btk family kinase at 29A, isoform B | DIV | 0.95 |
| AAEL007345 | cyclic-nucleotide-gated cation channel | TRP | 0.95 |
| AAEL000708 | hiv-1 rev binding protein | DIV | 0.94 |
| AAEL009709 | Dynein heavy chain 1, axonemal | DIV | 0.94 |
| AAEL012953 | serine protease, putative | PROT | 0.94 |
| AAEL006072 | hypothetical protein | UNK | 0.94 |
| AAEL005907 | carboxypeptidase N subunit 2 | DIV | 0.93 |
| AAEL014496 | NADH-cytochrome b5 reductase | DIV | 0.93 |
| AAEL006355 | SCRC1 | IMM | 0.93 |
| AAEL012813 | sphingolipid delta 4 desaturase/c-4 hydroxylase protein des2 | MET | 0.93 |
| AAEL000191 | conserved hypothetical protein | UNK | 0.93 |
| AAEL011300 | conserved hypothetical protein | UNK | 0.93 |
| AAEL010724 | conserved hypothetical protein | UNK | 0.93 |
| AAEL005021 | conserved hypothetical protein | UNK | 0.93 |
| AAEL012221 | Sodium channel protein 60E | DIV | 0.92 |
| AAEL014132 | thioredoxin binding protein tBP-2/VDUP1 | DIV | 0.92 |
| AAEL005275 | hypothetical protein | UNK | 0.92 |
| AAEL003934 | conserved hypothetical protein | UNK | 0.92 |
| AAEL012088 | cuticular protein RR-1 motif 54 | C/S | 0.91 |
| AAEL002080 | septin interacting protein, putative | DIV | 0.91 |
| AAEL007793 | alkyldihydroxyacetonephosphate synthase | DIV | 0.91 |
| AAEL002136 | zinc finger protein | DIV | 0.91 |
| AAEL007924 | neuropeptide f receptor 76f | DIV | 0.91 |
| AAEL008626 | polyprotein | DIV | 0.91 |
| AAEL012890 | fibrinogen-binding protein | DIV | 0.91 |
| AAEL013301 | Dorsal isoform 1-A | IMM | 0.91 |
| AAEL000320 | cytochrome P450 | R/S/M | 0.91 |
| AAEL006493 | hypothetical protein | UNK | 0.91 |
| AAEL007748 | SCRB10 | IMM | 0.9 |
| AAEL011690 | conserved hypothetical protein | UNK | 0.9 |
| AAEL010846 | hypothetical protein | UNK | 0.9 |
| AAEL008460 | hypothetical protein | UNK | 0.9 |
| AAEL000584 | sex-determining region y protein, sry | DIV | 0.89 |
| AAEL011217 | PQ loop repeat-containing protein 3 | DIV | 0.89 |
| AAEL014408 | m-phase inducer phosphatase(cdc25) | DIV | 0.89 |
| AAEL013299 | serine protease, putative | IMM | 0.89 |
| AAEL002230 | chromodomain helicase DNA binding protein | R/T/T | 0.89 |
| AAEL007015 | conserved hypothetical protein | UNK | 0.89 |
| AAEL014622 | Zinc finger CCHC domain-containing protein 2 | DIV | 0.88 |
| AAEL002893 | short-chain dehydrogenase | R/S/M | 0.88 |
| AAEL014162 | potassium channel kcnq, putative | TRP | 0.88 |
| AAEL004583 | conserved hypothetical protein | UNK | 0.88 |
| AAEL012910 | Neurogenic locus notch-like protein protein 1 | DIV | 0.87 |
| AAEL006438 | dolichyl glycosyltransferase | DIV | 0.87 |
| AAEL014688 | ionotropic receptor 7b | DIV | 0.87 |
| AAEL011165 | conserved hypothetical protein | UNK | 0.87 |
| AAEL000036 | hypothetical protein | UNK | 0.87 |
| AAEL005462 | hypothetical protein | UNK | 0.87 |
| AAEL007282 | syntaxin binding protein-1,2,3 | DIV | 0.86 |
| AAEL015037 | g-protein-linked acetylcholine receptor gar-2a | DIV | 0.86 |
| AAEL009535 | adenosine monophosphate deaminase | DIV | 0.86 |
| AAEL002275 | kv channel-interacting protein 1 (KChIP1) , putative | DIV | 0.86 |
| AAEL008374 | E3 ubiquitin-protein ligase nedd-4 | MET | 0.86 |
| AAEL009294 | phosphatidylinositol 4-kinase | R/T/T | 0.86 |
| AAEL005057 | Nlp protein | R/T/T | 0.86 |
| AAEL007779 | delilah | R/T/T | 0.86 |
| AAEL007945 | eukaryotic translation initiation factor 3 subunit | R/T/T | 0.86 |
| AAEL005723 | conserved hypothetical protein | UNK | 0.86 |
| AAEL008495 | expanded | DIV | 0.85 |
| AAEL010489 | hypothetical protein | UNK | 0.85 |
| AAEL001157 | light protein | DIV | 0.84 |
| AAEL003902 | d(1a,b) dopamine receptor | DIV | 0.84 |
| AAEL004948 | serine protease | IMM | 0.84 |
| AAEL007363 | leucinech transmembrane protein | IMM | 0.84 |
| AAEL001555 | conserved hypothetical protein | UNK | 0.84 |
| AAEL015634 | conserved hypothetical protein | UNK | 0.84 |
| AAEL001884 | TPA_inf: venus kinase receptor | DIV | 0.83 |
| AAEL008194 | protein phosphatase 2a, regulatory subunit | DIV | 0.83 |
| AAEL009442 | Jun dimerization protein 2 | DIV | 0.83 |
| AAEL003844 | GALE5 | IMM | 0.83 |
| AAEL000384 | vesicular acetylcholine transporter | TRP | 0.83 |
| AAEL008449 | conserved hypothetical protein | UNK | 0.83 |
| AAEL003200 | hypothetical protein | UNK | 0.83 |
| AAEL007408 | MIP09710p | DIV | 0.82 |
| AAEL002199 | FR47-like protein | DIV | 0.82 |
| AAEL012976 | type 11 methyltransferase | DIV | 0.82 |
| AAEL003643 | AMP dependent ligase | MET | 0.82 |
| AAEL002644 | conserved hypothetical protein | UNK | 0.82 |
| AAEL005156 | hypothetical protein | UNK | 0.82 |
| AAEL004255 | zinc finger protein | DIV | 0.81 |
| AAEL010889 | Phosphatidylinositol N-acetylglucosaminyltransferase | DIV | 0.81 |
| AAEL013047 | sphingolipid delta 4 desaturase/c-4 hydroxylase protein des2 | MET | 0.81 |
| AAEL013317 | hypothetical protein | UNK | 0.81 |
| AAEL014998 | Large proline-rich protein BAT3 | DIV | 0.8 |
| AAEL011572 | Oct1, putative | DIV | 0.8 |
| AAEL011050 | Activating signal cointegrator 1 complex subunit | DIV | -0.8 |
| AAEL012685 | Juvenile hormone-inducible protein | DIV | -0.8 |
| AAEL003647 | g-protein coupled receptor | DIV | -0.8 |
| AAEL011009 | FBN13 | IMM | -0.8 |
| AAEL006769 | tryptophanyl-tRNA synthetase | MET | -0.8 |
| AAEL002783 | mitochondrial ribosomal protein, L37, putative | R/S/M | -0.8 |
| AAEL002973 | zinc finger protein | DIV | -0.81 |
| AAEL002691 | Transport and Golgi organization protein 1 | DIV | -0.81 |
| AAEL004267 | parkin (ubiquitin E3 ligase prkn) | DIV | -0.81 |
| AAEL014231 | Male-specific RNA 98Cb | DIV | -0.81 |
| AAEL011432 | plexin b | DIV | -0.81 |
| AAEL011994 | Carbohydrate sulfotransferase 5 | DIV | -0.81 |
| AAEL003911 | DnaJ-like protein subfamily C member 14 | DIV | -0.81 |
| AAEL011716 | tartan (LRR) | IMM | -0.81 |
| AAEL001657 | DEAD box ATP-dependent RNA helicase | R/T/T | -0.81 |
| AAEL012423 | conserved hypothetical protein | UNK | -0.81 |
| AAEL006890 | conserved hypothetical protein | UNK | -0.81 |
| AAEL010445 | hypothetical protein | UNK | -0.81 |
| AAEL008717 | hypothetical protein | UNK | -0.81 |
| AAEL005439 | mical | DIV | -0.82 |
| AAEL007646 | Band4.1 inhibitor LRP interactor, isoform B | DIV | -0.82 |
| AAEL014529 | Ran binding protein 3 | DIV | -0.82 |
| AAEL004593 | zinc finger protein 564 | DIV | -0.82 |
| AAEL013473 | camp-specific 3,5-cyclic phosphodiesterase | DIV | -0.82 |
| AAEL013171 | HPX2 | IMM | -0.82 |
| AAEL013213 | enhancer of zeste, ezh | MET | -0.82 |
| AAEL014582 | step ii splicing factor slu7 | MET | -0.82 |
| AAEL003524 | monocarboxylate transporter | TRP | -0.82 |
| AAEL003472 | conserved hypothetical protein | UNK | -0.82 |
| AAEL002962 | conserved hypothetical protein | UNK | -0.82 |
| AAEL011844 | 5-hydroxytryptamine receptor 1 | DIV | -0.83 |
| AAEL007374 | yellow protein precursor, putative | DIV | -0.83 |
| AAEL000084 | elongin b | DIV | -0.83 |
| AAEL011485 | sumo ligase | DIV | -0.83 |
| AAEL003528 | cdc73 domain protein | DIV | -0.83 |
| AAEL007688 | serine/threonine protein kinase, putative | DIV | -0.83 |
| AAEL009909 | cln3/battenin | DIV | -0.83 |
| AAEL011596 | mitotic checkpoint serine/threonine-protein kinase bub1 and bubr1 | DIV | -0.83 |
| AAEL006949 | SOCS 16D2 | IMM | -0.83 |
| AAEL007587 | CLIP | IMM | -0.83 |
| AAEL005732 | acyl-coa dehydrogenase | MET | -0.83 |
| AAEL002067 | cytochrome P450 | R/S/M | -0.83 |
| AAEL006721 | 2-oxoglutarate dehydrogenase | R/S/M | -0.83 |
| AAEL006574 | nuclear transcription factor, x-box binding 1 (nfx1) | TRP | -0.83 |
| AAEL008113 | hypothetical protein | UNK | -0.83 |
| AAEL003264 | conserved hypothetical protein | UNK | -0.83 |
| AAEL001180 | hypothetical protein | UNK | -0.83 |
| AAEL001359 | hypothetical protein | UNK | -0.83 |
| AAEL010193 | conserved hypothetical protein | UNK | -0.83 |
| AAEL003081 | conserved hypothetical protein | UNK | -0.83 |
| AAEL003420 | conserved hypothetical protein | UNK | -0.83 |
| AAEL015503 | adult cuticle protein, putative | C/S | -0.84 |
| AAEL001344 | pairle protein odd-paired | DIV | -0.84 |
| AAEL004602 | neurogenic differentiation factor, putative | DIV | -0.84 |
| AAEL013830 | bmp-induced factor | DIV | -0.84 |
| AAEL003990 | myeloid leukemia factor, putative | DIV | -0.84 |
| AAEL010865 | defective proboscis extension response | DIV | -0.84 |
| AAEL013105 | receptor protein tyrosine phosphatase | DIV | -0.84 |
| AAEL002629 | serine protease | IMM | -0.84 |
| AAEL004401 | HPX7 | IMM | -0.84 |
| AAEL004942 | helicase | R/T/T | -0.84 |
| AAEL008814 | 5'-3' exoribonuclease, putative | R/T/T | -0.84 |
| AAEL013721 | RNA binding protein, putative | R/T/T | -0.84 |
| AAEL004025 | glucose dehydrogenase | TRP | -0.84 |
| AAEL013275 | importin beta-1 | TRP | -0.84 |
| AAEL008361 | hypothetical protein | UNK | -0.84 |
| AAEL003812 | hypothetical protein | UNK | -0.84 |
| AAEL000322 | hypothetical protein | UNK | -0.84 |
| AAEL009456 | hypothetical protein | UNK | -0.84 |
| AAEL005283 | EsV-1-166 (conserved hypothetical) | C/S | -0.85 |
| AAEL001554 | phd finger protein | DIV | -0.85 |
| AAEL000564 | myotubularin | DIV | -0.85 |
| AAEL012747 | RhoGAP71E, isoform B | DIV | -0.85 |
| AAEL010169 | LOW QUALITY PROTEIN: ankyrin repeats | DIV | -0.85 |
| AAEL011178 | posterior sex combs protein | DIV | -0.85 |
| AAEL008898 | sulfotransferase (sult) | DIV | -0.85 |
| AAEL010392 | putative farnesoic acid O-methyl transferase | DIV | -0.85 |
| AAEL013079 | ankyrin repeat domain-containing protein | DIV | -0.85 |
| AAEL003686 | serpin-11 | IMM | -0.85 |
| AAEL011619 | galactose-specific C-type lectin, putative | IMM | -0.85 |
| AAEL004724 | carboxylesterase | R/S/M | -0.85 |
| AAEL001428 | transcription factor Hairy, putative | R/T/T | -0.85 |
| AAEL011039 | conserved hypothetical protein | UNK | -0.85 |
| AAEL009290 | hypothetical protein | UNK | -0.85 |
| AAEL003442 | conserved hypothetical protein | UNK | -0.85 |
| AAEL014950 | conserved hypothetical protein | UNK | -0.85 |
| AAEL010934 | hypothetical protein | UNK | -0.85 |
| AAEL009803 | phaF protein, putative | C/S | -0.86 |
| AAEL004047 | regulator of presynaptic morphology protein | DIV | -0.86 |
| AAEL008647 | no-mechanoreceptor potential a | DIV | -0.86 |
| AAEL015442 | zinc finger protein 25 | DIV | -0.86 |
| AAEL000640 | alanine-glyoxylate aminotransferase | DIV | -0.86 |
| AAEL008533 | B-cell lymphoma/leukaemia 11A extra long for | DIV | -0.86 |
| AAEL004823 | MnSOD1 | IMM | -0.86 |
| AAEL009423 | SCRBQ2 | IMM | -0.86 |
| AAEL002730 | serpin-21 | IMM | -0.86 |
| AAEL012400 | hydroxybutyrate dehydrogenase | MET | -0.86 |
| AAEL005975 | transcription initiation factor | R/T/T | -0.86 |
| AAEL012981 | sugar transporter | TRP | -0.86 |
| AAEL012222 | conserved hypothetical protein | UNK | -0.86 |
| AAEL002207 | conserved hypothetical protein | UNK | -0.86 |
| AAEL003811 | hypothetical protein | UNK | -0.86 |
| AAEL008796 | hypothetical protein | UNK | -0.86 |
| AAEL011124 | phd finger protein | UNK | -0.86 |
| AAEL006569 | bat5 hla-b-associated transcript | DIV | -0.87 |
| AAEL008550 | zinc finger protein, putative | DIV | -0.87 |
| AAEL014089 | ionotropic glutamate receptor-invertebrate | DIV | -0.87 |
| AAEL010579 | lipid storage droplets surface binding protein | DIV | -0.87 |
| AAEL004820 | zinc finger protein | DIV | -0.87 |
| AAEL012965 | wd and tetratricopeptide repeat protein | DIV | -0.87 |
| AAEL008535 | zinc finger protein, putative | DIV | -0.87 |
| AAEL011941 | oxidase/peroxidase | IMM | -0.87 |
| AAEL001121 | n-acetylgalactosaminyltransferase | MET | -0.87 |
| AAEL008322 | frizzled | PROT | -0.87 |
| AAEL009017 | glutathione-s-transferase theta, gst | R/S/M | -0.87 |
| AAEL000909 | histone H1, putative | R/T/T | -0.87 |
| AAEL002461 | RNA-binding protein, putative | R/T/T | -0.87 |
| AAEL001355 | conserved hypothetical protein | UNK | -0.87 |
| AAEL005863 | monocarboxylate transporter | DIV | -0.88 |
| AAEL002216 | 5-amp-activated protein kinase, beta subunit | DIV | -0.88 |
| AAEL011263 | phosphatidylethanolamine-binding protein | DIV | -0.88 |
| AAEL006894 | hypothetical protein | DIV | -0.88 |
| AAEL006901 | roundabout | DIV | -0.88 |
| AAEL010723 | elongin A, isoform B | DIV | -0.88 |
| AAEL010276 | aminomethyltransferase | DIV | -0.88 |
| AAEL002739 | zinc finger protein | DIV | -0.88 |
| AAEL005921 | d-lactate dehydrognease 2, putative | DIV | -0.88 |
| AAEL003108 | protein-tyrosine phosphatase, nonceptor type nt5 | DIV | -0.88 |
| AAEL013499 | PPO2 | IMM | -0.88 |
| AAEL011407 | type II transmembrane receptor OtB7, putative | IMM | -0.88 |
| AAEL006958 | cell adhesion molecule | IMM | -0.88 |
| AAEL010083 | IMD | IMM | -0.88 |
| AAEL007412 | sphingomyelin phosphodiesterase | MET | -0.88 |
| AAEL010814 | isocitrate dehydrogenase | MET | -0.88 |
| AAEL005564 | ubiquitin specific protease 52 / poly(a) ribonuclease subunit pan2 | PROT | -0.88 |
| AAEL001540 | ubiquitin specific protease | PROT | -0.88 |
| AAEL006514 | sodium-dependent phosphate transporter | TRP | -0.88 |
| AAEL015457 | conserved hypothetical protein | UNK | -0.88 |
| AAEL011620 | conserved hypothetical protein | UNK | -0.88 |
| AAEL004628 | hypothetical protein | UNK | -0.88 |
| AAEL006861 | hypothetical protein | UNK | -0.88 |
| AAEL008703 | scaffold attachment factor B, putative | DIV | -0.89 |
| AAEL013909 | pentapeptide repeat-containing protein | DIV | -0.89 |
| AAEL013040 | coagulation factor IX | DIV | -0.89 |
| AAEL014537 | maltose phosphorylase | DIV | -0.89 |
| AAEL008710 | short chain dehydrogenase | DIV | -0.89 |
| AAEL001274 | glycine-rich protein-like | DIV | -0.89 |
| AAEL012510 | NFkappaB essential modulator, putative | DIV | -0.89 |
| AAEL014356 | CTL | IMM | -0.89 |
| AAEL014667 | brain chitinase and chia | MET | -0.89 |
| AAEL014903 | 40S ribosomal protein S24 | R/T/T | -0.89 |
| AAEL015319 | hypothetical protein | UNK | -0.89 |
| AAEL012307 | conserved hypothetical protein | UNK | -0.89 |
| AAEL001723 | conserved hypothetical protein | UNK | -0.89 |
| AAEL008059 | conserved hypothetical protein | UNK | -0.89 |
| AAEL000061 | hypothetical protein | UNK | -0.89 |
| AAEL003630 | conserved hypothetical protein | UNK | -0.89 |
| AAEL003787 | TRAF-interacting protein | DIV | -0.9 |
| AAEL001475 | fibroblast growth factor receptor substrate 3 | DIV | -0.9 |
| AAEL014173 | Fps oncogene analog, isoform B | DIV | -0.9 |
| AAEL001721 | shroom, isoform G | DIV | -0.9 |
| AAEL012629 | deoxyuridine 5'-triphosphate nucleotidohydrolase | MET | -0.9 |
| AAEL014718 | shoc2 | R/S/M | -0.9 |
| AAEL008661 | AMP-activated protein kinase, gamma regulatory subunit | R/S/M | -0.9 |
| AAEL008397 | glutathione peroxidase | R/S/M | -0.9 |
| AAEL010293 | hypothetical protein | UNK | -0.9 |
| AAEL006060 | hypothetical protein | UNK | -0.9 |
| AAEL002659 | hypothetical protein | UNK | -0.9 |
| AAEL006408 | short D7 protein | CSR | -0.91 |
| AAEL011575 | Cysteine and histidine-rich protein 1-like | DIV | -0.91 |
| AAEL010053 | prolonged depolarization afterpotential (PDA) | DIV | -0.91 |
| AAEL009725 | ZNF343 protein | DIV | -0.91 |
| AAEL003175 | forkhead box protein (AaegFOXA) | DIV | -0.91 |
| AAEL003356 | adh transcription factor 1 | DIV | -0.91 |
| AAEL003938 | mRNA cleavage stimulating factor, 50kD-subunit, putative | DIV | -0.91 |
| AAEL004891 | zinc finger protein Ci-ZF(U1like)-9 | DIV | -0.91 |
| AAEL006722 | eclosion hormone, putative | DIV | -0.91 |
| AAEL000221 | mediator complex, subunit, putative | DIV | -0.91 |
| AAEL010737 | aromatic amino acid decarboxylase | IMM | -0.91 |
| AAEL003243 | serine protease | IMM | -0.91 |
| AAEL013501 | PPO4 | IMM | -0.91 |
| AAEL004574 | conserved hypothetical protein | UNK | -0.91 |
| AAEL007528 | conserved hypothetical protein | UNK | -0.91 |
| AAEL010357 | hypothetical protein | UNK | -0.91 |
| AAEL008068 | conserved hypothetical protein | UNK | -0.91 |
| AAEL008719 | Sm protein G, putative | DIV | -0.92 |
| AAEL009817 | PP2C-like domain-containing protein | DIV | -0.92 |
| AAEL000481 | rab3 interacting molecule | DIV | -0.92 |
| AAEL000866 | beta-lactamase | DIV | -0.92 |
| AAEL003205 | zinc finger protein 319 | DIV | -0.92 |
| AAEL005849 | synaptic vesicle protein | DIV | -0.92 |
| AAEL005176 | transcriptional repressor scratch, putative | DIV | -0.92 |
| AAEL004505 | serine collagenase 1 precursor, putative | IMM | -0.92 |
| AAEL013417 | FBN24 | IMM | -0.92 |
| AAEL012430 | AMP dependent ligase | MET | -0.92 |
| AAEL006695 | oligopeptide ABC transporter, periplasmic oligopeptide-binding protein transporter, periplasmic oligopeptide-binding protein [Borrelia garinii PBi] | PROT | -0.92 |
| AAEL001427 | short-chain dehydrogenase | R/S/M | -0.92 |
| AAEL004784 | 3-2trans-enoyl-CoA isomerase, putative | R/S/M | -0.92 |
| AAEL004036 | glucose dehydrogenase | TRP | -0.92 |
| AAEL014174 | hypothetical protein | UNK | -0.92 |
| AAEL011930 | conserved hypothetical protein | UNK | -0.92 |
| AAEL004617 | conserved hypothetical protein | UNK | -0.92 |
| AAEL009331 | general transcription factor 3C polypeptide | DIV | -0.93 |
| AAEL006525 | kelch repeat protein | DIV | -0.93 |
| AAEL002955 | osiris 20 | DIV | -0.93 |
| AAEL010576 | modifier of mdg4 | DIV | -0.93 |
| AAEL001014 | vacuolar protein sorting-associated | DIV | -0.93 |
| AAEL000581 | 5-oxoprolinase | DIV | -0.93 |
| AAEL005988 | LYSC6 | IMM | -0.93 |
| AAEL009953 | Niemann-Pick Type C-2, putative | IMM | -0.93 |
| AAEL011734 | leucinech transmembrane protein | IMM | -0.93 |
| AAEL003857 | DEFD | IMM | -0.93 |
| AAEL000760 | serine protease | IMM | -0.93 |
| AAEL007593 | lumbrokinase-3(1) precursor, putative | MET | -0.93 |
| AAEL009288 | mitochondrial ribosomal protein, L48, putative | R/S/M | -0.93 |
| AAEL011462 | transcription initiation factor IIE beta subunit | R/T/T | -0.93 |
| AAEL004259 | RNA-binding protein lin-28 | R/T/T | -0.93 |
| AAEL013151 | transcription factor IIB, putative | R/T/T | -0.93 |
| AAEL003718 | glucose transporter | TRP | -0.93 |
| AAEL002803 | conserved hypothetical protein | UNK | -0.93 |
| AAEL007648 | conserved hypothetical protein | UNK | -0.93 |
| AAEL005111 | hypothetical protein | UNK | -0.93 |
| AAEL012793 | conserved hypothetical protein | UNK | -0.93 |
| AAEL008312 | intestinal mucin | C/S | -0.94 |
| AAEL010927 | actin binding | C/S | -0.94 |
| AAEL000715 | zinc finger protein | DIV | -0.94 |
| AAEL000643 | zinc finger protein | DIV | -0.94 |
| AAEL015392 | Ubiquinone biosynthesis monooxygenase COQ6 | DIV | -0.94 |
| AAEL007165 | paramyosin | DIV | -0.94 |
| AAEL010812 | zinc finger protein | DIV | -0.94 |
| AAEL014158 | Putative ATP-dependent RNA helicase DHX35 | DIV | -0.94 |
| AAEL003153 | zinc finger protein 25 | DIV | -0.94 |
| AAEL010439 | zinc finger protein 436 | DIV | -0.94 |
| AAEL014837 | PPO9 | IMM | -0.94 |
| AAEL006985 | dopachrome-conversion enzyme (DCE), putative | IMM | -0.94 |
| AAEL007039 | PGRPS5 | IMM | -0.94 |
| AAEL011070 | C-type lectin, galactose-binding | IMM | -0.94 |
| AAEL009049 | crotonobetainyl-CoA-hydratase, putative | MET | -0.94 |
| AAEL005537 | neuroligin 4 | PROT | -0.94 |
| AAEL013187 | Cytosolic carboxypeptidase 6 | PROT | -0.94 |
| AAEL012025 | mitochondrial ribosomal protein, L45, putative | R/S/M | -0.94 |
| AAEL004357 | neuroligin, putative | R/S/M | -0.94 |
| AAEL010273 | cytochrome P450 | R/S/M | -0.94 |
| AAEL004716 | chromodomain helicase DNA binding protein | R/T/T | -0.94 |
| AAEL010822 | sulfate transporter | TRP | -0.94 |
| AAEL001268 | TRPA1 channel protein | TRP | -0.94 |
| AAEL009922 | conserved hypothetical protein | UNK | -0.94 |
| AAEL000521 | hypothetical protein | UNK | -0.94 |
| AAEL014061 | conserved hypothetical protein | UNK | -0.94 |
| AAEL008315 | calponin/transgelin | C/S | -0.95 |
| AAEL007306 | alpha-actinin | C/S | -0.95 |
| AAEL004883 | guanylate cyclase | DIV | -0.95 |
| AAEL012269 | serine/threonine kinase NLK | DIV | -0.95 |
| AAEL003297 | alkaline phosphatase | DIV | -0.95 |
| AAEL009723 | FBN11 | IMM | -0.95 |
| AAEL003607 | FBN27 | IMM | -0.95 |
| Aaeg:N46074 | TPx | IMM | -0.95 |
| AAEL014724 | clip-domain serine protease, putative | IMM | -0.95 |
| AAEL010671 | oxidoreductase | R/S/M | -0.95 |
| AAEL009279 | 60S ribosomal protein L7 | R/T/T | -0.95 |
| AAEL002980 | U1 small nuclear ribonucleoprotein A | R/T/T | -0.95 |
| AAEL008282 | substance P receptor (long form), putative | TRP | -0.95 |
| AAEL001328 | hypothetical protein | UNK | -0.95 |
| AAEL008747 | hypothetical protein | UNK | -0.95 |
| AAEL008951 | hypothetical protein | UNK | -0.95 |
| AAEL007506 | lava lamp protein | C/S | -0.96 |
| AAEL000233 | membrane-associated guanylate kinase, putative | DIV | -0.96 |
| AAEL008676 | Acylglycerol kinase, mitochondrial | DIV | -0.96 |
| AAEL008319 | protein disulfide isomerase | DIV | -0.96 |
| AAEL012741 | zinc finger transcription factor SMA-9 | DIV | -0.96 |
| AAEL013425 | U7 snRNA-associated Sm-like protein LSm10 | DIV | -0.96 |
| AAEL003487 | development and differentiation-enhancing factor, ddef | DIV | -0.96 |
| AAEL008556 | prp4 | DIV | -0.96 |
| AAEL012386 | ATP-binding cassette transporter | TRP | -0.96 |
| AAEL010608 | succinate dehydrogenase | TRP | -0.96 |
| AAEL013894 | hypothetical protein | UNK | -0.96 |
| AAEL008725 | conserved hypothetical protein | UNK | -0.96 |
| AAEL015124 | hypothetical protein | UNK | -0.96 |
| AAEL002776 | conserved hypothetical protein | UNK | -0.96 |
| AAEL011043 | cuticular protein 62Bb, isoform A | C/S | -0.97 |
| AAEL013229 | tubulin alpha chain | C/S | -0.97 |
| AAEL002167 | Gustatory receptor 21a, putative | CSR | -0.97 |
| AAEL013853 | C-type lectin, galactose-binding | DIV | -0.97 |
| AAEL010369 | phospholipase b, plb1 | DIV | -0.97 |
| AAEL013312 | dual-specificity protein phosphatase, putative | DIV | -0.97 |
| AAEL012195 | small GTPase, putative | DIV | -0.97 |
| AAEL013595 | Nose resistant to fluoxetine protein 6 | DIV | -0.97 |
| AAEL010619 | costa | DIV | -0.97 |
| AAEL011834 | SNARE protein TLG2/syntaxin 16 | DIV | -0.97 |
| AAEL000171 | zinc finger protein, putative | DIV | -0.97 |
| AAEL007185 | synbindin | DIV | -0.97 |
| AAEL000074 | serine protease | IMM | -0.97 |
| Aaeg:N24616 | SOD | IMM | -0.97 |
| AAEL014824 | SOCS box protein | IMM | -0.97 |
| AAEL014493 | aldehyde oxidase | R/S/M | -0.97 |
| AAEL001488 | ribosomal protein L15 | R/T/T | -0.97 |
| AAEL005397 | conserved hypothetical protein | UNK | -0.97 |
| AAEL010882 | conserved hypothetical protein | UNK | -0.97 |
| AAEL012090 | cuticular protein hypothetical 28 | C/S | -0.98 |
| AAEL007268 | WW domain-containing protein 1 | C/S | -0.98 |
| AAEL012331 | ubiquitin ligase E3 alpha | DIV | -0.98 |
| AAEL005717 | zinc finger protein, putative | DIV | -0.98 |
| AAEL002722 | protein kinase C inhibitor, putative | DIV | -0.98 |
| AAEL004893 | sugar ABC transporter, ATP-binding protein | DIV | -0.98 |
| AAEL005436 | diphteria toxin resistance protein 2, dph2 | DIV | -0.98 |
| AAEL012952 | N-acetyltransferase 2 | DIV | -0.98 |
| AAEL000610 | U3 snoRNP protein | DIV | -0.98 |
| AAEL004233 | lachesin precursor, putative | DIV | -0.98 |
| AAEL006302 | spire | DIV | -0.98 |
| AAEL001591 | zinc finger protein | DIV | -0.98 |
| AAEL005566 | bric a brac-like protein | DIV | -0.98 |
| AAEL007035 | tak1 | IMM | -0.98 |
| AAEL000709 | CACTUS | IMM | -0.98 |
| AAEL011618 | CTL | IMM | -0.98 |
| AAEL001932 | Death domain-containing adapter protein BG4 | IMM | -0.98 |
| AAEL013936 | serpin-4A | IMM | -0.98 |
| AAEL013143 | ankyrin repeat domain-containing protein | IMM | -0.98 |
| AAEL010960 | xaa-pro dipeptidase app(e.coli) | MET | -0.98 |
| AAEL010037 | phosphoglucomutase | MET | -0.98 |
| AAEL006317 | short-chain dehydrogenase | R/S/M | -0.98 |
| AAEL004198 | conserved hypothetical protein | UNK | -0.98 |
| AAEL009728 | conserved hypothetical protein | UNK | -0.98 |
| AAEL010300 | conserved hypothetical protein | UNK | -0.98 |
| AAEL011691 | hypothetical protein | UNK | -0.98 |
| AAEL008119 | hypothetical protein | UNK | -0.98 |
| AAEL000822 | kinesin-like protein KLP68D | C/S | -0.99 |
| AAEL010837 | SR-related CTD associated factor 6 | DIV | -0.99 |
| AAEL014839 | sidestep protein | DIV | -0.99 |
| AAEL005605 | adenylsulfate kinase | DIV | -0.99 |
| AAEL007120 | lim homeobox protein | DIV | -0.99 |
| AAEL005410 | erythroblast macrophage protein emp | DIV | -0.99 |
| AAEL006013 | huntingtin interacting protein | DIV | -0.99 |
| AAEL006877 | PPO4 | IMM | -0.99 |
| AAEL011764 | PPO10 | IMM | -0.99 |
| AAEL007515 | delta(9)-desaturase, putative | MET | -0.99 |
| AAEL014035 | suppressor of actin (sac) | TRP | -0.99 |
| AAEL012569 | hypothetical protein | UNK | -0.99 |
| AAEL012884 | conserved hypothetical protein | UNK | -0.99 |
| AAEL010025 | conserved hypothetical protein | UNK | -0.99 |
| AAEL002238 | hypothetical protein | UNK | -0.99 |
| AAEL008311 | mitogen activated protein kinase kinase kinase 5, mapkkk5, mekk5 | DIV | -1 |
| AAEL007702 | chaperonin | DIV | -1 |
| AAEL004409 | yellow protein, putative | DIV | -1 |
| AAEL008000 | golgi reassembly stacking protein 2 (grasp2) | DIV | -1 |
| AAEL004106 | hairy protein | DIV | -1 |
| AAEL001134 | methylmalonate-semialdehyde dehydrogenase | DIV | -1 |
| AAEL012963 | lipid storage droplets surface binding protein | DIV | -1 |
| AAEL014601 | alkaline phosphatase | DIV | -1 |
| AAEL012193 | Zinc finger protein 92 | DIV | -1 |
| AAEL011443 | phosphatidylinositol transfer protein SEC14 | DIV | -1 |
| AAEL002885 | protein kinase c zeta and iota isoform | DIV | -1 |
| AAEL003697 | serpin-17 | IMM | -1 |
| Aaeg:N44241 | GPx | IMM | -1 |
| AAEL001077 | proclotting enzyme | IMM | -1 |
| AAEL003402 | sphingomyelin phosphodiesterase | MET | -1 |
| AAEL006524 | venom allergen | MET | -1 |
| AAEL009131 | cytochrome P450 | R/S/M | -1 |
| AAEL009472 | acidic ribosomal protein P1, putative | R/T/T | -1 |
| AAEL008146 | zinc transporter | TRP | -1 |
| AAEL009453 | voltage-gated potassium channel | TRP | -1 |
| AAEL001832 | hypothetical protein | UNK | -1 |
| AAEL004387 | hypothetical protein | UNK | -1 |
| AAEL014107 | conserved hypothetical protein | UNK | -1 |
| AAEL008226 | hypothetical protein | UNK | -1 |
| AAEL001337 | band 4.1-like protein 2, putative | C/S | -1.01 |
| AAEL004772 | pupal cuticle protein, putative | C/S | -1.01 |
| AAEL006066 | peptidyl-trna hydrolase | DIV | -1.01 |
| AAEL005276 | target of myb1 (tom1) | DIV | -1.01 |
| AAEL005098 | cysteinech venom protein, putative | DIV | -1.01 |
| AAEL005930 | ubiquitin-protein ligase | DIV | -1.01 |
| AAEL001160 | zinc finger protein | DIV | -1.01 |
| AAEL010199 | Activating signal cointegrator 1 complex subunit | DIV | -1.01 |
| AAEL013247 | UDP-N-acetylmuramyl pentapeptide synthase | DIV | -1.01 |
| AAEL007260 | lethal (3) neo38, isoform A | DIV | -1.01 |
| AAEL004788 | Osiris, putative | DIV | -1.01 |
| AAEL005107 | Kaz1-ORFB, isoform C | PROT | -1.01 |
| AAEL002612 | variable region-containing chitin-binding protein | REDOX | -1.01 |
| AAEL007539 | hypothetical protein | UNK | -1.01 |
| AAEL008684 | serrano protein | DIV | -1.02 |
| AAEL002390 | zinc finger protein | DIV | -1.02 |
| AAEL001458 | heparan sulfate sulfotransferase | DIV | -1.02 |
| AAEL011611 | serine protease, putative | IMM | -1.02 |
| AAEL003475 | aromatic amino acid decarboxylase | IMM | -1.02 |
| AAEL003612 | peroxinectin | IMM | -1.02 |
| AAEL014658 | caspase-s20 | IMM | -1.02 |
| AAEL009232 | long-chain-fatty-acid coa ligase | MET | -1.02 |
| AAEL015053 | conserved hypothetical protein | UNK | -1.02 |
| AAEL005843 | conserved hypothetical protein | UNK | -1.02 |
| AAEL001980 | protein serine/threonine kinase, putative | CSR | -1.03 |
| AAEL011065 | RhoGEF2, isoform D | DIV | -1.03 |
| AAEL004064 | meiotic checkpoint regulator cut4 | DIV | -1.03 |
| AAEL011592 | secreted mucin MUC17, putative | DIV | -1.03 |
| AAEL006607 | Juvenile hormone-inducible protein, putative | DIV | -1.03 |
| AAEL000425 | Flap endonuclease GEN | DIV | -1.03 |
| AAEL011991 | serine protease | IMM | -1.03 |
| AAEL009410 | amine oxidase | R/S/M | -1.03 |
| AAEL013249 | eukaryotic translation initiation factor 4 gamma | R/T/T | -1.03 |
| AAEL006215 | synaptopodin 2-like protein-like | R/T/T | -1.03 |
| AAEL008386 | ATP-binding cassette sub-family A member 3, putative | TRP | -1.03 |
| AAEL001454 | hypothetical protein | UNK | -1.03 |
| AAEL015276 | conserved hypothetical protein | UNK | -1.03 |
| AAEL001661 | hypothetical protein | UNK | -1.03 |
| AAEL009796 | cuticle protein, putative | C/S | -1.04 |
| AAEL011507 | zinc finger protein | DIV | -1.04 |
| AAEL002997 | serine protease | IMM | -1.04 |
| AAEL005718 | serine protease, putative | IMM | -1.04 |
| AAEL012380 | PPGRP LA | IMM | -1.04 |
| AAEL003889 | GNBPB1 | IMM | -1.04 |
| AAEL009097 | Cathepsin K | PROT | -1.04 |
| AAEL002098 | DNA repair endonuclease xp-f / mei-9 / rad1 | R/T/T | -1.04 |
| AAEL003897 | DNA repair protein xp-c / rad4 | R/T/T | -1.04 |
| AAEL005256 | hypothetical protein | UNK | -1.04 |
| AAEL010406 | hypothetical protein | UNK | -1.04 |
| AAEL008557 | conserved hypothetical protein | UNK | -1.04 |
| AAEL008896 | hypothetical protein | UNK | -1.04 |
| AAEL004334 | hypothetical protein | UNK | -1.04 |
| AAEL011537 | hypothetical protein | UNK | -1.04 |
| AAEL000258 | conserved hypothetical protein | UNK | -1.04 |
| AAEL013973 | hypothetical protein | UNK | -1.04 |
| AAEL007884 | conserved membrane protein at 44E, putative | DIV | -1.05 |
| AAEL001217 | tripartite motif protein 45 | DIV | -1.05 |
| AAEL008794 | DNA-directed RNA polymerase III subunit D | DIV | -1.05 |
| AAEL010909 | zinc finger protein 592 | DIV | -1.05 |
| AAEL002839 | kakapo | DIV | -1.05 |
| AAEL014864 | zinc finger protein 555 | DIV | -1.05 |
| AAEL011622 | serine protease, putative | IMM | -1.05 |
| AAEL005403 | conserved hypothetical protein | UNK | -1.05 |
| AAEL005474 | hypothetical protein | UNK | -1.05 |
| AAEL014126 | hypothetical protein | UNK | -1.05 |
| AAEL003806 | hypothetical protein | UNK | -1.05 |
| AAEL003373 | hypothetical protein | UNK | -1.05 |
| AAEL014750 | pyruvate dehydrogenase | DIV | -1.06 |
| AAEL001587 | zinc finger protein with KRAB and SCAN domain | DIV | -1.06 |
| AAEL012356 | somatostatin receptor | DIV | -1.06 |
| AAEL010992 | CTL8 | IMM | -1.06 |
| AAEL012762 | cytochrome P450 | R/S/M | -1.06 |
| AAEL007126 | sugar transporter | TRP | -1.06 |
| AAEL001858 | hypothetical protein | UNK | -1.06 |
| AAEL003535 | hypothetical protein | UNK | -1.06 |
| AAEL001290 | conserved hypothetical protein | UNK | -1.06 |
| AAEL002169 | conserved hypothetical protein | UNK | -1.06 |
| AAEL005783 | conserved hypothetical protein | UNK | -1.06 |
| AAEL012917 | conserved hypothetical protein | UNK | -1.06 |
| AAEL001628 | hypothetical protein | UNK | -1.06 |
| AAEL001561 | conserved hypothetical protein | UNK | -1.06 |
| AAEL009937 | calcium/calmodulin-dependent serine protein kinase membrane-associated guanylate kinase (cask) | DIV | -1.07 |
| AAEL005522 | IWS1-like protein | DIV | -1.07 |
| AAEL009022 | adenylate cyclase type | DIV | -1.07 |
| AAEL000693 | fkbppamycin associated protein | MET | -1.07 |
| AAEL012785 | lumbrokinase-3(1) precursor, putative | MET | -1.07 |
| AAEL010600 | hypoxia associated factor | MET | -1.07 |
| AAEL002695 | hypothetical protein | UNK | -1.07 |
| AAEL001502 | hypothetical protein | UNK | -1.07 |
| AAEL001012 | hypothetical protein | UNK | -1.07 |
| AAEL012053 | conserved hypothetical protein | UNK | -1.07 |
| AAEL011186 | conserved hypothetical protein | UNK | -1.07 |
| AAEL008209 | hypothetical protein | UNK | -1.07 |
| AAEL015296 | septin | C/S | -1.08 |
| AAEL009735 | iodotyrosine dehalogenase | DIV | -1.08 |
| AAEL003480 | zinc finger protein 708-like | DIV | -1.08 |
| AAEL003459 | failed axon connections protein | DIV | -1.08 |
| AAEL005029 | zinc finger protein | DIV | -1.08 |
| AAEL005943 | ser/thr kinase stk11 (lkb1) | DIV | -1.08 |
| AAEL004386 | HPX8C | IMM | -1.08 |
| AAEL000749 | angiopoietin like salivary protein | IMM | -1.08 |
| AAEL010917 | organic anion transporter | TRP | -1.08 |
| AAEL015288 | conserved hypothetical protein | UNK | -1.08 |
| AAEL015451 | hypothetical protein | UNK | -1.08 |
| AAEL001261 | hypothetical protein | UNK | -1.08 |
| AAEL013935 | conserved hypothetical protein | UNK | -1.08 |
| AAEL003755 | BolA protein, putative | DIV | -1.09 |
| AAEL000931 | alkaline phosphatase | DIV | -1.09 |
| AAEL008229 | disabled, isoform A | DIV | -1.09 |
| AAEL003540 | bicoid-interacting protein 3 | DIV | -1.09 |
| AAEL004041 | flotillin-2 | DIV | -1.09 |
| AAEL010912 | dipeptidyl-peptidase | MET | -1.09 |
| AAEL009692 | signal transducer and activator of transcription | R/S/M | -1.09 |
| AAEL000958 | conserved hypothetical protein | UNK | -1.09 |
| AAEL011767 | hypothetical protein | UNK | -1.09 |
| AAEL000601 | 3-hydroxy-3-methylglutaryl-coenzyme A reductase | DIV | -1.1 |
| AAEL012694 | Juvenile hormone-inducible protein, putative | DIV | -1.1 |
| AAEL008360 | 5-hydroxytryptamine receptor 2 | DIV | -1.1 |
| AAEL004641 | PTS system IIA component domain-containing protein | DIV | -1.1 |
| AAEL007211 | TBC1 domain family member 5 | DIV | -1.1 |
| AAEL014289 | zinc finger protein 774 | DIV | -1.1 |
| AAEL014001 | yellow protein precursor, putative | DIV | -1.1 |
| AAEL006462 | TFIID subunit TAFII55, putative | DIV | -1.1 |
| AAEL006326 | deoxyribonuclease I, putative | DIV | -1.1 |
| AAEL002499 | Protein bric-a-brac 2 | DIV | -1.1 |
| AAEL002159 | zinc finger protein 436 | DIV | -1.1 |
| AAEL009051 | TPX5 | IMM | -1.1 |
| AAEL003163 | forkhead protein/ forkhead protein domain | R/T/T | -1.1 |
| AAEL013529 | synapsin | TRP | -1.1 |
| AAEL012444 | organic cation transporter | TRP | -1.1 |
| AAEL014602 | conserved hypothetical protein | UNK | -1.1 |
| AAEL006255 | conserved hypothetical protein | UNK | -1.1 |
| AAEL006852 | hypothetical protein | UNK | -1.1 |
| AAEL007217 | conserved hypothetical protein | UNK | -1.1 |
| AAEL014454 | hypothetical protein | UNK | -1.1 |
| AAEL013829 | cuticle protein, putative | C/S | -1.11 |
| AAEL013185 | beat protein, putative | DIV | -1.11 |
| AAEL010105 | MRAS2, putative | DIV | -1.11 |
| AAEL011567 | protein phosphatase 2c | DIV | -1.11 |
| AAEL000256 | epithelial membrane protein | IMM | -1.11 |
| AAEL015138 | Niemann-Pick Type C-2, putative | IMM | -1.11 |
| AAEL014150 | transcription factor IIIA, putative | R/T/T | -1.11 |
| AAEL012916 | conserved hypothetical protein | UNK | -1.11 |
| AAEL012434 | conserved hypothetical protein | UNK | -1.11 |
| AAEL012644 | peritrophin-like protein | C/S | -1.12 |
| AAEL008566 | g-protein coupled receptor | DIV | -1.12 |
| AAEL001384 | triple functional domain, trio | DIV | -1.12 |
| Aaeg:N18481 | SOCS | IMM | -1.12 |
| AAEL003253 | CLIP13B | IMM | -1.12 |
| AAEL001036 | ikappab kinase complex-associated protein | IMM | -1.12 |
| AAEL011126 | alcohol dehydrogenase | MET | -1.12 |
| AAEL001615 | mitochondrial ribosomal protein, S18C, putative | R/S/M | -1.12 |
| AAEL006883 | Growth arrest and DNA-damage-inducible protein | R/T/T | -1.12 |
| AAEL001620 | organic anion transporter | TRP | -1.12 |
| AAEL005782 | conserved hypothetical protein | UNK | -1.12 |
| AAEL001186 | hypothetical protein | UNK | -1.12 |
| AAEL006398 | odorant binding protein 1 | CSR | -1.13 |
| AAEL011360 | 15E1.2 protein | DIV | -1.13 |
| AAEL000202 | t-box protein | DIV | -1.13 |
| AAEL000170 | zinc finger protein | DIV | -1.13 |
| AAEL012143 | caspase-s7 ice | IMM | -1.13 |
| AAEL002688 | glucosyl/glucuronosyl transferases | MET | -1.13 |
| AAEL013605 | Kaz1-ORFB, isoform C | PROT | -1.13 |
| AAEL011389 | conserved hypothetical protein | UNK | -1.13 |
| AAEL013480 | hypothetical protein | UNK | -1.13 |
| AAEL005558 | X box binding protein-1 | DIV | -1.14 |
| AAEL000894 | hunchback protein | DIV | -1.14 |
| AAEL011168 | GTP-binding protein (i) alpha subunit, gnai | DIV | -1.14 |
| AAEL004388 | HPX8A | IMM | -1.14 |
| Aaeg:N32065 | CTL | IMM | -1.14 |
| AAEL002699 | serpin-7 | IMM | -1.14 |
| AAEL015109 | serine protease | IMM | -1.14 |
| AAEL014557 | homeobox protein cdx | R/T/T | -1.14 |
| AAEL010783 | sodium/potassium-dependent atpase beta-2 subunit | TRP | -1.14 |
| AAEL007809 | potassium channel regulator | TRP | -1.14 |
| AAEL002861 | conserved hypothetical protein | UNK | -1.14 |
| AAEL013792 | cuticular protein 10 from Low Complexity family | DIV | -1.15 |
| AAEL013731 | DNA polymerase III polC-type | DIV | -1.15 |
| AAEL006736 | Protein singed wings 2 (LRR) | IMM | -1.15 |
| AAEL011129 | alcohol dehydrogenase | MET | -1.15 |
| AAEL003195 | carboxylesterase | R/S/M | -1.15 |
| AAEL013092 | cation chloride cotransporter | TRP | -1.15 |
| AAEL003347 | CRAL/TRIO domain-containing protein | TRP | -1.15 |
| AAEL004535 | hypothetical protein | UNK | -1.15 |
| AAEL005361 | hypothetical protein | UNK | -1.15 |
| AAEL012638 | conserved hypothetical protein | UNK | -1.15 |
| AAEL014368 | sap18 | DIV | -1.16 |
| AAEL002734 | zinc finger protein | DIV | -1.16 |
| AAEL009098 | glycosyl transferase family protein | DIV | -1.16 |
| AAEL002665 | matrix metalloproteinase | PROT | -1.16 |
| AAEL006800 | sodium/chloride dependent transporter | TRP | -1.16 |
| AAEL005937 | ATP-binding cassette transporter | TRP | -1.16 |
| AAEL002180 | hypothetical protein | UNK | -1.16 |
| AAEL008156 | conserved hypothetical protein | UNK | -1.16 |
| AAEL006377 | membrane glycoprotein LIG-1 | DIV | -1.17 |
| AAEL001396 | mandelate racemase | DIV | -1.17 |
| AAEL011362 | gastrula zinc finger protein XFG20-1 | DIV | -1.17 |
| AAEL013329 | cdk1 | DIV | -1.17 |
| AAEL004524 | proacrosin, putative | MET | -1.17 |
| AAEL015588 | hydroxyacyl dehydrogenase | MET | -1.17 |
| AAEL005101 | carboxylesterase | R/S/M | -1.17 |
| AAEL009599 | odorant binding protein | CSR | -1.18 |
| AAEL005827 | katanin P80 subunit | DIV | -1.18 |
| AAEL006688 | Probable splicing factor 3B subunit 5 | DIV | -1.18 |
| AAEL011850 | cytochrome P450 | R/S/M | -1.18 |
| AAEL004555 | hypothetical protein | UNK | -1.18 |
| AAEL001662 | kinesin family member 21A | C/S | -1.19 |
| AAEL002473 | fermentation associated protein (Csf1), putative | DIV | -1.19 |
| AAEL006600 | Juvenile hormone-inducible protein, putative | DIV | -1.19 |
| AAEL003849 | DEFE | IMM | -1.19 |
| AAEL011608 | PRGP-LD | IMM | -1.19 |
| AAEL009240 | neurotactin | R/S/M | -1.19 |
| AAEL003173 | forkhead protein/ forkhead protein domain | R/T/T | -1.19 |
| AAEL002541 | cystinosin | TRP | -1.19 |
| AAEL013462 | conserved hypothetical protein | UNK | -1.19 |
| AAEL009205 | Feline leukemia virus subgroup C receptor-related | DIV | -1.2 |
| AAEL005685 | Low-density lipoprotein receptor-related protein | DIV | -1.2 |
| AAEL011289 | Uncharacterized protein KIAA0564-like protein | DIV | -1.2 |
| AAEL002301 | serine protease | IMM | -1.2 |
| AAEL002279 | transcription factor grauzone | DIV | -1.21 |
| AAEL001520 | conserved hypothetical protein | UNK | -1.21 |
| AAEL012300 | conserved hypothetical protein | UNK | -1.21 |
| AAEL006620 | conserved hypothetical protein | UNK | -1.21 |
| AAEL007164 | vitamin D | CSR | -1.22 |
| AAEL006368 | trypsin-beta, putative | DIG | -1.22 |
| AAEL007103 | p37NB protein, putative (LRR) | IMM | -1.22 |
| AAEL013025 | conserved hypothetical protein | UNK | -1.22 |
| AAEL007456 | zinc finger protein, putative | DIV | -1.23 |
| AAEL000906 | guanyl-nucleotide exchange factor | DIV | -1.23 |
| AAEL012322 | hypothetical protein | UNK | -1.23 |
| AAEL014486 | quick-to-court, isoform B | DIV | -1.24 |
| AAEL002710 | hypothetical protein | UNK | -1.24 |
| AAEL011838 | U5 snRNP-specific protein | DIV | -1.25 |
| AAEL002796 | l-asparaginase i | DIV | -1.25 |
| AAEL002428 | BTB/POZ domain-containing protein 3 | DIV | -1.25 |
| AAEL006571 | IRAK | IMM | -1.25 |
| AAEL010179 | nadph oxidase | R/S/M | -1.25 |
| AAEL011808 | glucose dehydrogenase | TRP | -1.25 |
| AAEL007128 | sugar transporter | TRP | -1.25 |
| AAEL004239 | conserved hypothetical protein | UNK | -1.25 |
| AAEL008944 | hypothetical protein | UNK | -1.25 |
| AAEL005559 | myosin light chain kinase | C/S | -1.26 |
| AAEL000051 | Odorant-binding protein 56e, putative | CSR | -1.26 |
| AAEL005752 | lysosomal alpha-mannosidase (mannosidase alpha class 2b member 1) | MET | -1.26 |
| AAEL005026 | ATP-dependent bile acid permease | TRP | -1.26 |
| AAEL003878 | hypothetical protein | UNK | -1.26 |
| AAEL007079 | conserved hypothetical protein | UNK | -1.26 |
| AAEL014520 | follistatin | DIV | -1.27 |
| AAEL014192 | BTB/POZ domain-containing protein 3 | DIV | -1.27 |
| AAEL014833 | hypothetical protein | UNK | -1.27 |
| AAEL011861 | F-box/LRR-repeat protein 16 | DIV | -1.28 |
| AAEL004390 | HPX8B | IMM | -1.28 |
| AAEL010735 | aromatic amino acid decarboxylase | IMM | -1.28 |
| AAEL015033 | conserved hypothetical protein | UNK | -1.28 |
| AAEL012930 | lethal (2) NC136 | DIV | -1.29 |
| AAEL000107 | zinc finger X-linked protein ZXDA | DIV | -1.29 |
| AAEL000441 | hypothetical protein | UNK | -1.29 |
| AAEL004437 | dual-specificity protein phosphatase, putative | DIV | -1.3 |
| AAEL013748 | CTL9 | IMM | -1.3 |
| AAEL006746 | DEFD | IMM | -1.3 |
| AAEL011196 | conserved hypothetical protein | UNK | -1.3 |
| AAEL011913 | serine-type enodpeptidase, putative | DIG | -1.31 |
| AAEL014385 | CTLG4 | IMM | -1.31 |
| AAEL013554 | cytochrome P450 | R/S/M | -1.31 |
| AAEL002720 | serpin-20 | IMM | -1.32 |
| AAEL002036 | hypothetical protein | UNK | -1.32 |
| AAEL013716 | growl, isoform B | DIV | -1.33 |
| AAEL012574 | synaptic vesicle protein | DIV | -1.33 |
| AAEL012938 | zinc finger protein | DIV | -1.34 |
| AAEL001906 | pnuts protein | DIV | -1.34 |
| AAEL002335 | atbf1 | DIV | -1.34 |
| AAEL009730 | type 1 envelope glycoprotein gp350 | DIV | -1.35 |
| AAEL011130 | alcohol dehydrogenase | MET | -1.35 |
| AAEL007916 | conserved hypothetical protein | UNK | -1.35 |
| AAEL009557 | Niemann-Pick Type C-2, putative | IMM | -1.36 |
| AAEL005001 | aquaporin | TRP | -1.36 |
| AAEL015005 | zinc finger protein 394 | DIV | -1.37 |
| AAEL012003 | keratinocyte lectin, putative | IMM | -1.37 |
| AAEL000828 | vitellogenin, putative | MET | -1.37 |
| AAEL010194 | hypothetical protein | UNK | -1.37 |
| AAEL009566 | apolipoprotein D, putative | DIV | -1.38 |
| AAEL003843 | Defensin-like protein | IMM | -1.38 |
| AAEL006992 | cytochrome P450 | R/S/M | -1.38 |
| AAEL009514 | conserved hypothetical protein | UNK | -1.38 |
| AAEL011636 | hypothetical protein | UNK | -1.39 |
| AAEL007027 | sodium/solute symporter | TRP | -1.4 |
| AAEL002850 | patched 1, putative | DIV | -1.41 |
| AAEL005047 | conserved hypothetical protein | UNK | -1.41 |
| AAEL005533 | synaptic vesicle protein | DIV | -1.42 |
| AAEL003038 | conserved hypothetical protein | UNK | -1.42 |
| AAEL000592 | auxin efflux carrier superfamily | DIV | -1.43 |
| AAEL000072 | zinc carboxypeptidase | PROT | -1.43 |
| AAEL010921 | organic anion transporter | TRP | -1.43 |
| AAEL010189 | Band 7 protein | DIV | -1.44 |
| AAEL000196 | conserved hypothetical protein | UNK | -1.44 |
| AAEL010828 | conserved hypothetical protein | UNK | -1.45 |
| AAEL002294 | sulfate transporter | TRP | -1.46 |
| AAEL014898 | hypothetical protein | UNK | -1.46 |
| AAEL014274 | conserved hypothetical protein | UNK | -1.47 |
| AAEL004342 | odorant-binding protein OBPjj83a | CSR | -1.48 |
| AAEL000099 | serine protease | IMM | -1.48 |
| AAEL011634 | FBN12 | IMM | -1.49 |
| AAEL002680 | AMP dependent ligase | MET | -1.49 |
| AAEL001110 | jnk stimulatory phosphatase (jsp1) | DIV | -1.52 |
| AAEL001902 | glutamate decarboxylase | IMM | -1.52 |
| AAEL005064 | serine protease | IMM | -1.52 |
| AAEL011610 | galactose-specific C-type lectin, putative | IMM | -1.55 |
| AAEL000354 | dimeric dihydrodiol dehydrogenase | MET | -1.57 |
| AAEL000777 | CECJ | IMM | -1.6 |
| AAEL001855 | zinc carboxypeptidase | PROT | -1.61 |
| AAEL012524 | AMP dependent ligase | MET | -1.62 |
| AAEL012646 | mucin-like protein 1 | DIV | -1.64 |
| AAEL015450 | ribonuclease UK114, putative | R/T/T | -1.65 |
| AAEL003821 | defensin | IMM | -1.67 |
| AAEL006984 | cytochrome P450 | R/S/M | -1.68 |
| AAEL007051 | lipase | MET | -1.69 |
| AAEL000819 | hypothetical protein | UNK | -1.69 |
| AAEL004240 | gamma glutamyl transpeptidases | MET | -1.72 |
| AAEL002794 | Transcriptional regulator, PadR family domain | R/T/T | -1.72 |
| AAEL006704 | FBN18 | IMM | -1.73 |
| AAEL014188 | serine-type enodpeptidase, putative | DIG | -1.74 |
| AAEL013338 | lethal(2)essential for life protein, l2efl | DIV | -1.76 |
| AAEL005959 | phospholipase b, plb1 | DIV | -1.76 |
| AAEL004126 | sterol desaturase | MET | -1.93 |
| AAEL009962 | von Willebrand factor type A domain-containing | DIV | -1.95 |
| AAEL002662 | elongase, putative | MET | -1.97 |
| AAEL009181 | conserved hypothetical protein | UNK | -1.99 |
| AAEL000029 | dopachrome-conversion enzyme (DCE), putative | IMM | -2.03 |
| AAEL003426 | sodium-dependent phosphate transporter | TRP | -2.03 |
| AAEL013885 | conserved hypothetical protein | UNK | -2.05 |
| Aaeg:N41092 | CTL | IMM | -2.08 |
| AAEL009111 | sphingomyelin phosphodiesterase | MET | -2.08 |
| AAEL009985 | conserved hypothetical protein | UNK | -2.1 |
| AAEL001813 | sodium/solute symporter | TRP | -2.11 |
| AAEL008568 | glucosyl/glucuronosyl transferases | MET | -2.2 |
| AAEL006583 | multicopper oxidase | DIV | -2.3 |
| AAEL000756 | putative viral A-type inclusion protein | DIV | -2.32 |
| AAEL013126 | putative protein G12 | DIV | -2.56 |
| AAEL009166 | microvillar-like protein | DIV | -2.74 |
| AAEL000176 | conserved hypothetical protein | UNK | -6.91 |
