# Supplementary material for: Transcriptome Analysis of Aedes aegypti Transgenic Mosquitoes with Altered Immunity
Source: PLoS Pathog. 2011 Nov 17;7(11):e1002394. doi: 10.1371/journal.ppat.1002394 (PMC3219725; doi:10.1371/journal.ppat.1002394)
Supplement: Table S7 — Gene repertoire induced in the Aedes aegypti fat body, 24 h after infection with P. gallinaceum . Data obtained by means of a full genome Agilent-based microarray analysis. Gene ID, gene name, functional group and log fold increase (decrease) are presented. Abbreviations are: IMM, immunity; R/S/M, redox, stress and mitochondrion; DIG, blood and sugar food digestive; C/S, cytoskeletal and structural; PROT, proteolysis; TRP, transport; R/T/T, replication, transcription, and translation; MET, metabolism; DIV, diverse functions; UNK, unknown functions. (DOCX) [file ppat.1002394.s012.docx]

Table S7. Gene repertoire induced in the *Aedes aegypti* fat body, 24 h after infection with *P. gallinaceum*. Data obtained by means of a full genome Agilent-based microarray analysis. Gene ID, gene name, functional group and log fold increase (decrease) are presented. Abbreviations are: IMM, immunity; R/S/M, redox, stress and mitochondrion; DIG, blood and sugar food digestive; C/S, cytoskeletal and structural; PROT, proteolysis; TRP, transport; R/T/T, replication, transcription, and translation; MET, metabolism; DIV, diverse functions; UNK, unknown functions.

| GENE ID | Name | Func group | Logfold |
| --- | --- | --- | --- |
| AAEL002859 | anaphase-promoting complex subunit 2 | DIV | 2.61 |
| AAEL005333 | Titin | DIV | 2.59 |
| AAEL009139 | class D atypical G-protein coupled receptor | DIV | 2.49 |
| AAEL007665 | AraC family transcription regulator | DIV | 2.42 |
| AAEL004112 | TPX2 | IMM | 2.37 |
| AAEL004116 | hypothetical protein | UNK | 2.37 |
| AAEL001349 | zinc finger protein | DIV | 2.36 |
| AAEL007924 | neuropeptide f receptor 76f | DIV | 2.35 |
| AAEL005745 | neurokinin-3 receptor, putative | TRP | 2.32 |
| AAEL010272 | conserved hypothetical protein | UNK | 2.32 |
| AAEL005265 | conserved hypothetical protein | UNK | 2.32 |
| AAEL008449 | conserved hypothetical protein | UNK | 2.32 |
| AAEL014056 | HPCH/HPAI aldolase family protein | DIV | 2.31 |
| AAEL008323 | conserved hypothetical protein | UNK | 2.31 |
| AAEL002621 | splicing factor 1 K-like RNA-binding domain protein | DIV | 2.26 |
| AAEL012831 | bestrophin 2,3,4 | DIV | 2.24 |
| AAEL014892 | cytochrome P450 | R/S/M | 2.24 |
| AAEL010724 | conserved hypothetical protein | UNK | 2.24 |
| AAEL014233 | pickpocket | TRP | 2.23 |
| AAEL007746 | bves, isoform A | DIV | 2.22 |
| AAEL003809 | sugar transporter | TRP | 2.22 |
| AAEL008612 | hypothetical protein | UNK | 2.22 |
| AAEL011699 | hypothetical protein | IMM | 2.21 |
| AAEL012927 | hypothetical protein | UNK | 2.21 |
| AAEL004140 | hypothetical protein | UNK | 2.21 |
| AAEL013184 | open rectifier K[+] channel 1, isoform B | DIV | 2.19 |
| AAEL006855 | UDP-galactose transporter | TRP | 2.19 |
| AAEL013622 | hypothetical protein | UNK | 2.19 |
| AAEL002752 | anaphase-promoting complex, subunit-5, putative | DIV | 2.17 |
| AAEL011300 | conserved hypothetical protein | UNK | 2.15 |
| AAEL004114 | UNC93A protein, putative | DIV | 2.14 |
| AAEL006944 | Probable palmitoyltransferase ZDHHC5 | R/T/T | 2.14 |
| AAEL001912 | forkhead protein/ forkhead protein domain | R/T/T | 2.14 |
| AAEL012813 | sphingolipid delta 4 desaturase/c-4 hydroxylase protein des2 | MET | 2.13 |
| AAEL015257 | suppressor of cytokine signaling 7 | PROT | 2.13 |
| AAEL004208 | hypothetical protein | UNK | 2.12 |
| AAEL014526 | sideroflexin 1,2,3 | DIV | 2.11 |
| AAEL010862 | bip2 protein | DIV | 2.1 |
| AAEL005945 | beta adrenergic receptor | DIV | 2.1 |
| AAEL001754 | pigment dispersing hormone | DIV | 2.09 |
| AAEL003335 | ionotropic receptor 7b | DIV | 2.09 |
| AAEL011193 | steroid dehydrogenase | MET | 2.09 |
| AAEL009473 | conserved hypothetical protein | UNK | 2.09 |
| AAEL010916 | tyrosine-protein phosphatase, nonceptor type 23, putative | DIV | 2.08 |
| AAEL009136 | DNA binding protein | DIV | 2.08 |
| AAEL011217 | PQ loop repeat-containing protein 3 | DIV | 2.08 |
| AAEL008028 | monocarboxylate transporter | TRP | 2.08 |
| AAEL003962 | thiamine pyrophosphate protein | DIV | 2.07 |
| AAEL012815 | hypothetical protein | UNK | 2.06 |
| AAEL008296 | neuropeptide y receptor (npy-r) (pr4 receptor) | DIV | 2.05 |
| AAEL008686 | vlc | DIV | 2.05 |
| AAEL005012 | ionotropic receptor 93a, isoform A | DIV | 2.05 |
| AAEL005512 | speckle-type poz protein | DIV | 2.05 |
| AAEL003115 | tartan (LRR) | IMM | 2.05 |
| AAEL002992 | sphingolipid delta 4 desaturase/c-4 hydroxylase protein des2 | MET | 2.05 |
| AAEL012480 | sodium/calcium exchanger | TRP | 2.05 |
| AAEL006757 | hypothetical protein | UNK | 2.05 |
| AAEL002654 | hypothetical protein | UNK | 2.05 |
| AAEL013180 | hypothetical protein | UNK | 2.05 |
| AAEL008457 | amino acid racemase | DIG | 2.03 |
| AAEL007523 | peroxisomal n1-acetyl-spermine/spermidine oxidase | R/S/M | 2.03 |
| AAEL001555 | conserved hypothetical protein | UNK | 2.03 |
| AAEL007743 | hypothetical protein | UNK | 2.02 |
| AAEL004373 | hypothetical protein | UNK | 2.02 |
| AAEL000342 | peroxidasin | IMM | 2.01 |
| AAEL012248 | histamine-gated chloride channel subunit | TRP | 2.01 |
| AAEL014490 | conserved hypothetical protein | UNK | 2.01 |
| AAEL004354 | hypothetical protein | UNK | 2.01 |
| AAEL007282 | syntaxin binding protein-1,2,3 | DIV | 2 |
| AAEL003364 | bifocal, isoform B | DIV | 1.99 |
| AAEL003902 | d(1a,b) dopamine receptor | DIV | 1.99 |
| AAEL008338 | ion channel nompc | TRP | 1.99 |
| AAEL000453 | polyprotein | DIV | 1.98 |
| AAEL001222 | hypothetical protein | UNK | 1.98 |
| AAEL006062 | neuralized | DIV | 1.97 |
| AAEL002760 | conserved hypothetical protein | UNK | 1.97 |
| AAEL010411 | dual specificity protein phosphatase | DIV | 1.96 |
| AAEL005892 | zinc finger protein 367 | DIV | 1.96 |
| AAEL013161 | heat shock protein, putative | R/S/M | 1.96 |
| AAEL004872 | conserved hypothetical protein | UNK | 1.96 |
| AAEL004849 | hypothetical protein | UNK | 1.96 |
| AAEL007053 | receptor protein kinase, putative | DIV | 1.95 |
| AAEL004255 | zinc finger protein | DIV | 1.95 |
| AAEL003014 | zinc finger protein | DIV | 1.95 |
| AAEL007363 | leucinech transmembrane protein | IMM | 1.95 |
| AAEL002625 | mucin-like peritrophin | C/S | 1.94 |
| AAEL009531 | niemann-pick C1 | DIV | 1.94 |
| AAEL008817 | hexamerin 2 beta | IMM | 1.94 |
| AAEL014244 | glucosyl/glucuronosyl transferases | MET | 1.94 |
| AAEL007020 | hypothetical protein | UNK | 1.93 |
| AAEL011252 | pre-mRNA splicing factor prp17 | DIV | 1.93 |
| AAEL011505 | integral membrane pore glycoprotein gp210, putative | TRP | 1.93 |
| AAEL000026 | dynein light chain, putative | C/S | 1.91 |
| AAEL002951 | forkhead protein/ forkhead protein domain | R/T/T | 1.91 |
| AAEL004149 | hypothetical protein | UNK | 1.91 |
| AAEL014114 | mlck, drome | DIV | 1.9 |
| AAEL005156 | hypothetical protein | UNK | 1.9 |
| AAEL003208 | Tubby, putative | IMM | 1.89 |
| AAEL010157 | microsomal glutathione s-transferase | R/S/M | 1.89 |
| AAEL011679 | ion channel nompc | TRP | 1.89 |
| AAEL011213 | conserved hypothetical protein | UNK | 1.89 |
| AAEL010431 | ANG12 precursor | DIV | 1.88 |
| AAEL015091 | cys-loop ligand-gated ion channel subunit | DIV | 1.88 |
| AAEL011929 | serine-type enodpeptidase, putative | DIG | 1.88 |
| AAEL006072 | hypothetical protein | UNK | 1.88 |
| AAEL002062 | nuclear hormone receptor ftz-f1 (ftz-f1 alpha) | DIV | 1.87 |
| AAEL011446 | galactose-specific C-type lectin, putative | IMM | 1.87 |
| AAEL014863 | glycogenin | MET | 1.87 |
| AAEL013908 | conserved hypothetical protein | UNK | 1.87 |
| AAEL012598 | hypothetical protein | UNK | 1.87 |
| AAEL012088 | TPA: putative cuticle protein | C/S | 1.86 |
| AAEL013729 | myotonin-protein kinase | DIV | 1.86 |
| AAEL002094 | Zinc finger protein 516 | DIV | 1.86 |
| AAEL011916 | serine-type enodpeptidase, putative | DIG | 1.86 |
| AAEL013723 | polypyrimidine tract binding protein | MET | 1.86 |
| AAEL009323 | carbonic anhydrase precursor | MET | 1.86 |
| AAEL001929 | conserved hypothetical protein | UNK | 1.86 |
| AAEL011064 | hypothetical protein | UNK | 1.86 |
| AAEL003844 | GALE5 | IMM | 1.85 |
| AAEL013748 | C-type lectin | IMM | 1.85 |
| AAEL008460 | hypothetical protein | UNK | 1.85 |
| AAEL003832 | DEFC | IMM | 1.84 |
| AAEL005988 | lysozyme | IMM | 1.84 |
| AAEL012358 | hypothetical protein | UNK | 1.84 |
| AAEL007199 | hypothetical protein | DIV | 1.83 |
| AAEL005723 | conserved hypothetical protein | UNK | 1.83 |
| AAEL006555 | hypothetical protein | UNK | 1.83 |
| AAEL009587 | organic cation transporter | DIV | 1.82 |
| AAEL012164 | SPZ6 | IMM | 1.82 |
| AAEL014699 | abc transporter | TRP | 1.82 |
| AAEL010609 | conserved hypothetical protein | UNK | 1.82 |
| AAEL007744 | hypothetical protein | UNK | 1.82 |
| AAEL007213 | delta(9)-desaturase, putative | MET | 1.81 |
| AAEL011690 | conserved hypothetical protein | UNK | 1.81 |
| AAEL015634 | conserved hypothetical protein | UNK | 1.81 |
| AAEL007867 | hypothetical protein | UNK | 1.8 |
| AAEL006832 | frizzled, putative | DIV | 1.79 |
| AAEL009155 | conserved hypothetical protein | UNK | 1.79 |
| AAEL007661 | rab-40 | IMM | 1.78 |
| AAEL004168 | syntaxin | TRP | 1.78 |
| AAEL007623 | conserved hypothetical protein | UNK | 1.78 |
| AAEL011613 | conserved hypothetical protein | UNK | 1.78 |
| AAEL005756 | putative 67B2 family heat shock protein | DIV | 1.76 |
| AAEL009176 | GNBPB3 | IMM | 1.76 |
| AAEL015517 | conserved hypothetical protein | UNK | 1.76 |
| AAEL006438 | dolichyl glycosyltransferase | DIV | 1.75 |
| AAEL002876 | Cubilin | DIV | 1.75 |
| AAEL006997 | putative trypsin-like inhibitor protein | DIV | 1.75 |
| AAEL007344 | conserved hypothetical protein | UNK | 1.75 |
| AAEL010475 | hypothetical protein | UNK | 1.75 |
| AAEL010846 | hypothetical protein | UNK | 1.75 |
| AAEL003947 | Protein tramtrack, alpha isoform | DIV | 1.74 |
| AAEL006638 | n-acetyllactosaminide beta-1,3-n-acetylglucosaminyltransferase | DIV | 1.74 |
| AAEL002230 | chromodomain helicase DNA binding protein | R/T/T | 1.74 |
| AAEL007454 | conserved hypothetical protein | UNK | 1.74 |
| AAEL006545 | hypothetical protein | UNK | 1.74 |
| AAEL011501 | cuticular protein RR-3 motif 147 | C/S | 1.73 |
| AAEL013797 | Kinesin-like protein CG14535 | DIV | 1.72 |
| AAEL012446 | survivin | MET | 1.72 |
| AAEL001843 | ski oncogene | R/T/T | 1.72 |
| AAEL000384 | vesicular acetylcholine transporter | TRP | 1.72 |
| AAEL014687 | ionotropic receptor 7f | DIV | 1.71 |
| AAEL014688 | ionotropic receptor 7f | DIV | 1.71 |
| AAEL000393 | suppressors of cytokine signalling | IMM | 1.71 |
| AAEL003439 | caspase-S18 | IMM | 1.71 |
| AAEL011700 | phosphatidylinositol glycan, class c | MET | 1.71 |
| AAEL011265 | abc transporter | TRP | 1.71 |
| AAEL013806 | conserved hypothetical protein | UNK | 1.71 |
| AAEL015418 | neuropeptide y receptor (npy-r) (pr4 receptor) | DIV | 1.7 |
| AAEL001478 | bile acid beta-glucosidase, putative | DIV | 1.7 |
| AAEL009173 | fasciclin ii (fas ii) | DIV | 1.69 |
| AAEL002136 | zinc finger protein | DIV | 1.69 |
| AAEL008806 | testis development protein prtd | DIV | 1.69 |
| AAEL006493 | hypothetical protein | UNK | 1.69 |
| AAEL006111 | hypothetical protein | UNK | 1.69 |
| AAEL015190 | frizzled, isoform B | DIV | 1.68 |
| AAEL004583 | conserved hypothetical protein | UNK | 1.68 |
| AAEL000204 | conserved hypothetical protein | UNK | 1.68 |
| AAEL003339 | pita, isoform A | DIV | 1.67 |
| AAEL003163 | forkhead protein/ forkhead protein domain | R/T/T | 1.67 |
| AAEL004447 | hypothetical protein | UNK | 1.67 |
| AAEL007188 | Coiled-coil domain-containing protein 63 | C/S | 1.66 |
| AAEL000700 | cadherin | C/S | 1.66 |
| AAEL005583 | c3f | DIV | 1.66 |
| AAEL013770 | zinc finger protein | DIV | 1.66 |
| AAEL003098 | glucosyl/glucuronosyl transferases | MET | 1.66 |
| AAEL013047 | sphingolipid delta 4 desaturase/c-4 hydroxylase protein des2 | MET | 1.66 |
| AAEL009352 | hypothetical protein | UNK | 1.66 |
| AAEL005933 | hypothetical protein | UNK | 1.66 |
| AAEL004476 | rap1 GTPase-activating protein 2 | DIV | 1.65 |
| AAEL002453 | zinc finger protein | DIV | 1.65 |
| AAEL010500 | glutathione-s-transferase theta, gst | R/S/M | 1.65 |
| AAEL000242 | voltage-gated potassium channel | TRP | 1.65 |
| AAEL009079 | conserved hypothetical protein | UNK | 1.65 |
| AAEL012153 | Endoplasmic reticulum metallopeptidase 1 | PROT | 1.64 |
| AAEL008720 | blistered, isoform B | R/T/T | 1.64 |
| AAEL014240 | hypothetical protein | UNK | 1.64 |
| AAEL012910 | dumpy | DIV | 1.63 |
| AAEL009382 | nucleolar protein 66 | DIV | 1.63 |
| AAEL010171 | PGRP-LB | IMM | 1.63 |
| AAEL014548 | TPX3 | IMM | 1.63 |
| AAEL005575 | transient receptor potential channel 4, putative | TRP | 1.63 |
| AAEL014255 | aquaporin, putative | TRP | 1.63 |
| AAEL002931 | hypothetical protein | UNK | 1.63 |
| AAEL001157 | light protein | DIV | 1.62 |
| AAEL011036 | adult cuticle protein, putative | C/S | 1.61 |
| AAEL008938 | Polymerase delta-interacting protein 3 | DIV | 1.61 |
| AAEL003840 | GALE11 | IMM | 1.61 |
| AAEL001376 | hypothetical protein | UNK | 1.61 |
| AAEL000798 | sterol regulatory element binding protein cleavage-activating protein (srebp cleavage-activating protein) (scap) | DIV | 1.6 |
| AAEL003549 | Tbp-1 | DIV | 1.6 |
| AAEL009954 | ML23 | IMM | 1.6 |
| AAEL007862 | conserved hypothetical protein | UNK | 1.6 |
| AAEL002963 | conserved hypothetical protein | UNK | 1.6 |
| AAEL000662 | conserved hypothetical protein | UNK | 1.6 |
| AAEL000574 | thiol-disulfide isomerase | DIV | 1.59 |
| AAEL015139 | ML22 | IMM | 1.59 |
| AAEL006830 | yellow protein precursor | IMM | 1.59 |
| AAEL013368 | lysosomal acid lipase, putative | MET | 1.59 |
| AAEL006337 | conserved hypothetical protein | UNK | 1.59 |
| AAEL014349 | CLIPB15 | IMM | 1.58 |
| AAEL011532 | hypothetical protein | UNK | 1.58 |
| AAEL000790 | Matrix metalloproteinase-17 | DIV | 1.57 |
| AAEL009573 | 5-hydroxytryptamine receptor 1 | DIV | 1.57 |
| AAEL011845 | tryptophan/tyrosine permease | DIV | 1.57 |
| AAEL013441 | leucinech transmembrane protein | IMM | 1.57 |
| AAEL010947 | conserved hypothetical protein | UNK | 1.57 |
| AAEL014234 | conserved hypothetical protein | UNK | 1.57 |
| AAEL011403 | hypothetical protein | UNK | 1.57 |
| AAEL000036 | hypothetical protein | UNK | 1.57 |
| AAEL014132 | thioredoxin binding protein | DIV | 1.56 |
| AAEL007147 | matrix metalloproteinase 1 | DIV | 1.56 |
| AAEL007216 | elongase, putative | MET | 1.56 |
| AAEL008484 | steroid receptor-interacting snf2 domain protein | MET | 1.55 |
| AAEL013822 | conserved hypothetical protein | UNK | 1.55 |
| AAEL001538 | conserved hypothetical protein | UNK | 1.55 |
| AAEL002191 | cuticle protein, putative | C/S | 1.54 |
| AAEL013882 | tkr | DIV | 1.54 |
| AAEL004344 | zinc finger protein | DIV | 1.54 |
| AAEL002979 | conserved hypothetical protein | UNK | 1.54 |
| AAEL007873 | hypothetical protein | UNK | 1.54 |
| AAEL009783 | cuticle protein, putative | C/S | 1.53 |
| AAEL011831 | transcription factor Sox-2, putative | DIV | 1.53 |
| AAEL009212 | lola | PROT | 1.53 |
| AAEL014542 | hypothetical protein | UNK | 1.53 |
| AAEL002080 | septin interacting protein, putative | DIV | 1.52 |
| AAEL001692 | conserved hypothetical protein | UNK | 1.52 |
| AAEL014727 | osiris 20 | DIV | 1.51 |
| AAEL014359 | scratch | DIV | 1.51 |
| AAEL014496 | NADH-cytochrome b5 reductase | DIV | 1.51 |
| AAEL004174 | t-box transcription factor tbx6 | R/T/T | 1.51 |
| AAEL005623 | hypothetical protein | UNK | 1.51 |
| AAEL003027 | cuticular protein | C/S | 1.5 |
| AAEL011572 | Oct1, putative | DIV | 1.5 |
| AAEL011619 | galactose-specific C-type lectin, putative | IMM | 1.5 |
| AAEL014755 | TEP15 | IMM | 1.5 |
| AAEL011030 | hypothetical protein | UNK | 1.5 |
| AAEL003200 | hypothetical protein | UNK | 1.5 |
| AAEL014622 | Zinc finger CCHC domain-containing protein 2 | DIV | 1.49 |
| AAEL007039 | PGRPS5 | IMM | 1.49 |
| AAEL004167 | zinc finger protein | DIV | 1.48 |
| AAEL009508 | zinc finger protein | DIV | 1.47 |
| AAEL008217 | serine-type enodpeptidase, putative | PROT | 1.47 |
| AAEL001408 | CMRF-35-like molecule 3 | DIV | 1.46 |
| AAEL009442 | Jun dimerization protein 2 | DIV | 1.46 |
| AAEL002715 | SRPN22 | IMM | 1.46 |
| AAEL006375 | sphingomyelin phosphodiesterase | MET | 1.46 |
| AAEL000902 | sugar transporter | TRP | 1.46 |
| AAEL000260 | conserved hypothetical protein | UNK | 1.46 |
| AAEL011165 | conserved hypothetical protein | UNK | 1.46 |
| AAEL002095 | conserved hypothetical protein | UNK | 1.46 |
| AAEL012519 | actin binding protein, putative | C/S | 1.45 |
| AAEL010242 | putative 8.7 kDa secreted protein | DIV | 1.44 |
| AAEL011477 | conserved hypothetical protein | UNK | 1.44 |
| AAEL002731 | SRPN14 | IMM | 1.43 |
| AAEL000320 | cytochrome P450 | R/S/M | 1.43 |
| AAEL008194 | protein phosphatase 2a, regulatory subunit | DIV | 1.42 |
| AAEL007624 | REL2 | IMM | 1.42 |
| AAEL003135 | conserved hypothetical protein | UNK | 1.42 |
| AAEL007793 | alkyldihydroxyacetonephosphate synthase | DIV | 1.41 |
| AAEL010767 | glutamate receptor, putative | DIV | 1.41 |
| AAEL014348 | caspase-1 | IMM | 1.41 |
| AAEL005416 | HPX3 | IMM | 1.41 |
| AAEL011234 | reticulon/nogo receptor (LRR) | IMM | 1.41 |
| AAEL004741 | aquaporin transporter | TRP | 1.41 |
| AAEL007405 | conserved hypothetical protein | UNK | 1.41 |
| AAEL006185 | exocyst componenet sec8 | DIV | 1.4 |
| AAEL007945 | eukaryotic translation initiation factor 3 subunit | R/T/T | 1.4 |
| AAEL010310 | zinc finger protein | DIV | 1.39 |
| AAEL011250 | vlc | DIV | 1.39 |
| AAEL003161 | adenylosuccinate synthetase | DIV | 1.38 |
| AAEL006587 | thiamin pyrophosphokinase 1 | DIV | 1.38 |
| AAEL009444 | hypothetical protein | UNK | 1.38 |
| AAEL014851 | mediator complex subunit rgr-1 | DIV | 1.37 |
| Aaeg:N44241 | GPx | IMM | 1.37 |
| AAEL008374 | E3 ubiquitin-protein ligase nedd-4 | MET | 1.37 |
| AAEL003393 | ATP synthase beta subunit | TRP | 1.37 |
| AAEL012595 | lipoprotein NlpD | DIV | 1.36 |
| AAEL008432 | high-affinity choline transporter | DIV | 1.36 |
| Aaeg:N19306 | TEP | IMM | 1.36 |
| AAEL013169 | conserved hypothetical protein | UNK | 1.36 |
| AAEL003389 | attacin | IMM | 1.35 |
| AAEL004911 | DEAD box ATP-dependent RNA helicase | R/T/T | 1.35 |
| AAEL008042 | GPI mannosyltransferase 2 | DIV | 1.34 |
| AAEL002199 | N-acetyltransferase protein NAT1 | DIV | 1.34 |
| AAEL011943 | hairy protein | DIV | 1.34 |
| AAEL001233 | CLIPE9 | IMM | 1.34 |
| AAEL005669 | conserved hypothetical protein | UNK | 1.34 |
| AAEL002146 | putative surface layer protein | DIV | 1.33 |
| AAEL010606 | dsCAM | IMM | 1.33 |
| AAEL002756 | synaptotagmin-4, putative | TRP | 1.33 |
| AAEL003226 | pupal cuticle protein 78E, putative | C/S | 1.32 |
| AAEL005296 | fatty acyl-CoA reductase 2 | DIV | 1.32 |
| AAEL006167 | runt | DIV | 1.32 |
| AAEL014429 | t-box transcription factor tbx20 | R/T/T | 1.32 |
| AAEL009471 | syntaxin 4, putative | DIV | 1.31 |
| AAEL007437 | conserved hypothetical protein | UNK | 1.31 |
| AAEL015321 | 5-hydroxytryptamine receptor 1 | DIV | 1.3 |
| AAEL004648 | Homeobox protein ceh-10 | R/T/T | 1.3 |
| AAEL007169 | allatostatin receptor | DIV | 1.29 |
| AAEL003399 | cytochrome P450 | R/S/M | 1.29 |
| AAEL009863 | sodium/dicarboxylate cotransporter, putative | TRP | 1.29 |
| AAEL007441 | translocon-associated protein, gamma subunit | DIV | 1.28 |
| AAEL007408 | argonaut-like protein | DIV | 1.28 |
| AAEL012778 | protease m1 zinc metalloprotease | PROT | 1.28 |
| AAEL010117 | FBN35 | IMM | 1.27 |
| AAEL009658 | alpha,alpha-trehalase | MET | 1.27 |
| AAEL007563 | dual oxidase 1 | IMM | 1.26 |
| AAEL006355 | SCRC1 | IMM | 1.26 |
| AAEL008492 | Ankyrin repeat and fibronectin type-III | IMM | 1.26 |
| AAEL005876 | hypothetical protein | UNK | 1.26 |
| AAEL010333 | probable ribosome biogenesis protein | DIV | 1.25 |
| AAEL005979 | epithelial membrane protein | IMM | 1.25 |
| AAEL012627 | Ankyrin repeat and fibronectin type-III | IMM | 1.25 |
| AAEL011095 | n-acetylgalactosaminyltransferase | MET | 1.25 |
| AAEL011771 | hypothetical protein | UNK | 1.25 |
| AAEL006567 | max binding protein, mnt | DIV | 1.24 |
| AAEL010889 | GPI1 protein | DIV | 1.24 |
| AAEL014392 | tenascin C | DIV | 1.23 |
| Aaeg:N24616 | SOD | IMM | 1.23 |
| AAEL003325 | niemann-pick C1 | DIV | 1.22 |
| AAEL004898 | Tensin-1 | DIV | 1.22 |
| AAEL000087 | TEP22 | IMM | 1.22 |
| AAEL012684 | ribosome-associated membrane protein | R/T/T | 1.22 |
| AAEL004691 | ring finger | DIV | 1.21 |
| AAEL013038 | diacylglycerol kinase, alpha, beta, gamma | DIV | 1.21 |
| AAEL007116 | peritrophic matrix protein 14 | C/S | 1.2 |
| AAEL005850 | expressed protein (HR4) | DIV | 1.2 |
| AAEL002545 | vacuolar H+-ATPase v0 sector accessory subunit | DIV | 1.2 |
| AAEL006137 | SRPN19 | IMM | 1.2 |
| AAEL000264 | phopholipase d | MET | 1.2 |
| AAEL010226 | daughterless | R/S/M | 1.2 |
| AAEL006715 | DNA polymerase iota | R/T/T | 1.19 |
| AAEL010738 | sodium bicarbonate cotransporter | TRP | 1.19 |
| AAEL006606 | hypothetical protein | UNK | 1.19 |
| AAEL004275 | Osiris, putative | DIV | 1.18 |
| AAEL008905 | host cell factor C1 | MET | 1.18 |
| AAEL004358 | moira, isoform A | DIV | 1.17 |
| AAEL005057 | salivary gland-expressed bHLH | R/T/T | 1.17 |
| AAEL015259 | hypothetical protein | UNK | 1.17 |
| AAEL008346 | achaete-scute complex protein T3, putative | DIV | 1.16 |
| AAEL006633 | IAP2 | IMM | 1.16 |
| AAEL006092 | tartan (LRR) | IMM | 1.16 |
| AAEL005814 | Protogenin | DIV | 1.15 |
| AAEL008690 | apterous | DIV | 1.14 |
| AAEL011779 | Protein bric-a-brac 2 | DIV | 1.14 |
| AAEL008961 | Protein bowel | DIV | 1.14 |
| AAEL006877 | PPO4 | IMM | 1.14 |
| AAEL002460 | conserved hypothetical protein | UNK | 1.14 |
| AAEL005179 | hypothetical protein | UNK | 1.14 |
| AAEL015487 | zinc finger protein, putative | DIV | 1.13 |
| AAEL001569 | Cysteine string protein, putative | DIV | 1.13 |
| AAEL000098 | DNA-directed RNA polymerase II largest chain | R/T/T | 1.13 |
| AAEL007015 | conserved hypothetical protein | UNK | 1.13 |
| AAEL014041 | hypothetical protein | UNK | 1.12 |
| AAEL014047 | hypothetical protein | UNK | 1.12 |
| AAEL002644 | conserved hypothetical protein | UNK | 1.12 |
| AAEL014544 | PPO6 | IMM | 1.11 |
| AAEL001914 | SCRAC1 | IMM | 1.11 |
| AAEL003372 | Homeobox protein Hox-B1, putative | R/T/T | 1.11 |
| AAEL010148 | sodium/potassium-dependent atpase beta-2 subunit | TRP | 1.11 |
| AAEL010869 | hypothetical protein | UNK | 1.11 |
| AAEL004745 | pupal cuticle protein, putative | C/S | 1.1 |
| AAEL011716 | tartan (LRR) | IMM | 1.1 |
| AAEL014962 | conserved hypothetical protein | UNK | 1.1 |
| AAEL014356 | CTL2 | IMM | 1.09 |
| AAEL006693 | uroporphyrinogen decarboxylase | MET | 1.09 |
| AAEL006931 | Polyadenylate-binding protein-interacting protein | DIV | 1.08 |
| AAEL004883 | guanylate cyclase | DIV | 1.08 |
| AAEL003015 | protein phosphatase 2a, regulatory subunit | DIV | 1.08 |
| AAEL012227 | conserved hypothetical protein | UNK | 1.08 |
| AAEL008768 | multiprotein bridging factor, putative | DIV | 1.07 |
| AAEL009305 | numb-associated kinase | DIV | 1.07 |
| AAEL012566 | zinc finger protein Xfin-like | DIV | 1.07 |
| AAEL013430 | Rhodopsin, GQ-coupled, putative | DIV | 1.07 |
| AAEL006904 | stromal membrane-associated protein | DIV | 1.07 |
| AAEL005506 | salivary gland secretion 1 | DIV | 1.07 |
| AAEL006101 | carboxylesterase | R/S/M | 1.07 |
| AAEL014194 | conserved hypothetical protein | UNK | 1.07 |
| AAEL001479 | protoheme ix farnesyltransferase | DIV | 1.05 |
| AAEL002975 | hypothetical protein | UNK | 1.05 |
| AAEL014599 | hypothetical protein | UNK | 1.05 |
| AAEL009360 | serine/threonine protein kinase | DIV | 1.04 |
| AAEL007696 | REL1A | IMM | 1.04 |
| AAEL002327 | hypothetical protein | UNK | 1.04 |
| AAEL003236 | tetraspanin 39D | DIV | 1.03 |
| AAEL012570 | acid phosphatase SurE | DIV | 1.03 |
| AAEL006783 | histone h3 methyltransferase | R/T/T | 1.03 |
| AAEL006718 | multitransmembrane protein | DIV | 1.02 |
| Aaeg:N51900 | HSC70-3 | IMM | 1.02 |
| AAEL001666 | conserved hypothetical protein | UNK | 1.02 |
| AAEL008877 | conserved hypothetical protein | UNK | 1.02 |
| AAEL000041 | forkhead box protein (AaegFOXM2) | DIV | 1.01 |
| AAEL006300 | P53 induced protein | DIV | 1 |
| AAEL010987 | p15-2a protein, putative | DIV | 1 |
| AAEL001794 | TEP20 | IMM | 1 |
| AAEL009682 | serine collagenase 1 precursor, putative | IMM | 1 |
| AAEL000191 | conserved hypothetical protein | UNK | 1 |
| AAEL009519 | hypothetical protein | UNK | 1 |
| AAEL000745 | hypothetical protein | UNK | 1 |
| AAEL013866 | wdpeat protein | DIV | 0.99 |
| AAEL012128 | cationic amino acid transporter | TRP | 0.99 |
| AAEL008635 | abc transporter | TRP | 0.99 |
| AAEL004641 | PTS system IIA component domain-containing protein | DIV | 0.98 |
| AAEL010769 | SRPN6 | IMM | 0.98 |
| AAEL010347 | hypothetical protein | UNK | 0.98 |
| AAEL011903 | zinc finger protein | DIV | 0.97 |
| AAEL008015 | anon-194 | DIV | 0.97 |
| AAEL005381 | Dissatisfaction (Dsf) | DIV | 0.97 |
| AAEL010488 | ets | DIV | 0.97 |
| AAEL001405 | clathrin coat assembly protein | TRP | 0.97 |
| AAEL011777 | SRPN8 | IMM | 0.96 |
| AAEL013087 | mediator complex, 95kD-subunit, putative | DIV | 0.95 |
| AAEL001163 | TEP23 | IMM | 0.95 |
| AAEL003933 | DBLOX | IMM | 0.95 |
| AAEL005462 | hypothetical protein | UNK | 0.95 |
| AAEL007436 | gawky | DIV | 0.94 |
| AAEL003357 | DENN domain-containing protein 1A | DIV | 0.94 |
| AAEL014408 | m-phase inducer phosphatase(cdc25) | DIV | 0.94 |
| AAEL013303 | conserved hypothetical protein | UNK | 0.94 |
| AAEL010774 | glutamate receptor, putative | DIV | 0.93 |
| AAEL015437 | conserved hypothetical protein | UNK | 0.93 |
| AAEL002487 | P53 regulated pa26 nuclear protein sestrin | DIV | 0.92 |
| AAEL005643 | guanine nucleotide exchange factor | DIV | 0.92 |
| AAEL014518 | T-cell receptor beta chain | DIV | 0.92 |
| AAEL006674 | CLIPB29 | IMM | 0.92 |
| AAEL007829 | hypothetical protein | UNK | 0.92 |
| AAEL001672 | multicopper oxidase | DIV | 0.91 |
| AAEL000633 | TOLL8 | IMM | 0.91 |
| AAEL009338 | C-type lectin | IMM | 0.91 |
| AAEL003421 | triacylglycerol lipase, putative | MET | 0.91 |
| AAEL012897 | aconitase, mitochondrial | R/S/M | 0.91 |
| AAEL002067 | cytochrome P450 | R/S/M | 0.91 |
| AAEL003470 | nach, isoform A | DIV | 0.9 |
| AAEL004546 | coatomer beta subunit | DIV | 0.9 |
| AAEL013409 | dsCAM | IMM | 0.89 |
| AAEL015119 | cuticle protein, putative | C/S | 0.88 |
| AAEL007589 | Dedicator of cytokinesis protein 2 | DIV | 0.88 |
| AAEL009257 | mitochondrial cytochrome c oxidase subunit | DIV | 0.88 |
| AAEL013488 | glucose inhibited division protein a | DIV | 0.88 |
| AAEL012267 | TEP13 | IMM | 0.88 |
| AAEL007139 | sugar transporter | TRP | 0.87 |
| AAEL010643 | hypothetical protein | UNK | 0.87 |
| AAEL006502 | conserved hypothetical protein | UNK | 0.87 |
| AAEL001149 | serine-enriched protein | DIV | 0.86 |
| AAEL011167 | cathepsin l | IMM | 0.86 |
| AAEL012958 | Leucine Rich Repeats (LRR) | IMM | 0.86 |
| AAEL010898 | conserved hypothetical protein | UNK | 0.86 |
| AAEL009565 | conserved hypothetical protein | UNK | 0.86 |
| AAEL012396 | conserved hypothetical protein | UNK | 0.86 |
| AAEL000856 | germ cell-less protein | DIV | 0.85 |
| AAEL009517 | lumican, putative | DIV | 0.85 |
| AAEL015522 | f-box and wd40 domain protein 7 | DIV | 0.85 |
| AAEL003857 | DEFD | IMM | 0.85 |
| AAEL014327 | conserved hypothetical protein | UNK | 0.85 |
| AAEL007940 | hypothetical protein | UNK | 0.85 |
| AAEL014925 | cuticular protein 58, RR-2 family | C/S | 0.84 |
| AAEL002012 | Phosphorylated adaptor for RNA export, putative | TRP | 0.84 |
| AAEL009551 | TOLL11 | IMM | 0.83 |
| AAEL004625 | conserved hypothetical protein | UNK | 0.83 |
| AAEL003267 | conserved hypothetical protein | UNK | 0.83 |
| AAEL011458 | cuticular protein 111, RR-3 family | C/S | 0.82 |
| AAEL002866 | cyclin l | DIV | 0.82 |
| AAEL012711 | CLIPC12 | IMM | 0.82 |
| AAEL001428 | transcription factor Hairy, putative | R/T/T | 0.82 |
| AAEL002526 | conserved hypothetical protein | UNK | 0.82 |
| AAEL014043 | hypothetical protein | UNK | 0.82 |
| AAEL008970 | cuticular protein 141, RR-2 family | C/S | 0.81 |
| AAEL010231 | aegyptin 2 | DIV | 0.81 |
| AAEL000866 | beta-lactamase | DIV | 0.81 |
| AAEL014710 | alpha-amylase | DIG | 0.81 |
| AAEL009905 | DNA polymerase subunit alpha B | R/T/T | 0.81 |
| AAEL013764 | wdpeat protein | DIV | 0.8 |
| AAEL006095 | Gelsolin precursor | DIV | -0.8 |
| AAEL010300 | conserved hypothetical protein | UNK | -0.8 |
| AAEL004555 | hypothetical protein | UNK | -0.8 |
| AAEL004057 | gastrula zinc finger protein XFG20-1 | DIV | -0.81 |
| AAEL015442 | zinc finger protein | DIV | -0.81 |
| AAEL007006 | CLIP17A | IMM | -0.81 |
| AAEL003541 | GALE1 | IMM | -0.81 |
| AAEL005185 | Lucine Rich Repeats (LRR) | IMM | -0.81 |
| AAEL000354 | dimeric dihydrodiol dehydrogenase | MET | -0.81 |
| AAEL002461 | RNA-binding protein, putative | R/T/T | -0.81 |
| AAEL012771 | metalloproteinase | DIV | -0.82 |
| AAEL002734 | zinc finger protein | DIV | -0.82 |
| AAEL008676 | Acylglycerol kinase, mitochondrial | DIV | -0.82 |
| AAEL008646 | fibrinogen and fibronectin | DIV | -0.82 |
| AAEL009100 | mitochondrial oxodicarboxylate carrier | R/S/M | -0.82 |
| AAEL014150 | transcription factor IIIA, putative | R/T/T | -0.82 |
| AAEL000685 | conserved hypothetical protein | UNK | -0.82 |
| AAEL013402 | hypothetical protein | UNK | -0.82 |
| AAEL003989 | GTP-binding protein alpha subunit, gna | DIV | -0.83 |
| AAEL007765 | SRPN10 | IMM | -0.83 |
| AAEL002378 | carboxylesterase | R/S/M | -0.83 |
| AAEL007859 | conserved hypothetical protein | UNK | -0.83 |
| AAEL002168 | conserved hypothetical protein | UNK | -0.83 |
| AAEL008867 | zinc finger protein | DIV | -0.84 |
| AAEL012229 | zinc finger protein | DIV | -0.84 |
| AAEL006583 | multicopper oxidase | DIV | -0.84 |
| AAEL007926 | retinoid-inducible serine carboxypeptidase (serine carboxypeptidase | MET | -0.84 |
| AAEL014493 | aldehyde oxidase | R/S/M | -0.84 |
| AAEL010293 | hypothetical protein | UNK | -0.84 |
| AAEL004789 | hypothetical protein | UNK | -0.84 |
| AAEL008802 | conserved hypothetical protein | UNK | -0.84 |
| AAEL000640 | alanine-glyoxylate aminotransferase | DIV | -0.85 |
| AAEL008556 | prp4 | DIV | -0.85 |
| AAEL002161 | conserved hypothetical protein | UNK | -0.85 |
| AAEL013417 | FBN24 | IMM | -0.85 |
| AAEL005194 | FBN26 | IMM | -0.85 |
| AAEL003642 | serine protease | IMM | -0.85 |
| AAEL005262 | conserved hypothetical protein | UNK | -0.85 |
| AAEL013498 | PPO1 | IMM | -0.86 |
| AAEL012994 | glucose-6-phosphate isomerase | MET | -0.86 |
| AAEL013798 | cytochrome P450 | R/S/M | -0.86 |
| AAEL005071 | GTP binding protein | R/T/T | -0.86 |
| AAEL012981 | sugar transporter | TRP | -0.86 |
| AAEL004100 | hypothetical protein | UNK | -0.86 |
| AAEL006949 | yellow-e | IMM | -0.87 |
| AAEL007473 | cytochrome P450 | R/S/M | -0.87 |
| AAEL002541 | cystinosin | TRP | -0.87 |
| AAEL009985 | conserved hypothetical protein | UNK | -0.87 |
| AAEL013934 | hypothetical protein | UNK | -0.87 |
| AAEL000258 | conserved hypothetical protein | UNK | -0.87 |
| AAEL003709 | crotonobetainyl-CoA-hydratase, putative | MET | -0.88 |
| AAEL002813 | coupling factor, putative | R/S/M | -0.88 |
| AAEL004989 | RNA-binding protein | R/T/T | -0.88 |
| AAEL013885 | conserved hypothetical protein | UNK | -0.88 |
| AAEL007162 | gaba(a) receptor-associated protein | C/S | -0.89 |
| AAEL000221 | mediator complex, subunit, putative | DIV | -0.89 |
| AAEL010671 | oxidoreductase | R/S/M | -0.89 |
| AAEL009529 | transient receptor potential cation channel | TRP | -0.89 |
| AAEL009195 | Major facilitator superfamily domain-containing protein | DIV | -0.9 |
| AAEL002098 | DNA repair endonuclease xp-f / mei-9 / rad1 | R/T/T | -0.9 |
| AAEL004820 | zinc finger protein | DIV | -0.91 |
| AAEL011838 | U5 snRNP-specific protein | DIV | -0.91 |
| AAEL007865 | Protein FAM40A | DIV | -0.91 |
| AAEL003253 | CLIP13B | IMM | -0.91 |
| AAEL014724 | clip-domain serine protease, putative | IMM | -0.91 |
| AAEL011400 | FREP36 | IMM | -0.91 |
| AAEL011379 | coenzyme q10 biosynthesis protein | MET | -0.91 |
| AAEL014607 | cytochrome P450 | R/S/M | -0.91 |
| AAEL001186 | hypothetical protein | UNK | -0.91 |
| AAEL004713 | E3 ubiquitin-protein ligase TRIM37 | DIV | -0.92 |
| AAEL001877 | fucosyltransferase 11 (fut11) | DIV | -0.92 |
| AAEL000111 | nitrilase, putative | MET | -0.92 |
| AAEL003596 | hypothetical protein | UNK | -0.92 |
| AAEL013126 | putative protein G12 | DIV | -0.93 |
| AAEL012646 | mucin-like protein 1 | DIV | -0.93 |
| AAEL003938 | mRNA cleavage stimulating factor, 50kD-subunit, putative | DIV | -0.93 |
| AAEL012266 | cytochrome P450 | R/S/M | -0.93 |
| AAEL003552 | DNA-directed RNA polymerase subunit rpb6 | R/T/T | -0.93 |
| AAEL003467 | conserved hypothetical protein | UNK | -0.93 |
| AAEL000322 | hypothetical protein | UNK | -0.93 |
| AAEL006859 | Myb-interacting protein, putative | DIV | -0.94 |
| AAEL007288 | dynamin | DIV | -0.94 |
| AAEL011050 | Activating signal cointegrator 1 complex subunit | DIV | -0.94 |
| AAEL002662 | elongase, putative | MET | -0.94 |
| AAEL004056 | conserved hypothetical protein | UNK | -0.94 |
| AAEL002770 | hypothetical protein | UNK | -0.94 |
| AAEL008719 | Sm protein G, putative | DIV | -0.95 |
| AAEL004823 | MnSOD1 | IMM | -0.95 |
| AAEL004868 | hemomucin | IMM | -0.95 |
| AAEL005258 | syntaxin, putative | TRP | -0.95 |
| AAEL008580 | conserved hypothetical protein | UNK | -0.95 |
| AAEL001705 | odorant response protein ODR-4, putative | CSR | -0.97 |
| AAEL008171 | double-stranded RNA-binding protein zn72d | DIV | -0.97 |
| AAEL006936 | suppressor of cytokine signaling 7 | IMM | -0.97 |
| AAEL006704 | FREP18 | IMM | -0.97 |
| AAEL006317 | short-chain dehydrogenase | R/S/M | -0.97 |
| AAEL001615 | mitochondrial ribosomal protein, S18C, putative | R/S/M | -0.97 |
| AAEL007809 | potassium channel regulator | TRP | -0.97 |
| AAEL003264 | conserved hypothetical protein | UNK | -0.97 |
| AAEL011362 | gastrula zinc finger protein XFG20-1 | DIV | -0.98 |
| AAEL010083 | IMD | IMM | -0.98 |
| AAEL012471 | DOME | IMM | -0.98 |
| AAEL010582 | glutathione-s-transferase theta, gst | R/S/M | -0.98 |
| AAEL007946 | glutathione-s-transferase theta, gst | R/S/M | -0.98 |
| AAEL000196 | conserved hypothetical protein | UNK | -0.98 |
| AAEL001020 | anterior fat body protein | DIV | -0.99 |
| AAEL002593 | serine protease | IMM | -0.99 |
| AAEL013725 | conserved hypothetical protein | UNK | -0.99 |
| AAEL005344 | ras association domain protein, putative | DIV | -1.01 |
| AAEL008609 | zinc carboxypeptidase | PROT | -1.01 |
| AAEL005474 | hypothetical protein | UNK | -1.01 |
| AAEL004891 | zinc finger protein Ci-ZF(U1like)-9 | DIV | -1.02 |
| AAEL011607 | CTLMA14 | IMM | -1.02 |
| AAEL002861 | conserved hypothetical protein | UNK | -1.02 |
| AAEL003540 | bicoid-interacting protein 3 | DIV | -1.03 |
| AAEL006066 | peptidyl-trna hydrolase | DIV | -1.03 |
| AAEL004025 | glucose dehydrogenase | TRP | -1.03 |
| AAEL002722 | protein kinase C inhibitor, putative | DIV | -1.04 |
| AAEL013935 | conserved hypothetical protein | UNK | -1.04 |
| AAEL003444 | caspase-s19 | IMM | -1.05 |
| AAEL011334 | tequila, isoform C | C/S | -1.06 |
| AAEL003486 | zinc finger protein 383 | DIV | -1.06 |
| AAEL008568 | glucosyl/glucuronosyl transferases | MET | -1.06 |
| AAEL008559 | glutaminase | MET | -1.06 |
| AAEL010837 | scaf6, isoform A | DIV | -1.07 |
| AAEL011992 | NADH:ubiquinone dehydrogenase, putative | R/S/M | -1.07 |
| AAEL006255 | conserved hypothetical protein | UNK | -1.07 |
| AAEL005428 | conserved hypothetical protein | UNK | -1.08 |
| AAEL009219 | predicted protein | C/S | -1.09 |
| AAEL010199 | Activating signal cointegrator 1 | DIV | -1.09 |
| AAEL002680 | AMP dependent ligase | MET | -1.09 |
| AAEL011206 | aminoacylase, putative | PROT | -1.1 |
| AAEL011081 | conserved hypothetical protein | UNK | -1.1 |
| AAEL014811 | conserved hypothetical protein | UNK | -1.1 |
| AAEL012524 | AMP dependent ligase | MET | -1.11 |
| AAEL005601 | conserved hypothetical protein | UNK | -1.11 |
| AAEL012938 | zinc finger protein | DIV | -1.13 |
| AAEL011596 | mitotic checkpoint serine/threonine-protein kinase bub1 and bubr1 | DIV | -1.13 |
| AAEL008550 | zinc finger protein, putative | DIV | -1.13 |
| AAEL006953 | mucin-like peritrophin | C/S | -1.14 |
| AAEL002739 | zinc finger protein, putative | DIV | -1.14 |
| AAEL009244 | serine-type enodpeptidase, putative | PROT | -1.14 |
| AAEL011520 | sucrose transport protein | TRP | -1.14 |
| AAEL000647 | predicted protein | DIG | -1.16 |
| Aaeg:N41092 | CTL | IMM | -1.16 |
| AAEL010921 | organic anion transporter | TRP | -1.16 |
| AAEL005766 | fructose-bisphosphate aldolase | DIV | -1.17 |
| AAEL013676 | 26S protease regulatory subunit | DIV | -1.17 |
| AAEL009680 | chymotrypsin, putative | IMM | -1.17 |
| AAEL014605 | cytochrome P450 | R/S/M | -1.17 |
| AAEL008557 | conserved hypothetical protein | UNK | -1.19 |
| AAEL006467 | alcohol dehydrogenase | MET | -1.2 |
| AAEL011834 | SNARE protein TLG2/syntaxin 16 | DIV | -1.21 |
| AAEL000165 | salivary Cys-rich secreted peptide | DIV | -1.21 |
| AAEL007705 | hect E3 ubiquitin ligase | DIV | -1.21 |
| AAEL012025 | mitochondrial ribosomal protein, L45, putative | R/S/M | -1.21 |
| AAEL005026 | ATP-dependent bile acid permease | TRP | -1.22 |
| AAEL003160 | conserved hypothetical protein | UNK | -1.24 |
| AAEL002783 | mitochondrial ribosomal protein, L37, putative | R/S/M | -1.25 |
| AAEL002238 | hypothetical protein | UNK | -1.26 |
| AAEL015451 | hypothetical protein | UNK | -1.32 |
| AAEL004126 | sterol desaturase | MET | -1.34 |
| AAEL007432 | serine collagenase 1 precursor, putative | IMM | -1.35 |
| AAEL008119 | hypothetical protein | UNK | -1.35 |
| AAEL010673 | NADH dehydrogenase, putative | R/S/M | -1.38 |
| AAEL012902 | heterogeneous nuclear ribonucleoprotein, putative | R/T/T | -1.38 |
| AAEL004240 | gamma glutamyl transpeptidases | MET | -1.41 |
| AAEL012685 | Juvenile hormone-inducible protein | DIV | -1.48 |
| AAEL012532 | tetraspanin 29fb | DIV | -1.5 |
| AAEL002263 | conserved hypothetical protein | UNK | -1.69 |
| AAEL004369 | alpha-glucosidase | DIG | -1.73 |
| AAEL003426 | sodium-dependent phosphate transporter | TRP | -1.77 |
| AAEL003849 | DEFE | IMM | -1.83 |
| AAEL000667 | alpha-amylase | DIG | -1.87 |
