# Supplementary material for: Transcriptome Analysis of Aedes aegypti Transgenic Mosquitoes with Altered Immunity
Source: PLoS Pathog. 2011 Nov 17;7(11):e1002394. doi: 10.1371/journal.ppat.1002394 (PMC3219725; doi:10.1371/journal.ppat.1002394)
Supplement: Table S8 — Hierarchical clustering of genes regulated by P. gallinaceum in the fat bodies (PgFB), RNAi PIAS depletion, REL1 and REL2 fat body-specific ectopic expression. Abbreviations are: IMM, immunity; R/S/M, redox, stress and mitochondrion;; DIG, digestive; C/S, cytoskeletal and structural; PROT, proteolysis; TRP, transport; R/T/T, replication, transcription, and translation; MET, metabolism; DIV, diverse functions; UNK, unknown functions. (DOCX) [file ppat.1002394.s013.docx]

Table S8. Hierarchical clustering of genes regulated by *P. gallinaceum* in the fat bodies (PgFB), RNAi PIAS depletion, REL1 and REL2 fat body-specific ectopic expression. Abbreviations are: IMM, immunity; R/S/M, redox, stress and mitochondrion;; DIG, digestive; C/S, cytoskeletal and structural; PROT, proteolysis; TRP, transport; R/T/T, replication, transcription, and translation; MET, metabolism; DIV, diverse functions; UNK, unknown functions.

|  |  |  | Logfold | | | |  |
| --- | --- | --- | --- | --- | --- | --- | --- |
| GENE ID | Name | FC | PgFB | PIAS | REL1 | REL2 | Cluster |
| AAEL011206 | aminoacylase, putative | PROT | -1.10 | -0.18 | -0.96 | -0.32 | I |
| AAEL001705 | odorant response protein ODR-4 | CSR | -0.97 | -0.13 | -0.68 | -0.04 | I |
| AAEL014107 | conserved hypothetical protein | UNK | -0.52 | -0.18 | -0.36 | -0.07 | I |
| AAEL008171 | double-stranded RNA-binding protein zn72d | DIV | -0.97 | -0.40 | -0.65 | -0.46 | I |
| AAEL007705 | hect E3 ubiquitin ligase | DIV | -1.21 | -0.26 | -0.85 | -0.62 | I |
| AAEL004868 | hemomucin | IMM | -0.95 | -0.22 | -0.51 | -0.37 | I |
| AAEL000111 | nitrilase, putative | MET | -0.92 | -0.07 | -0.53 | -0.52 | I |
| AAEL006467 | alcohol dehydrogenase | MET | -1.20 | -0.19 | -0.45 | -0.54 | I |
| AAEL000221 | mediator complex, subunit, putative | DIV | -0.89 | -0.48 | -0.88 | -0.35 | I |
| AAEL001020 | anterior fat body protein | DIV | -0.99 | -0.55 | -1.04 | -0.26 | I |
| AAEL014903 | 40S ribosomal protein S24 | R/T/T | -0.53 | -0.27 | -0.53 | -0.15 | I |
| AAEL010406 | hypothetical protein | UNK | -0.64 | -0.53 | -0.62 | -0.17 | I |
| AAEL012930 | CCR4-NOT transcription complex subunit 3 | DIV | -0.61 | -0.35 | -0.57 | -0.35 | I |
| AAEL002378 | carboxylesterase | R/S/M | -0.83 | -0.41 | -0.44 | -0.06 | I |
| AAEL010837 | Calcium homeostasis endoplasmic reticulum protein | DIV | -1.07 | -0.61 | -0.47 | -0.26 | I |
| AAEL007865 | Protein FAM40A | DIV | -0.91 | -0.44 | -0.40 | -0.18 | I |
| AAEL013676 | 26S protease regulatory subunit | DIV | -1.17 | -0.44 | -0.52 | -0.29 | I |
| AAEL004891 | zinc finger protein Ci-ZF(U1like)-9 | DIV | -1.02 | -0.33 | -0.38 | -0.24 | I |
| AAEL006317 | short-chain dehydrogenase | R/S/M | -0.97 | -0.48 | -0.41 | -0.47 | I |
| AAEL014605 | cytochrome P450 | R/S/M | -1.17 | -0.82 | -0.44 | -0.84 | I |
| AAEL005843 | conserved hypothetical protein | UNK | -0.68 | -0.56 | -0.22 | -0.40 | I |
| AAEL008557 | conserved hypothetical protein | UNK | -1.19 | -1.02 | -0.38 | -0.34 | I |
| AAEL001427 | short-chain dehydrogenase | R/S/M | -0.63 | -0.48 | -0.27 | -0.13 | I |
| AAEL004989 | RNA-binding protein | R/T/T | -0.88 | -0.62 | -0.13 | -0.30 | I |
| AAEL004789 | hypothetical protein | UNK | -0.84 | -0.57 | -0.06 | -0.25 | I |
| AAEL002739 | zinc finger protein, putative | DIV | -1.14 | -1.09 | -0.09 | -0.35 | I |
| AAEL010169 | ankyrin repeats | DIV | -0.21 | -0.29 | -0.17 | -0.04 | I |
| AAEL011379 | coenzyme q10 biosynthesis protein | MET | -0.91 | -0.82 | -0.11 | -0.05 | I |
| AAEL011462 | transcription initiation factor IIE beta subunit | R/T/T | -0.27 | -0.31 | -0.10 | 0.03 | I |
| AAEL006936 | suppressor of cytokine signaling 7 | IMM | -0.97 | -0.37 | -0.32 | -0.90 | I |
| AAEL006949 | suppressor of cytokine signaling 7 | IMM | -0.87 | -0.26 | -0.22 | -0.84 | I |
| AAEL004126 | sterol desaturase | MET | -1.34 | -0.48 | -0.27 | -0.93 | I |
| AAEL003709 | crotonobetainyl-CoA-hydratase, putative | MET | -0.88 | -0.47 | -0.13 | -0.72 | I |
| AAEL006514 | sodium-dependent phosphate transporter | TRP | -0.53 | -0.14 | 0.06 | -0.38 | I |
| AAEL012025 | mitochondrial ribosomal protein, L45, putative | R/S/M | -1.21 | -0.36 | -0.29 | 0.07 | I |
| AAEL002861 | conserved hypothetical protein | UNK | -1.02 | -0.39 | -0.24 | 0.06 | I |
| AAEL010673 | NADH dehydrogenase, putative | R/S/M | -1.38 | -0.32 | -0.18 | -0.06 | I |
| AAEL000165 | salivary Cys-rich secreted peptide | DIV | -1.21 | -0.58 | -0.05 | 0.01 | I |
| AAEL013498 | prophenoloxidase | IMM | -0.86 | -0.31 | -0.04 | 0.02 | I |
| AAEL003552 | DNA-directed RNA polymerase subunit rpb6 | R/T/T | -0.93 | -0.37 | -0.22 | -0.18 | I |
| AAEL004198 | conserved hypothetical protein | UNK | -0.40 | -0.17 | -0.11 | -0.09 | I |
| AAEL009922 | conserved hypothetical protein | UNK | -0.46 | -0.26 | -0.12 | -0.11 | I |
| AAEL002168 | conserved hypothetical protein | UNK | -0.83 | -0.39 | -0.17 | -0.10 | I |
| AAEL002722 | protein kinase C inhibitor, putative | DIV | -1.04 | -0.47 | -0.15 | -0.14 | I |
| AAEL010812 | zinc finger protein | DIV | -0.54 | -0.26 | -0.06 | -0.07 | I |
| AAEL010083 | IMD | IMM | -0.98 | -0.33 | -0.03 | -0.14 | I |
| AAEL007946 | glutathione-s-transferase theta, gst | R/S/M | -0.98 | -0.34 | 0.07 | -0.19 | I |
| AAEL011992 | NADH:ubiquinone dehydrogenase, putative | R/S/M | -1.07 | -0.33 | 0.00 | -0.05 | I |
| AAEL007765 | serine protease inhibitor 4, serpin-4 | IMM | -0.83 | -0.25 | 0.07 | -0.04 | I |
| AAEL004100 | hypothetical protein | UNK | -0.86 | -0.22 | 0.03 | -0.19 | I |
| AAEL011520 | sucrose transport protein | TRP | -1.14 | -0.23 | 0.12 | -0.24 | I |
| AAEL008119 | hypothetical protein | UNK | -1.35 | -0.64 | -0.03 | -0.38 | I |
| AAEL004056 | conserved hypothetical protein | UNK | -0.94 | -0.56 | 0.01 | -0.25 | I |
| AAEL010582 | glutathione-s-transferase theta, gst | R/S/M | -0.98 | -0.43 | 0.16 | -0.16 | I |
| AAEL001355 | conserved hypothetical protein | UNK | -0.48 | -0.25 | 0.05 | -0.18 | I |
| AAEL003938 | mRNA cleavage stimulating factor, 50kD-subunit, putative | DIV | -0.93 | -0.36 | 0.09 | -0.40 | I |
| AAEL007162 | gaba(a) receptor-associated protein | C/S | -0.89 | -0.48 | -0.96 | -0.95 | I |
| AAEL000258 | conserved hypothetical protein | UNK | -0.87 | -0.18 | -0.86 | -0.79 | I |
| AAEL008802 | conserved hypothetical protein | UNK | -0.84 | -0.46 | -0.64 | -0.85 | I |
| AAEL009100 | mitochondrial oxodicarboxylate carrier | R/S/M | -0.82 | -0.45 | -0.56 | -0.60 | I |
| AAEL007103 | p37NB protein, putative(LRR) | IMM | -0.44 | -0.16 | -1.07 | -0.77 | I |
| AAEL004390 | peroxinectin | IMM | -0.50 | -0.34 | -1.23 | -1.44 | I |
| AAEL013338 | lethal(2)essential for life protein, l2efl | DIV | -0.73 | -0.71 | -1.89 | -2.81 | I |
| AAEL004524 | proacrosin, putative | MET | -0.06 | -0.07 | -0.14 | -0.23 | I |
| AAEL004386 | peroxinectin | IMM | -0.13 | -0.26 | -1.30 | -2.03 | I |
| AAEL004388 | peroxinectin | IMM | -0.15 | -0.37 | -1.23 | -2.16 | I |
| AAEL004401 | peroxinectin | IMM | 0.01 | -0.23 | -0.93 | -2.35 | I |
| AAEL003467 | conserved hypothetical protein | UNK | -0.93 | -0.15 | -0.94 | -4.10 | I |
| AAEL013229 | tubulin alpha chain | C/S | -0.12 | -0.18 | -0.23 | -0.68 | I |
| AAEL009166 | putative protein G12 | DIV | -0.72 | 1.31 | -1.13 | -3.69 | I |
| AAEL011608 | peptidoglycan recognition protein-1, putative | IMM | -0.29 | 0.30 | -0.30 | -0.96 | I |
| AAEL008397 | glutathione peroxidase | R/S/M | -0.16 | 0.13 | -0.17 | -0.81 | I |
| AAEL001488 | ribosomal protein L15 | R/T/T | -0.41 | 0.09 | -0.45 | -0.96 | I |
| AAEL011400 | FREP36 | IMM | -0.91 | -0.15 | -1.01 | -1.64 | I |
| AAEL010576 | modifier of mdg4 | DIV | -0.16 | -0.03 | -0.18 | -0.26 | I |
| AAEL000322 | hypothetical protein | UNK | -0.93 | -0.24 | -0.40 | -1.35 | I |
| AAEL014837 | prophenoloxidase | IMM | -0.23 | -0.51 | -0.16 | -0.32 | I |
| AAEL012510 | NFkappaB essential modulator, putative | DIV | -0.10 | -0.32 | -0.04 | -0.13 | I |
| AAEL000760 | serine protease | IMM | -0.06 | -0.26 | -0.09 | -0.33 | I |
| AAEL013501 | prophenoloxidase | IMM | -0.07 | -0.49 | 0.03 | -0.65 | I |
| AAEL010819 | Vacuolar ATP synthase subunit H | TRP | -0.14 | -0.46 | 0.19 | -0.55 | I |
| AAEL003153 | zinc finger protein 25 | DIV | -0.77 | -0.78 | -0.03 | -0.80 | I |
| AAEL002216 | 5-amp-activated protein kinase, beta subunit | DIV | -0.42 | -0.34 | 0.07 | -0.33 | I |
| AAEL005930 | ubiquitin-protein ligase | DIV | -0.37 | -0.49 | 0.02 | -0.23 | I |
| AAEL003934 | conserved hypothetical protein | UNK | -0.29 | -0.47 | 0.25 | -0.26 | I |
| AAEL007185 | synbindin | DIV | -0.22 | -0.19 | 0.17 | -0.54 | I |
| AAEL011621 | SPL3 | IMM | -0.19 | -0.18 | 0.18 | -0.51 | I |
| AAEL002665 | matrix metalloproteinase | PROT | -0.42 | -0.25 | 0.62 | -0.67 | I |
| AAEL008609 | zinc carboxypeptidase | PROT | -1.01 | -0.50 | 0.90 | -0.99 | II |
| AAEL013936 | serine protease inhibitor, serpin | IMM | 0.00 | 0.07 | 0.33 | -1.77 | II |
| AAEL008646 | fibrinogen and fibronectin | DIV | -0.82 | 0.28 | -0.13 | -0.50 | II |
| AAEL012771 | metalloproteinase | DIV | -0.82 | 0.18 | -0.10 | -0.25 | II |
| AAEL008898 | sulfotransferase (sult) | DIV | -0.40 | 0.23 | -0.10 | -0.07 | II |
| AAEL005605 | adenylsulfate kinase | DIV | -0.18 | 0.25 | 0.45 | -0.20 | II |
| AAEL003889 | gram-negative bacteria binding protein | IMM | -0.51 | 0.38 | 0.85 | 0.18 | II |
| AAEL000256 | epithelial membrane protein | IMM | -0.15 | -0.19 | 0.11 | 0.06 | II |
| AAEL000099 | serine protease | IMM | -0.16 | -0.15 | 0.19 | 0.07 | II |
| AAEL002680 | AMP dependent ligase | MET | -1.09 | -0.42 | 0.71 | -0.14 | II |
| AAEL000059 | proacrosin, putative | MET | -0.35 | -0.21 | 0.84 | 0.29 | II |
| AAEL012471 | protein tyrosine phosphatase, putative | IMM | -0.98 | -0.41 | 1.98 | -0.33 | II |
| AAEL011634 | fibrinogen and fibronectin | IMM | -0.19 | -0.53 | -0.26 | 0.28 | II |
| AAEL011764 | prophenoloxidase | IMM | -0.19 | -0.54 | 0.03 | 0.26 | II |
| AAEL001328 | hypothetical protein | UNK | -0.44 | -0.36 | -0.09 | 0.41 | II |
| AAEL007374 | yellow protein precursor, putative | DIV | -0.21 | -0.32 | -0.07 | 0.39 | II |
| AAEL011009 | fibrinogen and fibronectin | IMM | -0.23 | -0.26 | 0.01 | 0.29 | II |
| AAEL001615 | mitochondrial ribosomal protein, S18C, putative | R/S/M | -0.97 | -0.47 | -0.39 | 0.60 | II |
| AAEL002238 | hypothetical protein | UNK | -1.26 | -0.65 | -0.40 | 0.19 | II |
| AAEL002783 | mitochondrial ribosomal protein, L37, putative | R/S/M | -1.25 | -0.55 | -0.31 | 0.35 | II |
| AAEL004823 | superoxide dismutase, Mn | IMM | -0.95 | -0.27 | -0.07 | 0.19 | II |
| AAEL008676 | Acylglycerol kinase, mitochondrial | DIV | -0.82 | -0.28 | -0.01 | 0.13 | II |
| AAEL010199 | Activating signal cointegrator 1 | DIV | -1.09 | -0.38 | -0.01 | 0.30 | II |
| AAEL012685 | Juvenile hormone-inducible protein | DIV | -1.48 | -0.74 | 0.03 | 0.84 | II |
| AAEL007809 | potassium channel regulator | TRP | -0.97 | -0.60 | 0.08 | 0.43 | II |
| AAEL011050 | Activating signal cointegrator 1 complex subunit | DIV | -0.94 | -0.48 | 0.09 | 0.28 | II |
| AAEL003541 | galectin | IMM | -0.81 | 0.09 | -0.09 | 0.06 | II |
| AAEL005194 | fibrinogen and fibronectin | IMM | -0.85 | -0.03 | -0.02 | 0.26 | II |
| AAEL007288 | dynamin | DIV | -0.94 | 0.14 | 0.08 | 0.36 | II |
| AAEL002593 | serine protease | IMM | -0.99 | 0.20 | 0.26 | 0.19 | II |
| AAEL003347 | CRAL/TRIO domain-containing protein | TRP | -0.42 | -0.07 | 0.08 | 0.49 | II |
| AAEL011607 | galactose-specific C-type lectin, putative | IMM | -1.02 | -0.24 | 0.92 | 2.24 | II |
| AAEL003642 | serine protease | IMM | -0.85 | 0.43 | 0.27 | 1.42 | II |
| AAEL003253 | clip-domain serine protease, putative | IMM | -0.91 | -0.18 | 0.83 | 0.84 | II |
| AAEL006890 | conserved hypothetical protein | UNK | -0.17 | 0.44 | -0.75 | -0.47 | III |
| AAEL002730 | serine protease inhibitor (serpin-4), putative | IMM | -0.01 | 0.32 | -0.37 | 0.00 | III |
| AAEL002207 | conserved hypothetical protein | UNK | -0.06 | 0.20 | -0.22 | 0.01 | III |
| AAEL007306 | alpha-actinin | C/S | -0.32 | 0.35 | -0.23 | -0.53 | III |
| AAEL010737 | aromatic amino acid decarboxylase | IMM | -0.05 | 0.51 | -0.09 | -0.49 | III |
| AAEL009423 | cd36 antigen | IMM | -0.08 | 0.41 | -0.07 | -0.33 | III |
| AAEL000610 | proboscipedia, isoform D | DIV | -0.16 | 0.58 | -0.24 | -0.10 | III |
| AAEL006569 | bat5 hla-b-associated transcript | DIV | -0.23 | 0.81 | 0.08 | -0.30 | III |
| AAEL013299 | serine protease, putative | IMM | 0.02 | 0.64 | -0.02 | 0.07 | III |
| AAEL013171 | oxidase/peroxidase | IMM | 0.15 | 0.56 | -0.01 | 0.01 | III |
| AAEL010276 | aminomethyltransferase | DIV | 0.17 | 0.37 | 0.02 | 0.02 | III |
| AAEL006721 | 2-oxoglutarate dehydrogenase | R/S/M | -0.13 | 0.34 | 0.01 | 0.28 | III |
| AAEL008319 | protein disulfide isomerase | DIV | -0.13 | 0.28 | 0.02 | 0.09 | III |
| AAEL003697 | serine protease inhibitor, serpin | IMM | 0.04 | 0.51 | 0.33 | 0.21 | III |
| AAEL011774 | sarcolemmal associated protein, putative | DIV | 0.74 | 0.61 | -0.22 | -0.37 | III |
| AAEL008635 | abc transporter | TRP | 0.99 | 0.97 | -0.16 | -0.57 | III |
| AAEL001569 | Cysteine string protein, putative | DIV | 1.13 | 0.68 | -0.25 | -0.37 | III |
| AAEL009360 | serine/threonine protein kinase | DIV | 1.04 | 0.57 | 0.00 | -0.36 | III |
| AAEL013747 | phosphodiesterase 6 | DIV | 0.54 | 0.36 | 0.11 | -0.25 | III |
| AAEL009473 | conserved hypothetical protein | UNK | 2.09 | 1.46 | 0.07 | -0.34 | III |
| AAEL014392 | tenascin C | DIV | 1.23 | 0.81 | 0.08 | -0.24 | III |
| AAEL015259 | hypothetical protein | UNK | 1.17 | 0.93 | 0.14 | -0.30 | III |
| AAEL004174 | t-box transcription factor tbx6 | R/T/T | 1.51 | 1.10 | 0.28 | -0.30 | III |
| AAEL000700 | cadherin | C/S | 1.66 | 1.89 | -0.12 | -0.26 | III |
| AAEL007744 | hypothetical protein | UNK | 1.82 | 1.97 | -0.09 | -0.35 | III |
| AAEL014931 | sarm1 | DIV | 0.54 | 0.58 | 0.05 | -0.14 | III |
| AAEL008961 | Protein bowel | DIV | 1.14 | 1.30 | 0.31 | -0.07 | III |
| AAEL005745 | neurokinin-3 receptor, putative | TRP | 2.32 | 2.06 | 0.37 | -0.25 | III |
| AAEL012897 | aconitase, mitochondrial | R/S/M | 0.91 | 0.25 | -0.17 | -0.62 | III |
| AAEL010500 | glutathione-s-transferase theta, gst | R/S/M | 1.65 | 0.46 | -0.17 | -0.50 | III |
| AAEL009954 | Niemann-Pick Type C-2, putative | IMM | 1.60 | 0.32 | -0.05 | -0.34 | III |
| AAEL006832 | frizzled, putative | DIV | 1.79 | 0.32 | 0.03 | -0.37 | III |
| AAEL005416 | oxidase/peroxidase | IMM | 1.41 | 0.33 | 0.03 | -0.37 | III |
| AAEL002545 | vacuolar H+-ATPase v0 sector accessory subunit | DIV | 1.20 | 0.52 | 0.02 | -0.28 | III |
| AAEL009382 | nucleolar protein 66 | DIV | 1.63 | 0.59 | 0.04 | -0.42 | III |
| AAEL009863 | sodium/dicarboxylate cotransporter, putative | TRP | 1.29 | 0.43 | 0.08 | -0.30 | III |
| AAEL007940 | hypothetical protein | UNK | 0.85 | 0.39 | 0.12 | -0.32 | III |
| AAEL005578 | conserved hypothetical protein | UNK | 0.65 | 0.23 | 0.14 | -0.22 | III |
| AAEL006167 | runt | DIV | 1.32 | 0.44 | 0.17 | -0.41 | III |
| AAEL000264 | phopholipase d | MET | 1.20 | 0.27 | 0.02 | -0.48 | III |
| AAEL006674 | clip-domain serine protease, putative | IMM | 0.92 | 0.18 | 0.10 | -0.45 | III |
| AAEL006931 | Polyadenylate-binding protein-interacting protein | DIV | 1.08 | 0.12 | 0.23 | -0.34 | III |
| AAEL011403 | hypothetical protein | UNK | 1.57 | 0.22 | -0.03 | 0.17 | III |
| AAEL012711 | trypsin, putative | IMM | 0.82 | 0.08 | 0.00 | 0.07 | III |
| AAEL014348 | caspase-1 | IMM | 1.41 | 0.15 | 0.01 | 0.26 | III |
| AAEL009531 | niemann-pick C1 | DIV | 1.94 | -0.06 | -0.03 | -0.03 | III |
| AAEL003844 | galectin | IMM | 1.85 | -0.08 | -0.02 | 0.26 | III |
| AAEL010171 | peptidoglycan recognition protein sb2 | IMM | 1.63 | -0.07 | 0.08 | 0.19 | III |
| AAEL009257 | mitochondrial cytochrome c oxidase subunit | DIV | 0.88 | 0.01 | 0.22 | 0.11 | III |
| AAEL000204 | conserved hypothetical protein | UNK | 1.68 | -0.13 | -0.07 | -0.23 | III |
| AAEL013748 | C-type lectin | IMM | 1.85 | -0.33 | -0.01 | -0.26 | III |
| AAEL007945 | eukaryotic translation initiation factor 3 subunit | R/T/T | 1.40 | -0.19 | 0.04 | -0.26 | III |
| AAEL001914 | scavenger receptor, putative | IMM | 1.11 | -0.03 | 0.07 | -0.21 | III |
| AAEL005988 | lysozyme | IMM | 1.84 | -0.09 | 0.10 | -0.38 | III |
| AAEL014699 | abc transporter | TRP | 1.82 | 0.06 | 0.09 | -0.36 | III |
| AAEL006355 | SCRC1 | IMM | 1.26 | -0.31 | 0.10 | 0.10 | III |
| AAEL004583 | conserved hypothetical protein | UNK | 1.68 | -0.24 | 0.17 | 0.11 | III |
| AAEL014548 | peroxiredoxins, prx-1, prx-2, prx-3 | IMM | 1.63 | -0.23 | 0.17 | -0.20 | III |
| AAEL002136 | zinc finger protein | DIV | 1.69 | -0.35 | 0.28 | -0.32 | III |
| AAEL011217 | PQ loop repeat-containing protein 3 | DIV | 2.08 | -0.46 | 0.41 | -0.10 | III |
| AAEL013184 | open rectifier K[+] channel 1, isoform B | DIV | 2.19 | 1.13 | -0.10 | -0.08 | III |
| AAEL007053 | receptor protein kinase, putative | DIV | 1.95 | 1.16 | 0.10 | 0.02 | III |
| AAEL004116 | hypothetical protein | UNK | 2.37 | 1.42 | 0.23 | -0.09 | III |
| AAEL007437 | conserved hypothetical protein | UNK | 1.31 | 0.75 | 0.15 | -0.16 | III |
| AAEL011265 | abc transporter | TRP | 1.71 | 0.87 | 0.14 | -0.28 | III |
| AAEL002979 | conserved hypothetical protein | UNK | 1.54 | 0.77 | 0.17 | -0.18 | III |
| AAEL014408 | m-phase inducer phosphatase(cdc25) | DIV | 0.94 | 0.48 | 0.08 | 0.09 | III |
| AAEL000902 | sugar transporter | TRP | 1.46 | 0.77 | 0.20 | 0.11 | III |
| AAEL009155 | conserved hypothetical protein | UNK | 1.79 | 0.68 | 0.15 | 0.11 | III |
| AAEL014526 | sideroflexin 1,2,3 | DIV | 2.11 | 0.85 | 0.16 | 0.02 | III |
| AAEL002327 | hypothetical protein | UNK | 1.04 | 0.29 | -0.02 | -0.01 | III |
| AAEL004112 | peroxiredoxins, prx-1, prx-2, prx-3 | IMM | 2.37 | 0.63 | -0.02 | 0.07 | III |
| AAEL005265 | conserved hypothetical protein | UNK | 2.32 | 0.79 | -0.02 | -0.07 | III |
| AAEL004625 | conserved hypothetical protein | UNK | 0.83 | 0.34 | 0.01 | -0.07 | III |
| AAEL004149 | hypothetical protein | UNK | 1.91 | 0.72 | 0.13 | -0.21 | III |
| AAEL000393 | suppressors of cytokine signalling | IMM | 1.71 | 0.54 | 0.11 | -0.09 | III |
| AAEL002625 | mucin-like peritrophin | C/S | 1.94 | 0.53 | 0.17 | -0.20 | III |
| AAEL006633 | inhibitor of apoptosis 1, diap1 | IMM | 1.16 | 0.26 | 0.07 | -0.08 | III |
| AAEL012927 | hypothetical protein | UNK | 2.21 | 0.42 | 0.14 | -0.20 | III |
| AAEL002453 | zinc finger protein | DIV | 1.65 | 0.36 | 0.07 | -0.20 | III |
| AAEL004373 | hypothetical protein | UNK | 2.02 | 0.47 | 0.09 | -0.31 | III |
| AAEL004114 | UNC93A protein, putative | DIV | 2.14 | 0.49 | 0.16 | -0.38 | III |
| AAEL014892 | cytochrome P450 | R/S/M | 2.24 | 0.23 | 0.23 | -0.09 | III |
| AAEL004546 | coatomer beta subunit | DIV | 0.90 | 0.17 | 0.17 | 0.02 | III |
| AAEL012595 | lipoprotein NlpD | DIV | 1.36 | 0.18 | 0.23 | -0.05 | III |
| AAEL004168 | syntaxin | TRP | 1.78 | 0.30 | 0.34 | -0.13 | III |
| AAEL003161 | adenylosuccinate synthetase | DIV | 1.38 | 0.28 | 0.21 | -0.19 | III |
| AAEL004354 | hypothetical protein | UNK | 2.01 | 0.48 | 0.36 | -0.13 | III |
| AAEL011213 | conserved hypothetical protein | UNK | 1.89 | 0.49 | 0.37 | -0.09 | III |
| AAEL009905 | DNA polymerase subunit alpha B | R/T/T | 0.81 | 0.20 | 0.27 | 0.05 | III |
| AAEL010987 | p15-2a protein, putative | DIV | 1.00 | 0.38 | 0.28 | 0.07 | III |
| AAEL005575 | transient receptor potential channel 4, putative | TRP | 1.63 | 0.50 | 0.31 | 0.15 | III |
| AAEL010769 | serine protease inhibitor, serpin | IMM | 0.98 | 0.41 | 0.24 | -0.14 | III |
| AAEL002644 | conserved hypothetical protein | UNK | 1.12 | 0.51 | 0.28 | -0.20 | III |
| AAEL000098 | DNA-directed RNA polymerase II largest chain | R/T/T | 1.13 | 0.42 | 0.32 | -0.23 | III |
| AAEL015522 | f-box and wd40 domain protein 7 | DIV | 0.85 | 0.22 | 0.36 | -0.08 | III |
| AAEL005850 | expressed protein (HR4) | DIV | 1.20 | 0.60 | 0.05 | 0.29 | III |
| AAEL003267 | conserved hypothetical protein | UNK | 0.83 | 0.38 | 0.09 | 0.18 | III |
| AAEL007833 | fatty acid desaturase | DIV | 0.66 | 0.48 | 0.02 | 0.15 | III |
| AAEL007015 | conserved hypothetical protein | UNK | 1.13 | 0.76 | 0.22 | 0.23 | III |
| AAEL005381 | Dissatisfaction (Dsf) | DIV | 0.97 | 0.69 | 0.23 | 0.27 | III |
| AAEL008306 | mitogen activated protein kinase kinase kinase 5, mapkkk5, mekk5 | DIV | 1.67 | 1.01 | 0.24 | 0.06 | III |
| AAEL004167 | zinc finger protein | DIV | 1.48 | 0.91 | 0.28 | 0.05 | III |
| AAEL013797 | Kinesin-like protein CG14535 | DIV | 1.72 | 1.27 | 0.30 | 0.08 | III |
| AAEL001479 | protoheme ix farnesyltransferase | DIV | 1.05 | 0.53 | 0.29 | 0.10 | III |
| AAEL012754 | hypothetical protein | UNK | 0.50 | 0.23 | 0.20 | 0.20 | III |
| AAEL007696 | embryonic polarity dorsal | IMM | 1.04 | 0.61 | 0.93 | -0.03 | III |
| AAEL006854 | Niemann-Pick Type C-2, putative | IMM | 0.14 | 0.44 | -0.04 | -0.22 | III |
| AAEL011070 | C-type lectin, galactose-binding | IMM | 0.15 | 0.39 | 0.03 | -0.51 | III |
| AAEL003064 | Sugar phosphate exchanger 2 | DIV | 0.48 | 0.54 | 0.18 | -0.47 | III |
| AAEL001561 | conserved hypothetical protein | UNK | 0.30 | 0.28 | 0.30 | -0.39 | III |
| AAEL013175 | connector enhancer of ksr | DIV | 0.13 | 0.44 | 0.23 | -0.24 | IV |
| AAEL007587 | putative 11.9 kDa salivary protein | IMM | -0.01 | -0.51 | -0.01 | -0.04 | IV |
| AAEL009051 | peroxiredoxin 6, prx-6 | IMM | -0.01 | -0.33 | 0.12 | 0.08 | IV |
| AAEL013499 | prophenoloxidase | IMM | 0.32 | -0.42 | -0.05 | 0.01 | IV |
| AAEL005293 | galectin | IMM | 0.44 | -0.53 | -0.01 | 0.14 | IV |
| AAEL012135 | galectin | IMM | 0.27 | -0.73 | 0.12 | 0.05 | IV |
| AAEL002693 | venom allergen | MET | 0.21 | -0.31 | 0.23 | 0.03 | IV |
| AAEL004041 | flotillin-2 | DIV | 0.28 | -0.47 | -0.08 | -0.24 | IV |
| AAEL011734 | leucinech transmembrane protein | IMM | 0.20 | -0.18 | -0.02 | -0.17 | IV |
| AAEL002615 | leucinech transmembrane protein | IMM | 0.50 | -0.28 | 0.11 | -0.40 | IV |
| AAEL012234 | cytoplasmic dynein intermediate chain, (dhic) | DIV | 0.20 | -0.31 | 0.18 | -0.22 | IV |
| AAEL009032 | phosphatidylinositol transfer protein SEC14 | DIV | 0.31 | -0.57 | 0.24 | -0.32 | IV |
| AAEL012078 | putative potassium antiporter CHAC-1 | DIV | 0.11 | -0.38 | 0.35 | -0.33 | IV |
| AAEL010148 | sodium/potassium-dependent atpase beta-2 subunit | TRP | 1.11 | -0.23 | 0.01 | -0.42 | IV |
| AAEL006001 | conserved hypothetical protein | UNK | 0.75 | -0.01 | 0.05 | -0.48 | IV |
| AAEL009294 | phosphatidylinositol 4-kinase | R/T/T | 0.41 | -0.25 | 0.27 | -0.28 | IV |
| AAEL004426 | Syntaxin 18, putative | DIV | 0.69 | -0.25 | 0.29 | -0.16 | IV |
| AAEL011610 | galactose-specific C-type lectin, putative | IMM | -0.03 | 0.15 | 0.10 | 1.10 | IV |
| AAEL011622 | serine protease, putative | IMM | 0.08 | 0.33 | 0.16 | 0.82 | IV |
| AAEL007624 | relish | IMM | 1.42 | 0.06 | 0.23 | 1.49 | IV |
| AAEL007748 | cd36 antigen | IMM | 0.31 | 0.28 | 0.19 | 0.35 | IV |
| AAEL001794 | macroglobulin/complement | IMM | 1.00 | 0.49 | 0.85 | 1.93 | IV |
| AAEL000958 | conserved hypothetical protein | UNK | 0.05 | -0.17 | 0.23 | 0.33 | IV |
| AAEL014385 | galactose-specific C-type lectin | IMM | -0.09 | -0.33 | 0.31 | 1.33 | IV |
| AAEL009909 | cln3/battenin | DIV | 0.14 | -0.22 | 0.15 | 0.51 | IV |
| AAEL003832 | Defensin-C | IMM | 1.84 | -0.84 | 1.34 | 3.49 | IV |
| AAEL011777 | serine protease inhibitor, serpin | IMM | 0.96 | -0.53 | 0.85 | 0.87 | IV |
| AAEL001163 | macroglobulin/complement | IMM | 0.95 | -0.04 | 0.32 | 0.47 | IV |
| AAEL014349 | serine protease | IMM | 1.58 | 0.21 | 0.92 | 1.33 | IV |
| AAEL014755 | tep2 | IMM | 1.50 | 0.20 | 0.93 | 0.83 | IV |
| AAEL000087 | macroglobulin/complement | IMM | 1.22 | -0.07 | 1.02 | 1.05 | IV |
| AAEL002720 | serine protease inhibitors, serpins | IMM | -0.09 | 0.29 | 0.45 | 0.81 | IV |
| AAEL012785 | lumbrokinase-3(1) precursor, putative | MET | -0.11 | 0.08 | 0.40 | 0.54 | IV |
| AAEL003686 | serine protease inhibitor, serpin | IMM | -0.03 | 0.11 | 0.62 | 0.88 | IV |
| AAEL007593 | lumbrokinase-3(1) precursor, putative | MET | -0.07 | 0.14 | 0.53 | 0.37 | IV |
| AAEL002629 | serine protease | IMM | 0.14 | 0.45 | 0.81 | 0.57 | IV |
| AAEL002301 | serine protease | IMM | 0.13 | 0.31 | 0.90 | 0.72 | IV |
| AAEL000074 | serine protease | IMM | 0.08 | 0.11 | 0.64 | 0.08 | IV |
| AAEL005064 | serine protease | IMM | -0.10 | 0.31 | 0.91 | 0.24 | IV |
| AAEL000709 | developmental protein cactus | IMM | -0.04 | -0.13 | 1.09 | 0.47 | IV |
| AAEL011407 | type II transmembrane receptor OtB7, putative | IMM | 0.06 | -0.33 | 1.14 | 0.78 | IV |
