# Supplementary material for: Transcriptome Analysis of Aedes aegypti Transgenic Mosquitoes with Altered Immunity
Source: PLoS Pathog. 2011 Nov 17;7(11):e1002394. doi: 10.1371/journal.ppat.1002394 (PMC3219725; doi:10.1371/journal.ppat.1002394)
Supplement: Table S9 — Primers used for gene expression knockdowns and for quantitative Real-Time PCR. (DOCX) [file ppat.1002394.s014.docx]

Table S9. Primers used for gene expression knockdowns and for Real-Time PCR

| Real-time PCR primer | |  |
| --- | --- | --- |
| gene | left | right |
| caspS18 | CCTTTCTCGACCTACTTGCG | CGATCCTTATCAGTGCCGTT |
| caspS19 | TCGTTTCTCGTGCTGCTCTA | TATCGGCTCCATTCCTTTTG |
| runx2 | CCGTGTGCCTTCAAGGTTAT | ATCGCCGTACAATTCCGTAG |
| TPX2 | GCCGACAAGTCCATGAAGAT | CTGGCGGAGATTCTGCTTAC |
| TEP20 | GCGTTGATTCAGGTTACCGT | ATTACCGCCTTTGTTCGATG |
| FREP36 | TGGAGGTTTTGGAAGATTCG | TGCTCTTCATAGATGCACCG |
| IMD | TGGTCAACCTGTTATGGCAA | GGGTTGACTTTGTCGTCGTT |
| AAEL003426 | CAGTTTGGTATGTCGCATGG | CACGTTCCTGTTCCTTGGAT |
| AAEL002263 | TCTTATCTACGCCGACAGCA | CGGAGGCAAATACTGGAAAA |
| AAEL013126 | GATTGAGCGGATTCGTTGAT | GAATTCCGGACTGGTCTTCA |
| AAEL006583 | GTCCTACAGCGTGAATGGGT | ATAATGTGCTCCAAATCGGC |
| AAEL000667 | GTGTGGCATGAAGGAAAGGT | GAACCGAACTCCATTTCCAA |
| AAEL009166 | GCTTGAAAGCAGGAGGTTTG | TCAGGGCTGGTCTTCAGTTT |
| FREP13 | GGAGATCCAGCAATTCCAAA | TGAACTTGCTTCTGTCGGTG |
| TAK1 | ACAAAGCCAAATGGCAAAAC | CGTCTTGTGCAAGCTCCATA |
| ClipB39 | CACGCAAGCTGATAGCACAT | AGTCCGAATACGTCGGAATG |
| ClipB41 | TACCAACGGATAGCGGAAAC | TTTTCGATTTCAATCAGCCC |
| AAEL015418 | GATCGCTACCTGGCAATCAT | TGGATCAGGGTGGAGAAGAC |
| AAEL03426 | CAGTTTGGTATGTCGCATGG | CACGTTCCTGTTCCTTGGAT |
| AAEL06154 | GCTGTGGACCTCAGGAAGAG | GGTTGGTGTTGGTCGGTATC |
| AAEL09166 | GCTTGAAAGCAGGAGGTTTG | TCAGGGCTGGTCTTCAGTTT |
| DefA | AACTGCCGGAGGAAACCTAT | AATGCAATGAGCAGCACAAG |
| DefC | GCCTCAGTGCAATCTTCACA | CGTTTCAAGCGGAAGTTTTC |
| Vitellogenin | CCAGAAGACGTGAGCATTCA | TGGCGCAGATGATAGAACAG |
| REL2 | TGAATGTGCTGTTGGGTCAT | TTTTTACACATCACCGCCAA |
| RPS7 | TCAGTGTACAAGAAGCTGACCGGA | TTCCGCGCGCGCTCACTTATTAGATT |
| Gene Expression knock-down |  |  |
| T7-Cactus | T7-TTCGATTCCGGTGTGGATC | T7-TTCAGGAGTTCTTTTGCGC |
| T7-PIAS | T7-GATACACCCAGCAAGCCAGT | T7-AACCCCGCTATTGTATGCTG |
| T7-Caspar | T7-CAGCCACTTGAAGCATTTGA | T7-TCGTAGTCCAGCGTTGTGAG |
|  |  |  |
