# Supplementary material for: Transcriptome Analysis of Aedes aegypti Transgenic Mosquitoes with Altered Immunity
Source: PLoS Pathog. 2011 Nov 17;7(11):e1002394. doi: 10.1371/journal.ppat.1002394 (PMC3219725; doi:10.1371/journal.ppat.1002394)
Supplement: Table S10 — Validation of microarray expression data by means of quantitative Real-time PCR. PgFB – Plasmodium affected fat body transcriptome; PgMD - Plasmodium affected midgut transcriptome. (DOCX) [file ppat.1002394.s015.docx]

Table S10 Validation of microarray expression data by means of quantitative Real-time PCR.

PgFB – Plasmodium affected fat body transcriptome; PgMD - Plasmodium affected midgut transcriptome.

|  | gene | Real-time PCR | Microarray |
| --- | --- | --- | --- |
| PgFB | caspS18 | 1.42 | 1.71 |
| PgFB | caspS19 | -0.62 | -1.05 |
| PgFB | runx2 | 1.32 | 1.32 |
| PgFB | TPX2 | 1.74 | 2.37 |
| PgFB | TEP20 | 0.41 | 1.00 |
| PgFB | FREP36 | -0.41 | -0.91 |
| PgFB | IMD | -0.20 | -0.98 |
| PgFB | AAEL003426 | -1.30 | -1.77 |
| PgFB | AAEL002263 | -1.44 | -1.69 |
| PgFB | AAEL013126 | -0.66 | -0.93 |
| PgFB | AAEL006583 | -0.75 | -0.84 |
| PgFB | AAEL000667 | -1.16 | -1.87 |
| PgMD | TPX2 | 0.11 | 1.58 |
| PgMD | AAEL009166 | -0.33 | -2.74 |
| PgMD | FREP13 | -0.44 | -0.80 |
| PgMD | caspS18 | 0.42 | 1.06 |
| PgMD | TAK1 | -0.15 | -0.98 |
| PgMD | ClipB39 | 1.13 | 1.41 |
| PgMD | ClipB41 | 0.86 | 1.10 |
| PgMD | AAEL015418 | 0.90 | 1.93 |
| PgMD | AAEL03426 | -2.56 | -2.03 |
| PgMD | AAEL06154 | 0.87 | 1.56 |
| PgMD | AAEL09166 | -1.34 | -2.74 |
